# Analyzing the genetic diversity and biotechnological potential of *Leuconostoc pseudomesenteroides* by comparative genomics

**DOI:** 10.3389/fmicb.2022.1074366

**Published:** 2023-01-11

**Authors:** Ismail Gumustop, Fatih Ortakci

**Affiliations:** BioEngineering Department, Faculty of Life and Natural Sciences, Abdullah Gul University, Kayseri, Turkey

**Keywords:** *Leuconostoc pseudomesenteroides*, comparative genomics, CRISPR-Cas, mobile genetic elements, genetic diversity

## Abstract

*Leuconostoc pseudomesenteroides* is a lactic acid bacteria species widely exist in fermented dairy foods, cane juice, sourdough, kimchi, apple dumpster, caecum, and human adenoid. In the dairy industry, *Ln. pseudomesenteroides* strains are usually found in mesophilic starter cultures with lactococci. This species plays a crucial role in the production of aroma compounds such as acetoin, acetaldehyde, and diacetyl, thus beneficially affecting dairy technology. We performed genomic characterization of 38 *Ln. pseudomesenteroides* from diverse ecological niches to evaluate this species’ genetic diversity and biotechnological potential. A mere ~12% of genes conserved across 38 *Ln. pseudomesenteroides* genomes indicate that accessory genes are the driving force for genotypic distinction in this species. Seven main clades were formed with variable content surrounding mobile genetic elements, namely plasmids, transposable elements, IS elements, prophages, and CRISPR-Cas. All but three genomes carried CRISPR-Cas system. Furthermore, a type IIA CRISPR-Cas system was found in 80% of the CRISPR-Cas positive strains. AMBR10, CBA3630, and MGBC116435 were predicted to encode bacteriocins. Genes responsible for citrate metabolism were found in all but five strains belonging to cane juice, sourdough, and unknown origin. On the contrary, arabinose metabolism genes were only available in nine strains isolated from plant-related systems. We found that *Ln. pseudomesenteroides* genomes show evolutionary adaptation to their ecological environment due to niche-specific carbon metabolism and forming closely related phylogenetic clades based on their isolation source. This species was found to be a reservoir of type IIA CRISPR-Cas system. The outcomes of this study provide a framework for uncovering the biotechnological potential of *Ln. pseudomesenteroides* and its future development as starter or adjunct culture for dairy industry.

## 1. Introduction

*Leuconostoc (Ln) pseudomesenteroides* is gram-positive, catalase-negative, pyrrolidonyl arylamidase-negative, leucine aminopeptidase-negative, vancomycin-resistant non-motile lactic acid bacteria (LAB) species that form lenticular or spherical cells with dimensions of approximately (0.5 to 0.7 μm) × (0.7–1.2 μm) and usually occurs in short chains or pairs. They grow in MRS and produce gas; however, no growth occurs in the 6.5% NaCl broth (Farrow et al., 1989; Cappelli et al., 1999).

Most *Ln. pseudomesenteroides* strains do not form pigments, and they grow at both 10 and 37°C. Acid (presumably lactic acid) is produced from the fermentation of D-xylose, trehalose, ribose, raffinose, a-methyl-D-glucoside, melibiose, D-mannose, maltose, D-glucose, D-fructose, and N-acetylglucosamine. Most strains of *Ln. pseudomesenteroides* produce acid from D-turanose, sucrose, lactose, gentiobiose, galactose, esculin, cellobiose, and L-arabinose; however, few strains produce acid from 5-keto-gluconate, amygdalin, 2-keto-gluconate, and arbutin. No acid formation occurs from adonitol, D-arabinose, D-arabitol, L-arabitol, dulcitol, erythritol, D-fucose, L-fucose, glycerol, inositol, inulin, D-lyxose, melezitose, a-methyl-D-mannoside, L-rhamnose, L-sorbose, sorbitol, D-tagatose, L-xylose, or xylitol. *Leuconostoc pseudomesenteroides* is negative for arginine dehydrolase, urease, L-isoleucine arylamidase, and L-proline arylamidase; however, positive for beta-galactosidase and beta-D-xylosidase. The G + C content, as measured by the thermal denaturation method, ranges between 38.1–40.8 mol%. The isolation sources of *Ln. pseudomesenteroides* were clinical sources, dairy sources, and foods. The NCDO 768 strain isolated from cane juice produces acid from lactose, gluconate, gentiobiose, galactose, 5-keto-gluconate, 2-keto-gluconate, cellobiose, and L-arabinose however, no acid production from starch, salicin, mannitol, arbutin, and amygdalin was reported (Farrow et al., 1989; Cappelli et al., 1999).

One of the primary phenotypic differences between *Ln. mesenteroides* and *Ln. pseudomesenteroides* is their acid production capability from mannitol and starch, with the former producing acid from mannitol but not from starch upon 7 days of incubation; however, *Ln. pseudomesenteroides* shows the opposite reaction. Moreover, strains of *Ln. pseudomesenteroides* are negative for L-proline arylamidase and L-isoleucine arylamidase. *Leuconostoc pseudomesenteroides* and *Ln. citreum* strains are easy to distinguish from the remaining *Leuconostoc* species, as reported by Farrow et al. (1989). Both *Ln. pseudomesenteroides* and *Ln. mesenteroides* can convert fructose into mannitol, a low-calorie sugar metabolized independently of insulin that could substitute glucose, fructose, lactose, or sucrose in foods (Hemme and Foucaud-Scheunemann, 2004).

*Leuconostoc pseudomesenteroides* has widely been found in fermented food systems such as fermented dairy products, wine, olives, kimchi, beans, cacao, and meat (Ludbrook et al., 1997; De Bellis et al., 2010; Nieto-Arribas et al., 2010; Kim et al., 2011; Mesas et al., 2011; Papalexandratou et al., 2011). In dairy starter culture technology, *Ln. pseudomesenteroides* strains exist in mesophilic starter culture formulations in conjunction with *Ln. mesenteroides* and lactococci species. The presence of *Ln. pseudomesenteroides* is instrumental for various technological traits. For instance, due to the heterofermentative carbohydrate fermentation capacity of this species, CO_2_ gas releases from the pentose phosphate pathway, which leads to desired open texture in blue-veined cheese so that aerobic *Penicillium roqueforti* could colonize and proliferate in the cheese. In Gouda-type cheese, *Ln. pseudomesenteroides* produces diacetyl by degrading citrate and contributes to eye formation by its heterofermentative lifestyle (Hemme and Foucaud-Scheunemann, 2004). This species also produces dextrans that contribute to food systems’ textural attributes and sensorial properties by improving their viscosity and final stability (Duboc and Mollet, 2001). Moreover, the bioproduction of aromatic compounds such as acetoin, acetaldehyde, and more importantly diacetyl further emphasizes *Ln. pseudomesenteroides*’ contribution to organoleptic attributes in several fermented dairy foods (Vedamuthu, 1994; Hemme and Foucaud-Scheunemann, 2004; Nieto-Arribas et al., 2010).

Since *Ln. pseudomesenteroides* is a versatile LAB species and has widely been isolated from diverse food sources, comparative genomics of *Ln. pseudomesenteroides* strains will contribute to our understanding of the adaptation of this species to fermented foods. Thus, the present study aimed to mine insights into the evolution, environmental adaptation, and biotechnological potential of *Ln. pseudomesenteroides* isolated from different ecological niches.

The limited studies regarding *Ln. pseudomesentreoides* have led to relatively limited knowledge with regard to genomic diversity at the species level. In order to completely uncover the potential of *Ln. pseudomesenteroides*, we should evaluate genetic diversity within the species and define strains of industrial and scientific interest. In this study, we evaluated 38 strains including the type strain FDAARGOS_1003 through comparative genomic analyses to establish the genetic diversity of the overall species and their biotechnological potential.

## 2. Materials and methods

### 2.1. Annotation and genetic diversity analysis

A total of 40 *Ln. pseudomesenteroides* strains were acquired from the NCBI GenBank database (Clark et al., 2016). CheckM tool was utilized to determine the quality of genome assemblies (Parks et al., 2015). Thirty-eight genomes were annotated with Prokka (Seemann, 2014) with the following arguments – kingdom Bacteria – compliant. Identification of core- and pangenomes and presence/absence of genes across all strains were performed by feeding Roary (Page et al., 2015) with GFF files from Prokka using the following arguments: -e -n -v -r. Open/close classification of the pangenome was determined by fitting Heap’s law model with 10,000 permutations by micropan (Snipen and Liland, 2020) package. Evaluation of the similarity between genomes by presence/absence of genes was performed with principal coordinate analysis (PCoA) in R (version 4.1.1; R Core Team, 2021) by calculating Jaccard distance with prabclus (Hennig and Hausdorf, 2020) package. Neighbor joining phylogenetic analysis of whole genome alignment provided TYGS (Meier-Kolthoff and Göker, 2019). The phylogenetic tree of the whole genome alignment was built with the iTOL web tool (Letunic and Bork, 2021). Core orthogroups shared between *Ln. pseudomesenteroides* strains were annotated to clusters of orthologous groups (COG) categories using eggNOG-mapper (Huerta-Cepas et al., 2017).

### 2.2. Identification of genetic potentials

Identification of clustered regularly interspaced short palindromic repeat (CRISPR) elements and Cas enzyme clusters were performed by the CRISPRCasFinder (Couvin et al., 2018) web tool, and CRISPRviz (Nethery and Barrangou, 2019) was utilized to detect spacer and repeat sequences and their alignment. Putative carbohydrate-active enzyme (CAZyme) domains were identified by using the HMMER (Potter et al., 2018) tool on the dbCAN database (Zhang et al., 2018; v10) according to protocol dbCAN. Using default settings, putative prophage sequences were identified with the PHASTER (Hennig and Hausdorf, 2020) tool. Detection of potential bacteriocin-like sequences was performed with the BAGEL4 (van Heel et al., 2018) web tool, and the detected sequences were validated with the NCBI’s BLASTP (Cantarel et al., 2009) suite. Screening of antimicrobial resistance genes was performed with the Comprehensive Antibiotic Resistance Database (CARD; Alcock et al., 2020) web tool with perfect hits only. PLSDB (Galata et al., 2019; Schmartz et al., 2022) web tool was used to identify plasmid sequences with default settings. ISfinder (Siguier et al., 2006) web tool was utilized for detecting insertion sequences in *Ln. pseudomesenteroides* genomes by adjusting the e-value threshold to 0.01. Horizontally transferred sequences were identified with COLOMBO (Waack et al., 2006) tool. Annotation of interspersed repeats and low-complexity sequences was performed with RepeatMasker (Smit and Rubley, 2008) tool. Putative secondary metabolite gene clusters were screened using antiSMASH (Blin et al., 2021).

## 3. Results

### 3.1. Genome characteristics and genetic diversity

**Figure 1:**
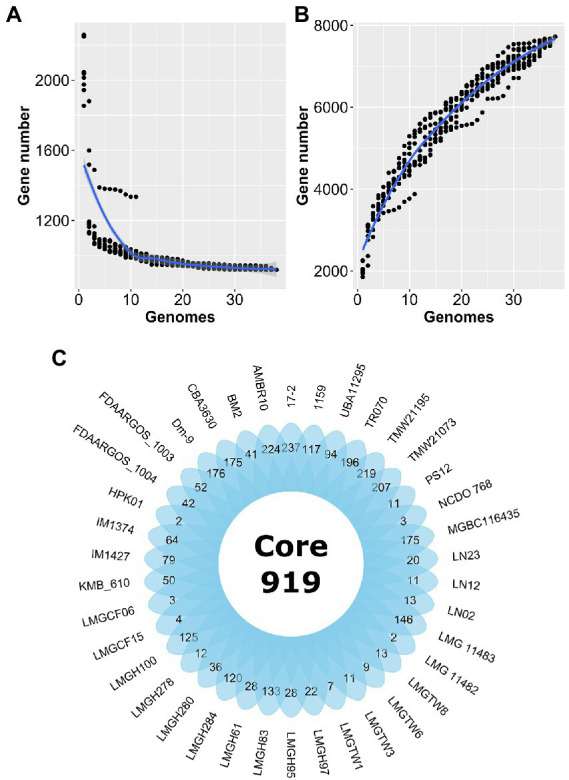
Estimation of the core genome **(A)** and pangenome **(B)** of 38 *Leuconostoc pseudomesenteroides* strains by including genomes one by one. R programming language (R Core Team, 2021) and ggplot2 (Wickham, 2016) package were used to plot the graph. **(C)** Venn diagram representing the core and unique gene families of *Ln. pseudomesenteroides* obtained by MCL clustering algorithm analyses.

Forty *Ln. pseudomesenteroides* genome assemblies retrieved from NCBI GenBank were quality-checked using CheckM (Parks et al., 2015). Table 1 shows the 38 *Ln. pseudomesenteroides* genomes (i.e., LMGCF08 and 4882 were discarded due to their low-quality CheckM outputs) isolated from different ecological niches including dairy, cheese starter culture, sourdough, kimchi, apple dumpster, cane juice, caecum, and human adenoid representing a broad range of ecological environments. The genome sizes of the strains ranged from 1.81 to 2.32 Mb (average 2.04 Mb). The G + C content of each strain slightly deviated and ranged from 38.5 and 39.2% (average 39%), which is consistent with the reference strain FDAARGOS_1003 (Table 1). Total CDS in each genome ranged from 1,825 to 2,359 proposing variability across *Ln. pseudomesenteroides* genomes (Li et al., 2021). To approximate the overall gene pool of *Ln. pseudomesenteroides*, we calculated the core genome (Figure 1A) and pangenome (Figure 1B) based on 38 strains. A total of 7,724 COGs were estimated, and the pangenome curve showed an asymptotic trend that did not plateau in 38 genomes implying new genes were still identified. Therefore, the pangenome of *Ln. pseudomesenteroides* is open (Figure 1B). Core genome analysis of 38 *Ln. pseudomesenteroides* strains revealed that the number of shared COGs reduced with an increase in the number of sequenced genomes. A total of 919 COGs were identified that were present in the core genome of all 38 strains, which represent ~12% of the entire pangenome (Figure 1B). In addition, a total of 6,805 variable COGs were determined, of which 2,907 of them were characterized as unique (Figure 1C). Across all *Ln. pseudomesenteroides* strains screened, 17–2 carried the unique COGs of 237 (Figure 1C).

**Table 1:** Genome statistics of 38 *Leuconostoc pseudomesenteroides* strains studied in the present work.

Strain	GenBank accession	Isolation source	Sequencing technology	Size (Mb)	GC%	CDS	Plasmid
1159	GCA_000686465.1	Mesophilic cheese starter cultures	Illumina MiSeq	2.04	39	2100	4
17–2	GCA_014634725.1	Sourdough	Illumina NovaSeq	2.27	38.5	2263	0
AMBR10	GCA_901830415.1	Human adenoid	N/A	2.3	39.2	2359	1
BM2	GCA_002092535.1	Dairy	Illumina MiSeq	2.02	39	2097	4
CBA3630	GCA_008033175.1	Kimchi	PacBio RSII	2.32	39	2266	3
Dm-9	GCA_018257095.1	Apple dumpster	Illumina NextSeq	2.18	38.8	2171	0
FDAARGOS_1003	GCA_016127255.1	Cane juice	PacBio RS; Illumina HiSeq 4000	2.11	39	2062	0
FDAARGOS_1004	GCA_016127035.1	Commercial starter culture	PacBio RS; Illumina HiSeq 4000	2.1	39.1	2114	7
HPK01	GCA_002092075.1	Dairy starter	Illumina MiSeq	1.95	39.1	1940	1
IM1374	GCA_925279165.1	Fresh curd cheese	Illumina MiSeq v3	2.08	38.9	2176	4
IM1427	GCA_925297955.1	Starter culture kefir ferment	Illumina MiSeq v3	1.91	39.1	1917	2
KMB_610	GCA_003346375.1	Bryndza cheese	Illumina MiSeq	1.98	39	1997	0
LMG 11482	GCA_014634745.1	Cane juice	Illumina NovaSeq	2.1	38.9	2041	0
LMG 11483	GCA_014634765.1	Unknown	Illumina NovaSeq	2.02	39	2014	0
LMGCF06	GCA_002092035.1	Dairy	Illumina MiSeq	1.95	39.1	1937	1
LMGCF15	GCA_002092375.1	Dairy	Illumina MiSeq	1.92	39.1	1914	1
LMGH100	GCA_002072505.1	Dairy	Illumina MiSeq	2.06	39.1	2227	1
LMGH278	GCA_002072495.1	Dairy	Illumina MiSeq	1.94	39.1	1942	1
LMGH280	GCA_002072555.1	Dairy	Illumina MiSeq	1.98	39.1	1996	1
LMGH284	GCA_002072575.1	Dairy	Illumina MiSeq	2.07	39	2190	1
LMGH61	GCA_002072515.1	Dairy	Illumina MiSeq	2.01	39.1	2014	1
LMGH83	GCA_002072475.1	Dairy	Illumina MiSeq	2.06	39.1	2229	1
LMGH95	GCA_002072565.1	Dairy	Illumina MiSeq	1.99	39	2059	1
LMGH97	GCA_002072585.1	Dairy	Illumina MiSeq	1.99	39	2042	4
LMGTW1	GCA_002092235.1	Dairy	Illumina MiSeq	1.97	39	2015	3
LMGTW3	GCA_002092355.1	Dairy	Illumina MiSeq	1.9	39.1	1915	3
LMGTW6	GCA_002092255.1	Dairy	Illumina MiSeq	1.88	39.1	1896	3
LMGTW8	GCA_002092295.1	Dairy	Illumina MiSeq	1.98	39	2039	4
LN02	GCA_002092555.1	Dairy	Illumina MiSeq	1.91	39.1	1919	3
LN12	GCA_002092635.1	Dairy	Illumina MiSeq	1.86	39.1	1851	2
LN23	GCA_002092645.1	Dairy	Illumina MiSeq	1.99	39.1	2046	1
MGBC116435	GCA_910579915.1	Caecum	N/A	2.14	38.6	2168	0
NCDO 768	GCA_012396745.1	Cane juice	Illumina NovaSeq	2.13	38.9	2075	0
PS12	GCA_000686505.1	Undefined mesophilic cheese starter culture	Illumina MiSeq	1.93	39.1	1975	4
TMW21073	GCA_020881495.1	Food	Illumina MiSeq	2.28	38.7	2258	0
TMW21195	GCA_020858715.1	Food	Illumina MiSeq	2.08	39	2042	0
TR070	GCA_006382035.1	Sourdough	Illumina MiSeq	2.23	38.8	2244	0
UBA11295	GCA_003542875.1	Unknown	Illumina HiSeq 2000	1.81	39.2	1825	0

**Figure 2:**
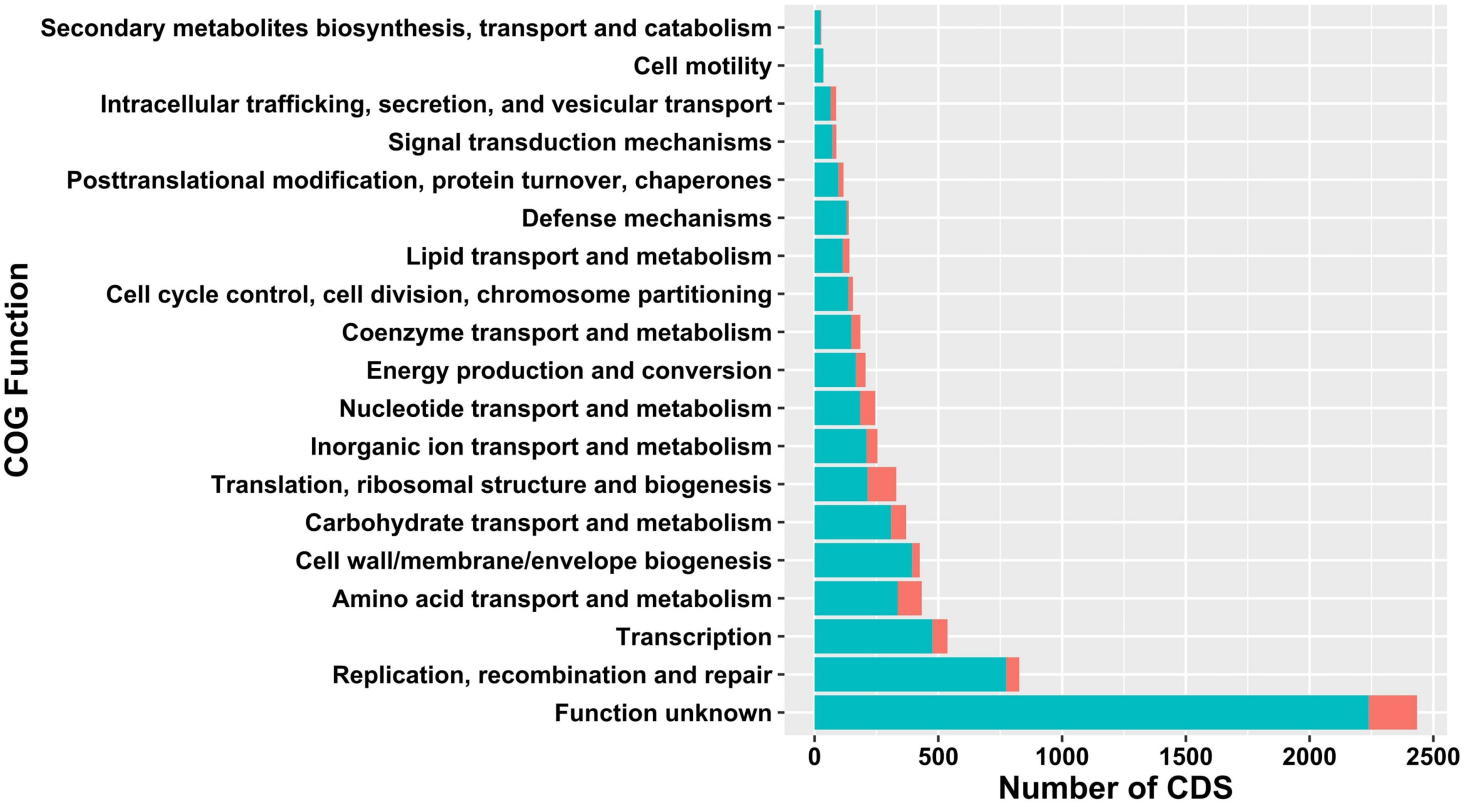
Comparison of functional COGs in pan- (light green) and core (red) genome of 38 *Ln. pseudomesenteroides* strains.

The core- and pangenomes were annotated using eggNOG-Mapper (Huerta-Cepas et al., 2017) and assigned to functional groups (Figure 2). The largest core- and pangenome category included coding sequences with functions pertained to function unknown. The second and third largest pangenome categories contained replication, recombination and repair and transcription. Pangenome categories of amino acid transport and metabolism had similar number of CDS with carbohydrate transport and metabolism. The smallest pangenome categories included cell motility and secondary metabolites biosynthesis, transport and catabolism. The second largest core genome category was composed of CDS with functions pertained to translation, ribosomal structure and biogenesis. Carbohydrate transport and metabolism, nucleotide transport and metabolism, and transcription related CDS formed the third largest core genome category. The smallest core genome categories consisted of CDS associated with cell motility and secondary metabolites biosynthesis, transport and catabolism.

**Figure 3:**
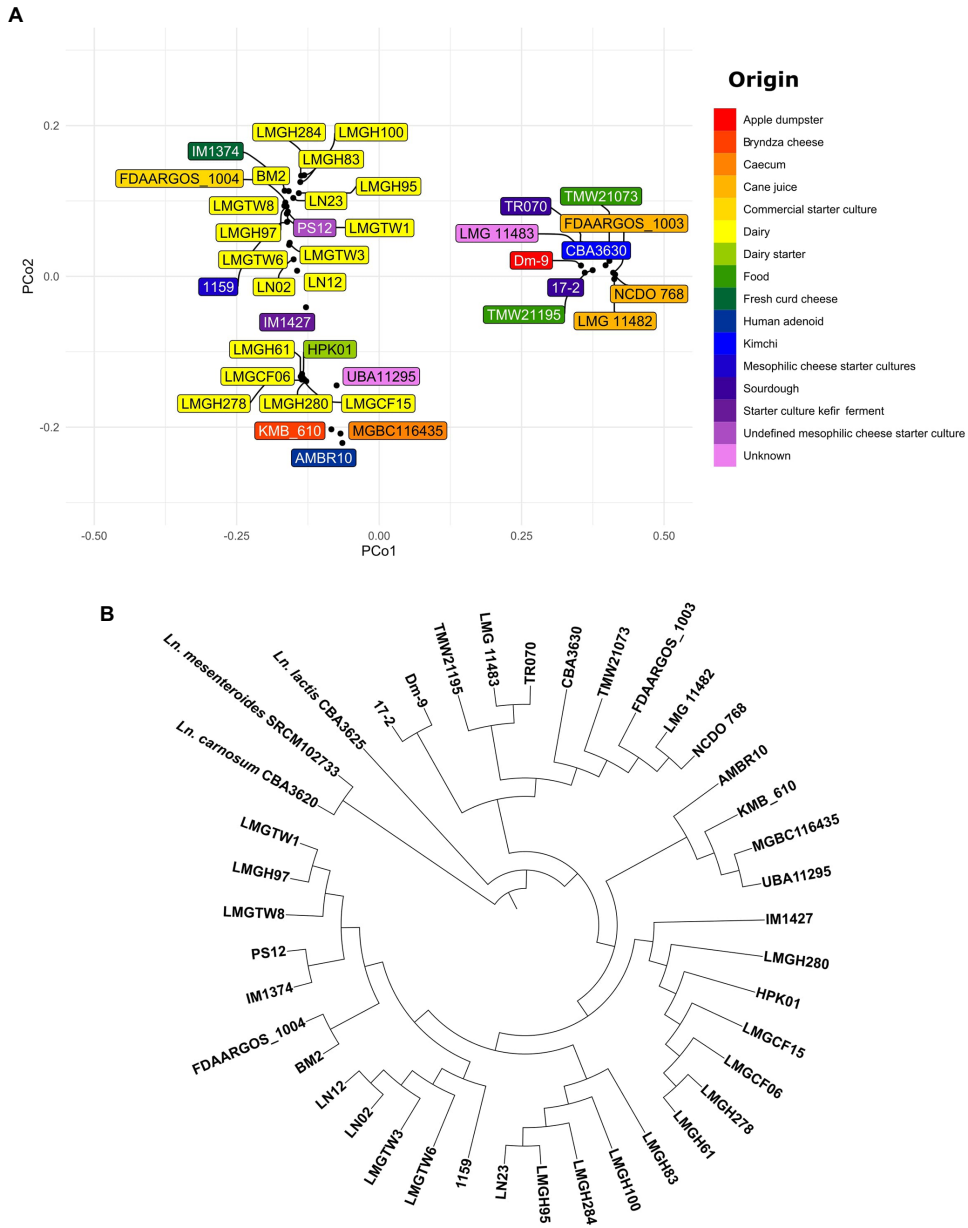
**(A)** PCoA visualization of Jaccard distances based on shared genes across 38 *Ln. pseudomesenteroides* genomes screened. The color of each box indicates a unique isolation source. **(B)** Neighbor-joining unrooted phylogenetic tree based on whole genome alignment.

To analyze the phylogenetic relationship among *Ln. pseudomesenteroides* PCoA plot and neighbor joining rooted phylogenetic tree were constructed on 38 *Ln. pseudomesenteroides* strains (Figures 3A,B). To confirm that these strains are all *Ln. pseudomesenteroides*, the outgroup strains of *Ln. lactis* CBA3625, *Ln. mesenteroides* SRCM102733, and *Ln. carnosum* CBA3620 were included in phylogenomic analysis. Dairy strains were clustered into two groups on the negative side of PCo1. On the other hand, 13 lay at positive values of PCo2. Plant-associated strains lay only at the positive side of PCo1 and PCo2 except LMG 11482, which was located on the positive side of PCo1 and negative side of PCo2 (Figure 3A). Phylogenomic analysis revealed that there are seven major branches within *Ln. pseudomesenteroides* strains and outgroup strains form two separate clades (Figure 3B). The first four branches close to each other were composed of dairy-associated strains. The fifth branch consisted of one clinical isolate, caecum, Bryndza cheese, and unknown source strains. The last two clades were far from the first five and mainly consisted of plant-based systems such as sourdough, kimchi, cane juice, and apple dumpster.

### 3.2. Carbohydrate active enzymes

Identification of carbohydrate-active enzymes (CAZyme) revealed that glycosyltransferase and glycoside hydrolase family enzymes were the most prevalent CAZymes across all *Ln. pseudomesenteroides* genomes analyzed. 17–2, TMW21073, and CBA3630 possessed the highest number of GH family CAZymes (Supplementary Figure S1). The abundance of GT family CAZymes was higher in TMW21073, NCDO 768, LMG 11482, and the type strain FDAARGOS_1003. The concentration of CE, AA, and CBM family CAZymes was remarkably lower than GH and GT family CAZymes across 38 genomes (Supplementary Figure S1). Five genomes were not predicted to encode AAs. Four main clades were identified based on the abundance of CAZymes in each genome. The first clade from the bottom-up contained sourdough, food and kimchi associated strains. However, the second clade members were pertained to plant-associated, caecum, and human clinical isolate. The third and fourth clade members were primarily belonged to dairy.

**Figure 4:**
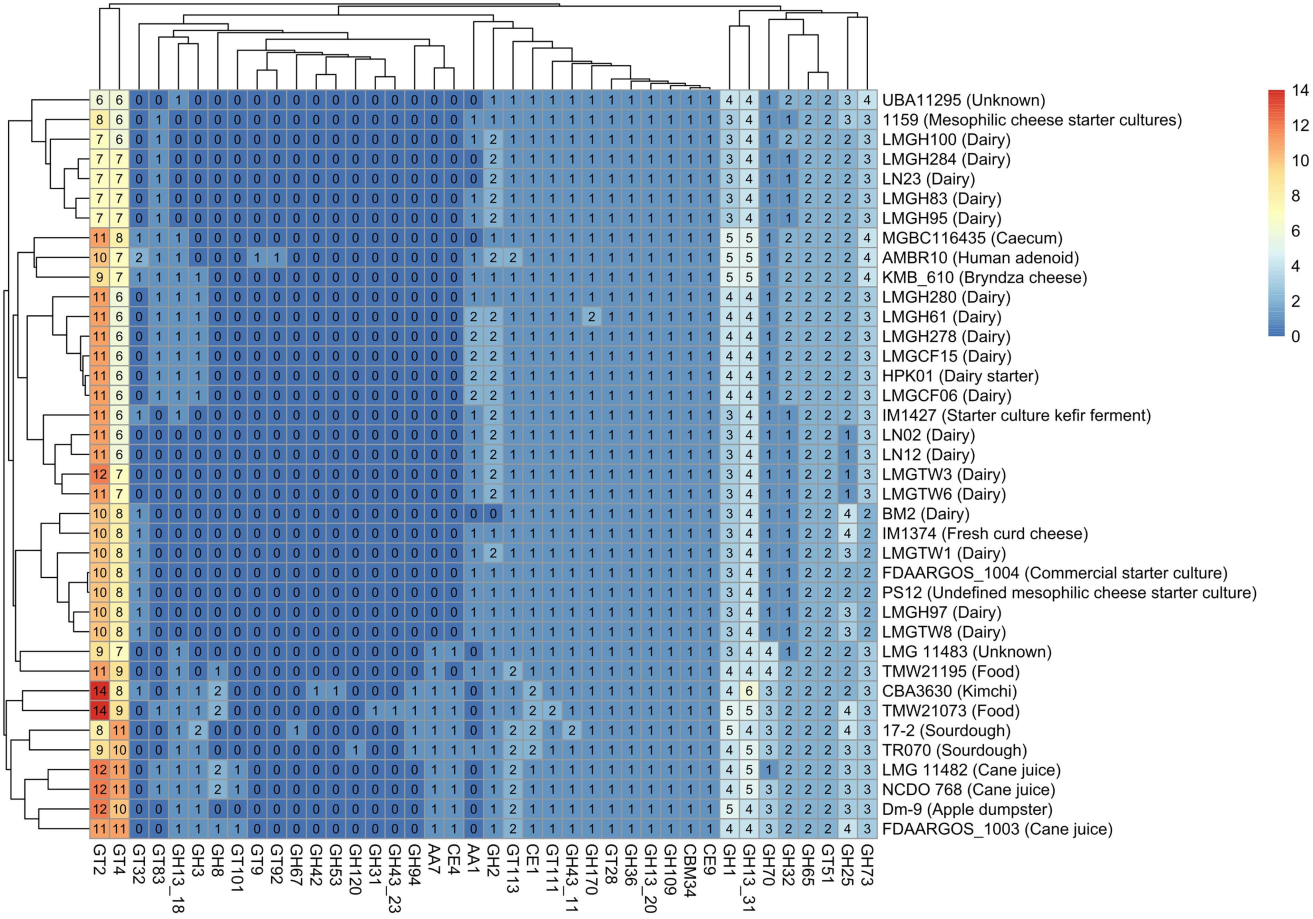
Heatmap of CAZymes distribution and clustering across 38 *Ln. pseudomesenteroides* genomes. The color gradient from lighter to darker colors represent the abundance of CAZymes found in each genome. GH: Glycoside hydrolase, GT: Glycosyltransferase, CE: Carbohydrate esterase, AA: Auxiliary activity, CBM: Carbohydrate binding module. R programming language (version 4.1.1; R Core Team, 2021) was used to draw the heatmap.

Twenty-one glycosyl hydrolases, key enzymes for the metabolism of carbohydrates, were identified among the 38 strains tested. Eleven of these existed in all strains, including GH1, GH109, GH13, GH170, GH25, GH32, GH36, GH43, GH65, GH70, and GH73. Some glycosyl hydrolases such as GH120, GH31, GH42, GH53, and GH67 were unique to TR070, TMW21073, CBA3630, CBA3630, and 17–2, respectively. While GH94 was only present in 17–2, TR070, CBA3630, and TMW21073, GH8 was present in those strains (except for 17-2) plus FDAARGOS_1003, LMG11482, NCDO 768, and TMW21195. GH2 was found in all genomes except BM2. Among 11 glycosyl transferases identified (GT101, GT111, GT113, GT2, GT28, GT32, GT4, GT51, GT83, GT9, and GT92), GT2 and GT4 composed 40% and 30% of all GT associated genes, respectively (Figure 4). GT51 was accounted for 8% of GTs whereas GT111, GT113, and GT28 represented 13.6% of GTs. The remaining five GTs were accounted for less than 5% of all GT related genes. Even though GT111, GT113, GT2, GT28, GT4, and GT51 were encoded in all strains, GT32 and GT83 were only present in 32 and 47% of the strains, respectively. GT101 was only found in FDAARGOS_1003, LMG 11482, and NCDO 768. Likewise, GT9 and GT92 were unique to human clinical isolate of AMBR10. All the strains carried CBM, CE, and AA with the exception of five genomes that lacked AAs.

### 3.3. Carbohydrate metabolism

Putative carbohydrate metabolism, citrate metabolism, malate metabolism, and mannitol metabolism genes of 38 *Ln. pseudomesenteroides* strains were detected based on the presence or absence of key genes annotated by Prokka (Table 2). Thirty-eight *Ln. pseudomesenteroides* genomes were found to be encoding phosphoketolase and fructose bisphosphate aldolase but lacked phosphofructokinase. Comparative analysis of carbohydrate metabolism-associated genes showed pronounced differences among certain strains of *Ln. pseudomesenteroides*. 76% of *Ln. pseudomesenteroides* genomes analyzed carried fructokinase functional in fructose to fructose 6-phosphate conversion. All *Ln. pseudomesenteroides* studied in the present work harbored beta-galactosidase. Two different beta-galactosidases, *lacLM* and *lacZ*, were found. While all the genomes encoded *lacLM*, only TMW21195 was predicted to encode *lacZ* that was severely truncated. *lacS*, lactose specific transporter, was carried by all *Ln. pseudomesenteroides* genomes analyzed. Genes encoding for *malL* (sucrose-isomaltose) and *malP* (maltose phosphorylase) were found in all *Ln. pseudomesenteroides* genomes. However, *malR* (HTH-type transcriptional regulator) was found in 47% of the strains that were belong to dairy. Sucrose 6-phosphate hydrolase (*scrB*) was found in 17 genomes analyzed in which 76% was belong to non-dairy associated niches (i.e., sourdough, cane juice, food, caecum, and unknown). *bglA* gene encoding beta-glucosidase was found in all *Ln. pseudomesenteroides* isolates except AMBR10. *trePP* encoding for trehalose 6-phosphate phosphorylase was available across all strains. *treA* (trehalose-6-phosphate hydrolase) was absent in all genomes except for Bryndza cheese isolate of KMB_610 although the gene was truncated. *xylA*, *xylB*, and *xylG* encoding xylulose isomerase, xylulose kinase, and xylulose transport protein, respectively, were found in all *Ln. pseudomesenteroides* tested. *araBAD* operon encoding for arabinose metabolism pathway was only available in plant-associated strains of CBA3630, Dm-9, FDAARGOS_1003, LMG 11482, LMG 11483, NCDO 768, TMW21073, and TR070.

**Table 2:** Presence (+) and absence (−) of putative carbohydrate metabolism, citrate metabolism, malate metabolism, and mannitol metabolism of 38 *Ln. pseudomesenteroides*.

Strain	araA	araB	araD	citC	citD	citE	citF	citG	citO	citS	fba	fruA	galE	galK	galT	lacL	lacM	lacS	lacZ	malF	malG	malL	malP	malR	malX	manA	manX	manZ	scrB	treA	trePP	xylA	xylB	xylG	treA	trePP	bglA	fruA	levE	scrB	mdh	mdh1	gtfC	gmue	fumC	mleA	aspC	alsS	aldC
1159	−	−	−	+	+	+	+	+	+	+	+	−	+	+	+	+	+	+	−	−	−	+	+	+	−	−	+	+	−	−	+	+	+	+	−	+	+	−	−	−	+	−	#	+	−	+	+	+	+
17–2	−	−	−	+	+	+	+	+	+	#	+	−	+	+	+	+	+	+	−	−	−	+	+	−	+	+	+	+	+	−	+	+	+	+	−	+	+	−	+	+	+	+	+	+	+	+	+	+	+
AMBR10	−	−	−	+	+	+	+	+	+	+	+	+	+	+	+	+	+	+	−	−	−	+	+	−	+	−	+	+	+	−	+	+	+	+	−	+	−	+	+	+	+	−	#	+	+	+	+	+	+
BM2	−	−	−	+	+	+	+	+	+	+	+	−	−	+	+	#	+	+	−	−	−	+	+	+	−	−	+	+	−	−	+	+	+	+	−	+	+	−	−	−	+	−	#	+	−	+	+	+	+
CBA3630	+	+	+	+	+	+	+	+	#	+	+	−	+	+	+	+	+	+	−	−	−	+	+	−	+	+	+	+	+	−	+	+	+	+	−	+	+	−	+	+	+	−	+	+	+	+	+	+	+
Dm-9	+	+	+	+	+	+	+	+	+	#	+	−	+	+	+	+	+	+	−	−	−	+	+	−	+	+	+	+	+	−	+	+	+	+	−	+	+	−	+	+	+	+	#	+	+	+	+	+	+
FDAARGOS_1003	+	+	+	−	−	−	−	−	−	#	+	−	+	+	+	+	+	+	−	−	−	+	+	−	+	+	+	+	+	−	+	+	+	+	−	+	+	−	−	+	+	+	+	+	−	−	+	+	+
FDAARGOS_1004	−	−	−	+	+	+	+	+	+	+	+	−	+	+	+	+	+	+	−	−	−	+	+	+	−	−	+	+	−	−	+	+	+	+	−	+	+	−	−	−	+	−	#	+	−	+	+	+	+
HPK01	−	−	−	+	+	+	+	+	+	+	+	−	#	+	+	+	+	+	−	−	−	+	+	−	−	−	+	+	+	−	+	+	+	+	−	+	+	−	+	+	+	−	+	+	−	+	+	+	+
IM1374	−	−	−	+	+	+	+	+	+	+	+	−	+	+	+	+	+	+	−	−	−	+	+	+	−	−	+	+	−	−	+	+	+	+	−	+	+	−	−	−	+	−	#	+	−	+	+	+	+
IM1427	−	−	−	+	+	+	+	+	+	+	+	−	+	+	+	+	+	+	−	−	−	+	+	−	+	−	+	+	−	−	+	+	+	+	−	+	+	−	+	−	+	−	+	+	−	−	+	+	+
KMB_610	−	−	−	+	+	+	+	+	+	+	+	−	+	+	+	+	+	+	−	−	−	+	+	−	−	−	+	+	+	+	+	+	+	+	#	+	+	−	+	+	+	−	+	+	+	+	+	+	+
LMG 11482	+	+	+	−	−	−	−	−	−	#	+	−	#	+	+	+	+	+	−	−	−	+	+	−	+	+	+	+	+	−	+	+	+	+	−	+	+	−	−	+	+	+	+	+	+	+	+	+	+
LMG 11483	+	+	+	−	−	−	−	−	−	+	+	−	#	+	+	+	+	+	−	−	−	+	+	−	+	+	+	+	+	−	+	+	+	+	−	+	+	−	−	+	+	−	+	+	−	−	+	+	+
LMGCF06	−	−	−	+	+	+	+	+	+	+	+	−	#	+	+	+	+	+	−	−	−	+	+	−	−	−	+	+	+	−	+	+	+	+	−	+	+	−	+	+	+	−	+	+	−	+	+	+	+
LMGCF15	−	−	−	+	+	+	+	+	+	+	+	−	+	+	+	+	+	+	−	−	−	+	+	−	−	−	+	+	+	−	+	+	+	+	−	+	+	−	+	+	+	−	+	+	−	+	+	+	+
LMGH100	−	−	−	+	+	+	+	+	+	+	+	−	+	+	+	+	+	+	−	−	+	+	+	+	−	−	+	+	−	−	+	+	+	+	−	+	+	−	−	−	+	−	#	−	−	+	+	+	+
LMGH278	−	−	−	+	+	+	+	+	+	+	+	−	−	+	+	+	+	+	−	−	−	+	+	−	−	−	+	+	+	−	+	+	+	+	−	+	+	−	+	+	+	−	+	+	−	+	+	+	+
LMGH280	−	−	−	+	+	+	+	+	+	+	+	−	+	+	+	+	+	+	−	+	−	+	+	−	−	−	+	+	+	−	+	+	+	+	−	+	+	−	+	+	+	−	+	+	−	+	+	+	+
LMGH284	−	−	−	+	+	+	+	+	#	+	+	−	+	+	+	+	+	+	−	−	−	+	+	+	−	−	+	+	−	−	+	+	+	+	−	+	+	−	−	−	+	−	#	−	−	+	+	+	+
LMGH61	−	−	−	+	+	+	+	+	+	+	+	−	+	+	+	+	+	+	−	−	−	+	+	−	−	−	+	+	+	−	+	+	+	+	−	+	+	−	+	+	+	−	+	+	−	+	+	+	+
LMGH83	−	−	−	+	+	+	+	+	#	+	+	−	+	+	+	+	+	+	−	−	−	+	+	+	−	−	+	+	−	−	+	+	+	+	−	+	+	−	−	−	+	−	#	−	−	+	+	+	+
LMGH95	−	−	−	+	+	+	+	+	+	+	+	−	+	+	+	+	+	+	−	−	−	+	+	+	−	−	+	+	−	−	+	+	+	+	−	+	+	−	−	−	+	−	#	−	−	+	+	+	+
LMGH97	−	−	−	+	+	+	+	+	+	+	+	−	#	+	+	+	+	+	−	−	−	+	+	+	−	−	+	+	−	−	+	+	+	+	−	+	+	−	−	−	#	−	#	+	−	+	+	+	+
LMGTW1	−	−	−	+	+	+	+	+	+	+	+	−	#	+	+	+	+	+	−	−	−	+	+	+	−	−	+	+	−	−	+	+	+	+	−	+	+	−	−	−	+	−	#	+	−	+	+	+	+
LMGTW3	−	−	−	+	+	+	+	+	+	+	+	−	#	+	+	+	+	+	−	−	#	+	+	+	−	−	+	+	−	−	+	+	+	+	−	+	+	−	−	−	#	−	#	+	−	−	+	+	+
LMGTW6	−	−	−	+	+	+	+	+	#	+	+	−	−	+	+	+	+	+	−	−	#	+	+	+	−	−	+	+	−	−	+	+	+	+	−	+	+	−	−	−	#	−	#	+	−	−	+	+	+
LMGTW8	−	−	−	+	+	+	+	+	+	+	+	−	+	+	+	+	+	+	−	−	−	+	+	+	−	−	+	+	−	−	+	+	+	+	−	+	+	−	−	−	+	−	#	−	−	+	+	+	+
LN02	−	−	−	+	+	+	+	+	+	+	+	−	#	+	+	+	+	+	−	−	#	+	+	+	−	−	+	+	−	−	+	+	+	+	−	+	+	−	−	−	#	−	#	+	−	−	+	+	+
LN12	−	−	−	+	+	+	+	+	+	+	+	−	−	+	+	+	+	+	−	−	#	+	+	+	−	−	+	+	−	−	+	+	+	+	−	+	+	−	−	−	#	−	#	−	−	−	+	+	+
LN23	−	−	−	+	+	+	+	+	+	+	+	−	+	+	+	+	+	+	−	−	−	+	+	+	−	−	+	+	−	−	+	+	+	+	−	+	+	−	−	−	+	−	#	−	−	+	+	+	+
MGBC116435	−	−	−	+	+	+	+	+	+	+	+	−	#	+	+	+	+	+	−	−	−	+	+	−	+	−	+	+	+	−	+	+	+	+	−	+	+	−	+	+	+	−	+	+	+	+	+	+	+
NCDO 768	+	+	+	−	−	−	−	−	−	#	+	−	#	+	+	+	+	+	−	−	−	+	+	−	+	+	+	+	+	−	+	+	+	+	−	+	+	−	+	+	+	+	+	−	+	+	+	+	+
PS12	−	−	−	+	+	+	+	+	+	+	+	−	+	+	+	+	+	+	−	−	−	+	+	+	−	−	+	+	−	−	+	+	+	+	−	+	+	−	−	−	+	−	#	+	−	+	+	+	+
TMW21073	+	+	+	+	+	+	+	+	+	+	#	−	+	+	+	+	+	+	−	−	−	+	+	−	+	+	+	+	+	−	+	+	+	+	−	+	+	−	+	+	+	+	+	+	+	+	+	+	+
TMW21195	−	−	−	+	+	+	+	+	+	#	#	−	+	+	+	+	+	+	#	−	−	+	+	−	+	+	+	+	+	−	+	+	+	+	−	+	+	−	+	+	+	+	+	+	−	−	+	+	+
TR070	+	+	+	−	−	−	−	−	−	+	+	−	+	+	+	+	+	+	−	−	−	+	+	#	−	+	+	+	+	−	+	+	+	+	−	+	+	−	+	+	+	+	+	+	−	−	+	+	+
UBA11295	−	−	−	#	+	+	+	+	+	+	+	−	−	+	+	+	+	+	−	−	−	+	+	−	−	−	+	+	+	−	+	+	+	+	−	+	+	−	+	+	+	−	+	−	+	+	+	+	+

# means present but truncated gene. Green: Gene present; Pink: Gene absent; Yellow: Truncated gene.

Thirty-eight *Ln. pseudomesenteroides* strains were predicted to carry key enzymes functional in galactose utilization through the Leloir pathway except for BM2, LMGH278, LMGTW6, LN12, and UBA11295, which missed *galE* encoding UDP-glucose 4-epimerase. The maltose operon genes *maLF*, *malG*, *malL*, *malP*, and *malR* presence did not show a homogenous distribution across 38 *Ln. pseudomesenteroides* strains. For example, *malF* was not present in any of the strains except LMGH280. Although *malG* existed in LMGH100, LMGTW3, LMGTW6, LN02, and LN12, only the former strain carried a complete gene while the latter four contained truncated gene.

Genes that were found in *citCDEFGOS* operon composed of citrate lyase ligase (*citC*), citrate lyase (*citDEF*), holo-ACP synthase (*citG*), transcriptional regulator (*citO*) and sodium dependent citrate transporter (*citS*). Citrate uptake and metabolism operon was encoded by all strains but cane juice isolates of FDAARGOS_1003, LMG 11482, NCDO 768, sourdough isolate of TR070, and unknown isolate of LMG 11483. The genes encoding for malate dehydrogenase was available in NCDO 768, LMG 11482, FDAARGOS_1003, Dm-9, 17–2, TMW21195, TR070, and TMW21073. Fumarate hydratase was found in 17–2, AMBR10, CBA3630, Dm-9, KMB_610, LMG 11482, MGBC116435, NCDO 768, TMW21073, and UBA11295. Malolactic enzyme was found in 76% of all genomes. The gene encoding for aspartate aminotransferase functional in production of aspartate from oxaloacetate was possessed by all genomes. Acetolactate synthase and acetolactate decarboxylase that are functional in pyruvate transformations into alpha-acetolactate and acetoin were also harbored by all genomes. Mannitol dehydrogenase (*mdh*) encoding for mannitol production from fructose was also evident in all genomes however *mdh* was truncated in ~13% of strains primarily belonging to dairy. All *Ln. pseudomesenteroides* genomes encoded dextransucrase which converts sucrose to dextran and fructose. However, 50% of *Ln. pseudomesenteroides* strains carried severely truncated dextransucrase gene which were primarily isolated from dairy except for Dm-9 (i.e., apple dumpster).

### 3.4. Proteolytic activity

The genes involved in proteolytic activity showed differences between strains (Table 3). The genes encoding for peptide ABC transporter operon, *oppABCDF*, were found in all genomes; however, *oppF* was truncated in 63% of the genomes. *oppB* was truncated in FDAARGOS_1003, LMG 11482, LMGH100, and NCDO 768. *oppA* and *oppC* were truncated in LMGH280 and PS12, respectively. *prtP* gene encoding for pII type serine proteinase functional against casein (Frantzen et al., 2017) was found in ~53% of *Ln. pseudomesenteroides* genomes. *Leuconostoc pseudomesenteroides* genomes also contained a range of aminotransferases and peptidases. For example*, pepN* (aminopeptidase) was carried by all genomes in which LMG 11483 and TR070 harbored truncated gene*. pepA, pepC, pepQ*, and *pepX* were also found in all genomes. 47% of the *Ln. pseudomesenteroides* genomes had the *pepV* gene encoding for beta-alanine dipeptidase. All *Ln. pseudomesenteroides* genomes had complete *pepS* and *pepT* genes with the exception of UBA11295 which carried truncated forms of those genes. Despite *pepF* was carried by all *Ln. pseudomesenteroides* strains, ~18% of them possessed truncated gene.

**Table 3:** Presence (+) and absence (−) of putative proteolytic activity of 38 *Ln. pseudomesenteroides*.

Strain	oppA	oppB	oppC	oppD	oppF	pepA	pepC	pepF	pepN	pepO	pepQ	pepS	pepT	pepV	pepX	prtP
1159	+	+	+	+	#	+	+	+	+	+	+	+	+	+	+	−
17–2	+	+	+	+	+	+	+	+	+	+	+	+	+	−	+	#
AMBR10	+	+	+	+	+	+	+	+	+	+	+	+	+	−	+	#
BM2	+	+	+	+	#	+	+	+	+	+	+	+	+	+	+	−
CBA3630	+	+	+	+	+	+	+	#	+	+	+	+	+	−	+	#
Dm-9	+	+	+	+	+	+	+	+	+	+	+	+	+	−	+	#
FDAARGOS_1003	+	#	+	+	+	+	+	#	+	+	+	+	+	−	+	#
FDAARGOS_1004	+	+	+	+	#	+	+	+	+	+	+	+	+	+	+	−
HPK01	+	+	+	+	#	+	+	+	+	+	+	+	+	+	+	+
IM1374	+	+	+	+	#	+	+	+	+	+	+	+	+	+	+	−
IM1427	+	+	+	+	#	+	+	+	+	+	+	+	+	−	+	−
KMB_610	+	+	+	+	+	+	+	+	+	+	+	+	+	−	+	#
LMG 11482	+	#	+	+	+	+	+	#	+	+	+	+	+	−	+	#
LMG 11483	+	+	+	+	+	+	+	+	#	+	+	+	+	−	+	+
LMGCF06	+	+	+	+	#	+	+	+	+	+	+	+	+	+	+	+
LMGCF15	+	+	+	+	#	+	+	+	+	+	+	+	+	+	+	#
LMGH100	+	#	+	+	#	+	+	+	+	+	+	+	+	+	+	−
LMGH278	+	+	+	+	#	+	+	+	+	+	+	+	+	+	+	#
LMGH280	#	+	+	+	#	+	+	+	+	+	+	+	+	+	+	+
LMGH284	+	+	+	+	#	+	+	#	+	+	+	+	+	+	+	−
LMGH61	+	+	+	+	#	+	+	+	+	+	+	+	+	+	+	+
LMGH83	+	+	+	+	#	+	+	+	+	+	+	+	+	−	+	−
LMGH95	+	+	+	+	#	+	+	+	+	+	+	+	+	+	+	−
LMGH97	+	+	+	+	#	+	+	+	+	+	+	+	+	#	+	−
LMGTW1	+	+	+	+	#	+	+	+	+	+	+	+	+	+	+	−
LMGTW3	+	+	+	+	#	+	+	+	+	+	+	+	+	+	+	−
LMGTW6	+	+	+	+	#	+	+	+	+	+	+	+	+	+	+	−
LMGTW8	+	+	+	+	#	+	+	+	+	+	+	+	+	+	+	−
LN02	+	+	+	+	#	+	+	+	+	+	+	+	+	+	+	−
LN12	+	+	+	+	#	+	+	+	+	+	+	+	+	+	+	−
LN23	+	+	+	+	#	+	+	+	+	#	+	+	+	+	+	−
MGBC116435	+	+	+	+	+	+	+	+	+	+	+	+	+	−	+	#
NCDO 768	+	#	+	+	+	+	+	#	+	+	+	+	+	−	+	#
PS12	+	+	#	+	#	+	+	+	+	+	+	+	+	+	+	−
TMW21073	+	+	+	+	+	+	+	#	+	+	+	+	+	−	+	#
TMW21195	+	+	+	+	+	+	+	+	+	+	+	+	+	−	+	#
TR070	+	+	+	+	+	+	+	#	#	+	+	+	+	−	+	#
UBA11295	+	+	+	+	+	+	+	+	+	+	+	#	#	−	+	#

# means present but truncated gene. Green: Gene present; Pink: Gene absent; Yellow: Truncated gene.

**Figure 5:**
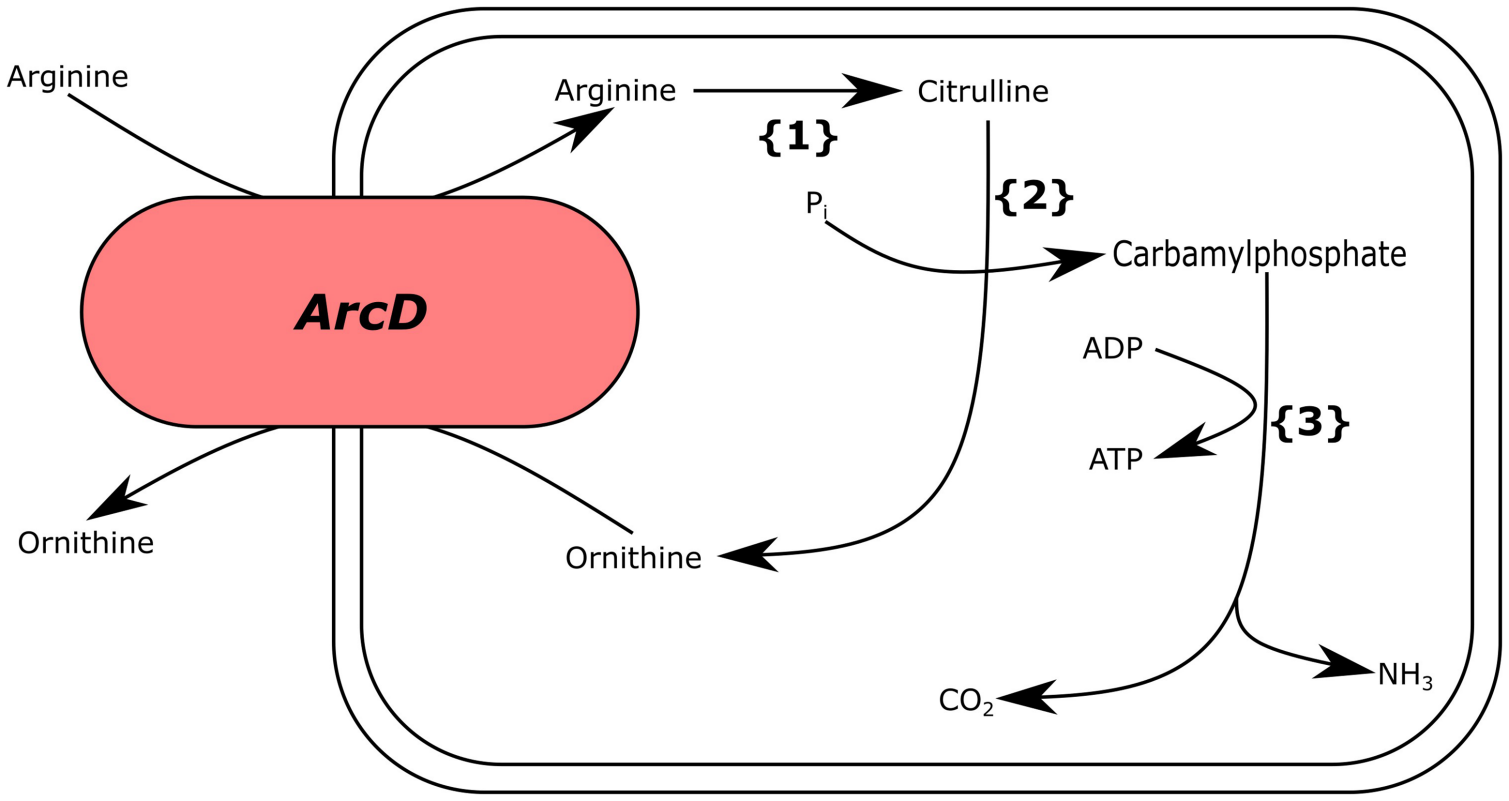
Putative arginine deiminase pathway for *Ln. pseudomesenteroides* 17–2 and TMW21195. {1} arginine deiminase, {2} ornithine transcarbamoylase, {3} carbamate kinase.

We also analyzed *Ln. pseudomesenteroides* genomes for genes encoding for arginine deiminase (ADI) metabolism (*arcA*, *arcB*, *arcC*, and *arcD*) and found that only two genomes (i.e., 17–2 and TMW21195) carried the complete gene set required for ADI metabolism (Figure 5).

### 3.5. Mobile genetic elements

All 38 genomes of *Ln. pseudomesenteroides* were explored for the existence of mobile genetic elements of plasmids, transposable elements, prophage, and CRISPR locus (Table 4). A total of 62 plasmids were identified in ~68% of *Ln. pseudomesenteroides* genomes analyzed (Table 1, Supplementary Table S2). Ten unique plasmids were detected. Transposable elements were explored using RepeatMasker (Smit and Rubley, 2008) tool, and these elements accounted for less than 0.1% of each *Ln. pseudomesenteroides* genomes. CBA3630, FDAARGOS_1003, and FDAARGOS_1004 genomes were predicted to harbor the largest number of (i.e., 145, 140, and 141) transposable elements. By grouping the transposable elements in 38 *Ln. pseudomesenteroides* strains, simple repeats accounted for the largest proportion, followed by tRNA, A-rich low complexity, rRNA, LINEs, SINEs, and DNA transposons. The IS elements were also screened in 38 *Ln. pseudomesenteroides* genomes using ISfinder (Siguier et al., 2006). AMBR10, LMGTW3, LMGTW6, and LN02 carried the highest number of IS elements. By classifying the IS elements in 38 genomes, IS6 was the most abundant element, followed by IS30, ISLre2, and IS3. IS1, IS256, IS66, ISL3, IS982, IS4, Tn3, IS21, and IS110 accounted for only ~9% of total IS elements found in 38 *Ln. pseudomesenteroides* (Supplementary Table S3).

**Table 4:** Putative mobile genetic elements of *Ln. pseudomesenteroides.*

Strain	Transposable elements	IS elements	Prophage	CRISPR
1159	114	15	6 (1 intact)	1
17–2	127	2	3 (1 intact)	0
AMBR10	133	58	8 (1 intact)	1
BM2	110	13	9 (1 intact)	1
CBA3630	145	4	4 (1 intact)	0
Dm-9	108	3	1 (0 intact)	0
FDAARGOS_1003	140	1	3 (2 intact)	0
FDAARGOS_1004	141	15	11 (0 intact)	1
HPK01	101	6	4 (0 intact)	1
IM1374	116	15	8 (2 intact)	1
IM1427	110	7	7 (1 intact)	1
KMB_610	99	7	3 (0 intact)	0
LMG 11482	107	1	1 (1 intact)	0
LMG 11483	106	3	2 (0 intact)	0
LMGCF06	101	6	3 (0 intact)	1
LMGCF15	100	6	2 (0 intact)	1
LMGH100	104	31	6 (0 intact)	1
LMGH278	101	6	3 (0 intact)	1
LMGH280	109	7	6 (1 intact)	1
LMGH284	110	15	7 (0 intact)	1
LMGH61	118	7	6 (0 intact)	1
LMGH83	106	31	8 (0 intact)	0
LMGH95	111	31	4 (0 intact)	1
LMGH97	109	15	4 (1 intact)	1
LMGTW1	104	6	7 (0 intact)	1
LMGTW3	104	42	3 (0 intact)	1
LMGTW6	100	42	5 (0 intact)	1
LMGTW8	106	15	9 (0 intact)	1
LN02	107	42	7 (0 intact)	1
LN12	104	33	4 (0 intact)	1
LN23	102	15	6 (0 intact)	1
MGBC116435	109	34	6 (1 intact)	1
NCDO 768	109	1	1 (1 intact)	0
PS12	97	15	6 (0 intact)	1
TMW21073	113	5	3 (2 intact)	0
TMW21195	104	7	2 (0 intact)	0
TR070	107	4	8 (1 intact)	0
UBA11295	73	2	3 (2 intact)	1

The analysis of prophages *via* PHASTER (Arndt et al., 2016) identified 189 prophage-like elements, of which 20 were intact, 123 were incomplete, and 46 were questionable. Out of 38 *Ln. pseudomesenteroides* screened, only 16 were predicted to encode intact prophages, while all strains carried incomplete and/or questionable prophages. By classifying the prophage-like elements, it was found that PHAGE_Lactob_phiAT3_NC_00589 accounted for the largest portion, followed by PHAGE_Lactoc_bIL309_NC_00266, PHAGE_Lactob_Lb_NC_04798, and PHAGE_Lactob_T25_NC_04862.

Thirty-eight *Ln. pseudomesenteroides* strains were also evaluated for CRISPR locus using CRISPRviz and CRISPRCasFinder tools. Because CRISPR varied in evidence level when using the CRISPRCasFinder tool, we considered the ones that exceeded evidence level one (Couvin et al., 2018). A total of 28 strains included complete CRISPR-Cas systems which belonged to Type IIA according to CRISPRCasFinder results.

**Figure 6:**
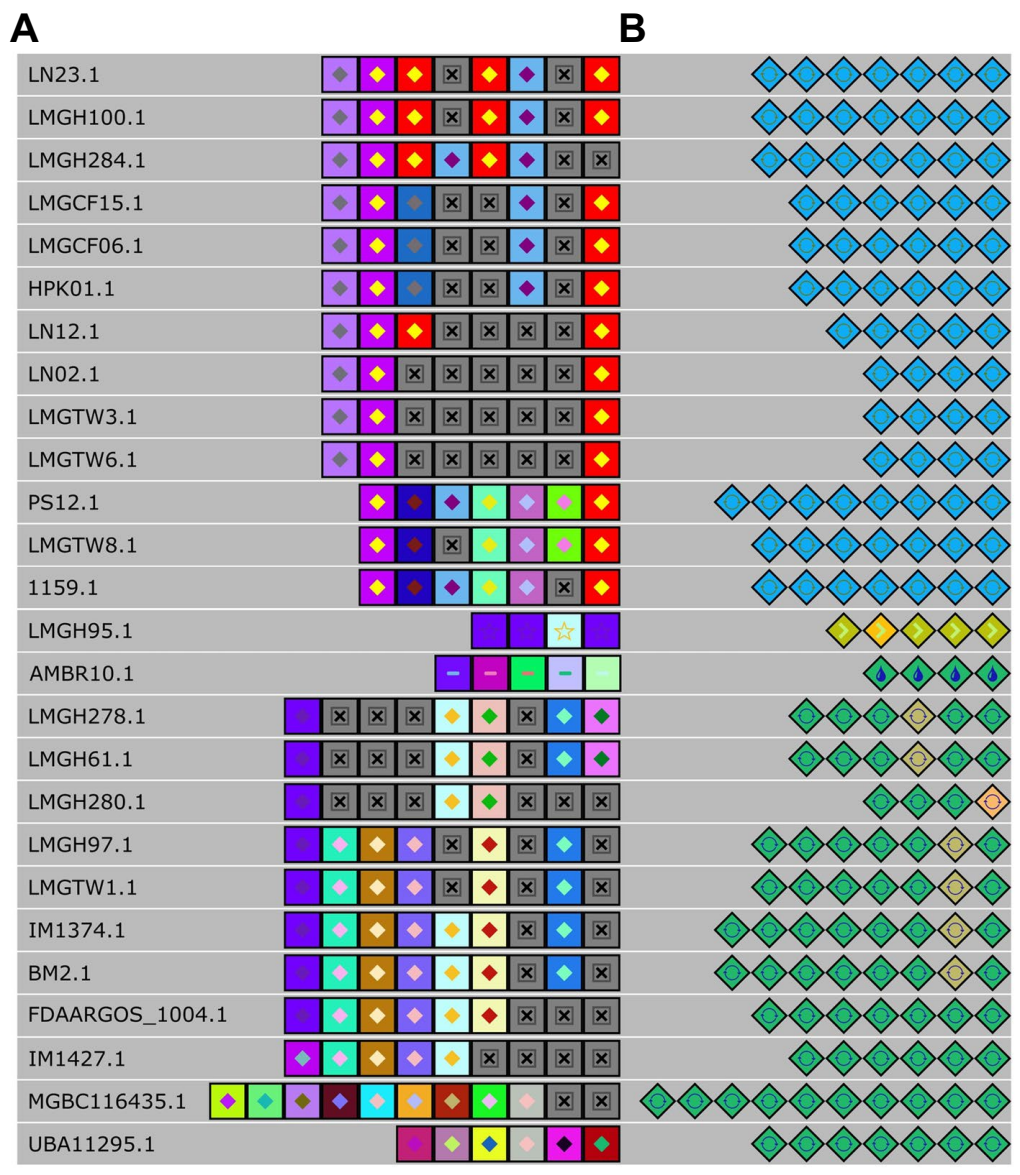
Alignment of spacers **(A)** and repeats **(B)** of each detected CRISPR locus. Each colored diamond represents a unique repeat, and each colored square represents a unique spacer in the CRISPR-Cas system. Grey “x” boxes show missing spacer.

To further elaborate our understanding of the CRISPR-Cas system in *Ln. pseudomesenteroides*, we identified and located repeats and spacers and successfully assigned them to canonical types and subtypes (Figure 6A). Six distinct groups of spacers were predicted to have 100% identity in their corresponding groups. The first group from the top down consisted of LN23 and LMGH100; the second group was composed of LMGCF15, LMGCF06, and HPK01; the third group was comprised of LN02, LMGTW3, and LMGTW6; the fourth group members were LMGH278, and LMGH61; the fifth group contained LMGH97 and LMGTW1; and the last group had IM1374 and BM2. The following strains of LMGH95, AMBR10, MGBC116435, and UBA11295 were not aligned into a group due to their diverse spacer compositions. LN12, LMGH280, and FDAARGOS_1004 did not participate in a group due to a single mismatch of spacer content. In parallel, LMGH284, and IM1427 were not laid into six groups owing to double mismatches. Nevertheless, LMGH95 and AMBR10 had no spacer identity with any CRISPR-Cas containing *Ln. pseudomesenteroides* genomes. By grouping according to repeat identity across 28 *Ln. pseudomesenteroides* strains, eight groups emerged (Figure 6B). LMGH95 and AMBR10 did not show any repeat identity with any of the genomes shown in Figure 6B. LMGH280 did not participate in a group because of a double mismatch in its repeats.

To determine horizontally transferred genes across 38 *Ln. pseudomesenteroides* strains COLOMBO software (version 4.0) was used. All genomes contained varying ratios of HGT except LN23 and UBA11295, which did not predict to carry alien genes (Supplementary Table S4). When grouped by HGT donors, *Bacillus akibai* JCM 9157 accounted for the largest proportion, followed by *Psychroserpens burtonensis* DSM 12212 and *Flavobacterium frigoris* PS1. AMBR10 was predicted to acquire the highest portion of alien genes (i.e., nine genes) followed by 17–2, Dm-9, FDAARGOS_1004, IM1374, 1159, MGBC116435, and TR070, respectively.

### 3.6. Secondary metabolites

The 38 *Ln. pseudomesenteroides* genomes were screened for the existence of gene clusters encoding for secondary metabolites using antiSMASH (Blin et al., 2021). A total of 10 secondary metabolites found were classified as: Alkoloid (62), NRP (4), NRP-polyketide (38), Nucleoside (1), Phenazine (2), other (Shikimate-derived) (7), Polyketide (26), RiPP (97), Saccharide (77) and Terpene (61) Supplementary Table S5). All *Ln. pseudomesenteroides* genomes analyzed were predicted to encode secondary metabolites producing gene clusters. Ten *Ln. pseudomesenteroides* strains were predicted to carry putative bacteriocin gene clusters namely acidocin B, gassericin A, gassericin E, gassericin T, glycocin F, plantaricyclin, lactocin S, salivaricin CRL1328 α peptide, salivaricin CRL1328 β peptide, and lacticin 481 (Supplementary Table S5). However, the similarity scores achieved for all secondary metabolites including bacteriocins were in the range of 0.07 and 0.3 which reveal that the likelihood of presence of putative secondary metabolite gene clusters in *Ln. pseudomesenteroides* is low. Hence, *Ln. pseudomesenteroides* can be considered as low potential producer of secondary metabolites. This is also supported by Figure 2 results that number of COG functions associated with secondary metabolites biosynthesis had the lowest number of CDS in pangenome of 38 *Ln. pseudomesenteroides*. Bacteriocin screening of 38 strains were also performed using BAGEL4 which predicted three kinds of bacteriocins (Supplementary Table S1), of which two of them were undefined and carried by AMBR10 and CBA3630. Garvicin Q family class II bacteriocin was predicted to be encoded by MGBC116435 only.

## 4. Discussion

The present study aimed to explore the genetic diversity and biotechnological potential of *Ln. pseudomesenteroides* strains isolated from diverse ecological niches such as dairy, sourdough, kimchi, apple dumpster, cane juice, caecum, and human adenoid through comparative genomics. The average genome size of 38 *Ln. pseudomesenteroides* was 2.04 Mb (ranging from 1.81 to 2.32 Mb) which is in the range with lactic acid bacteria in general. In addition, average G + C content was found at 39% (ranging from 38.5% to 39.2%) in consensus with low G + C LAB implying *Ln. pseudomesenteroides* had gone through genetic drift (Makarova et al., 2006; Brandt and Barrangou, 2018). All 38 strains shared a mere 12% of COGs in the core genome, revealing the genotypic differences in *Ln. pseudomesenteroides* were primarily determined by the accessory genome. In addition, ~21.4% of the core genome is composed of sequences without a known function producing future candidates for functional studies (Nethery et al., 2019). Pangenome analysis of *Ln. pseudomesenteroides* revealed an open genome, resulting in this species’ functional diversity (Li et al., 2021). It is generally accepted that broadly distributed bacterial species often carry open pangenomes, which leads to the acquisition of alien genes from the environmental niche and adjust against environmental conditions such as *E. coli* (Fu and Qin, 2012), *Bacillus cereus* (Bazinet, 2017), and *Streptococcus pneumonia* (Tettelin et al., 2008). The variable genes found in *Ln. pseudomesenteroides via* Roary was ~88% of the total gene content in the pangenome, which proposes a large degree of diversity within this species (Medini et al., 2005).

The distribution of genomes in the PCoA plot and neighbor-joining rooted phylogenetic tree demonstrate that strains of the same origin are usually clustered together or not too distant. For example, all dairy-associated strains were located on the left-hand side of the PCoA plot as two clusters (Figure 3A). On the other hand, plant-associated isolates from cane juice (FDAARGOS_1003, LMG 11482, and NCDO 768), sourdough (17–2 and TR070), kimchi (CBA3630), apple dumpster (Dm-9) positioned on the right-hand side of the plot as a group. In parallel, the phylogenetic association of dairy or plant-originated strains showed a similar clade formation in the phylogenetic tree (Figure 3B). This perhaps implies that *Ln. pseudomesenteroides* had experienced evolutionary adaptation to their corresponding microniche. We would expect that phylogenetically related strains would share similar isolation origins (Brandt et al., 2020). Outliers to this would be human adenoid and caecum isolates of AMBR10 and MGBC116435, respectively, which lay together with dairy isolates at the negative side of the PCo2. Nevertheless, those two strains formed a separate clade with KMB_610 and UBA11295. The discrepancy of location of AMBR10 in PCoA plot might be due to harboring a single plasmid in its genome. CAZyme distribution across 38 *Ln. pseudomesenteroides* genomes revealed four distinct clades, with plant-derived strains, caecum, and human adenoid isolates forming the first two closely related clades. Dairy-associated isolates and two unknown sourced genomes comprised the third and fourth clades. This finding supports the phenomenon that isolation source is an important factor causing genomic diversity in the carbohydrate metabolism of *Ln. pseudomesenteroides*.

*In silico* prediction of GHs encoded in 38 *Ln. pseudomesenteroides* genomes revealed pangenome is composed of genes encoding GHs that belong to 21 different GH families involved in carbohydrate metabolism (Figure 4). Members of GH13 family represented the largest proportion of all GHs predicted in 38 *Ln. pseudomesenteroides* genomes accounting for 18%, which is consistent with the previous report for genus *Leuconostoc* (Sharma et al., 2022). GH13 is belong to alpha amylase group and possesses catalytic machinery and conserved sequence regions (Martinovičová and Janeček, 2018). It was reported that GH13 including beta-galactosidase, beta-glucosidases, beta-xylosidases, and amylases exist in *Leuconostoc* sp. MTCC 10508 (Kaushal and Singh, 2020). The lysozyme which is belong to GH73 or GH25 was also found in 38 *Ln. pseudomesenteroides* genomes which is a catalyst of hydrolysis of beta (1–4) among N-acetylmuramic acid and N-acetyl glucosamine of cell wall and might possess antimicrobial potential (Michlmayr and Kneifel, 2014).

*In silico* evaluation of pangenome predicted 11 GT families that participate in carbohydrate metabolism. GT2 and GT4 family members represented the largest proportion of GTs in *Ln. pseudomesenteroides* genomes accounting for 42% and 32%, respectively and in alignment with previous observations for *Leuconostoc* genus (Sharma et al., 2022). Third largest member of GT family was GT51 involved in glycan metabolism. This enzymatic machinery degrades the complex polysaccharides of plants into mono- and oligosaccharides which could later be transported by ABC transporters (Sharma et al., 2022). GTs are functional in forming the glycosidic bonds by transferring sugar component from sugar donor (i.e., activated) to acceptor compound (Cantarel et al., 2009). GT51 which constitutes murein polymerases were also found in all *Ln. pseudomesenteroides* genomes. Murein polymerases are heavily participate in peptidoglycan synthesis and have a key role for maintaining the cell wall integrity (Sauvage et al., 2008). *Leuconostoc pseudomesenteroides* genomes carry crucial CAZymes that participate in carbohydrate hydrolysis and synthesis during fermentation. A bacterium’s carbohydrate fermentation potential is a critical indicator of biotechnological functionality of the strain and sets the fundamentals for strain selection and cultivation (Jiang et al., 2020).

**Figure 7:**
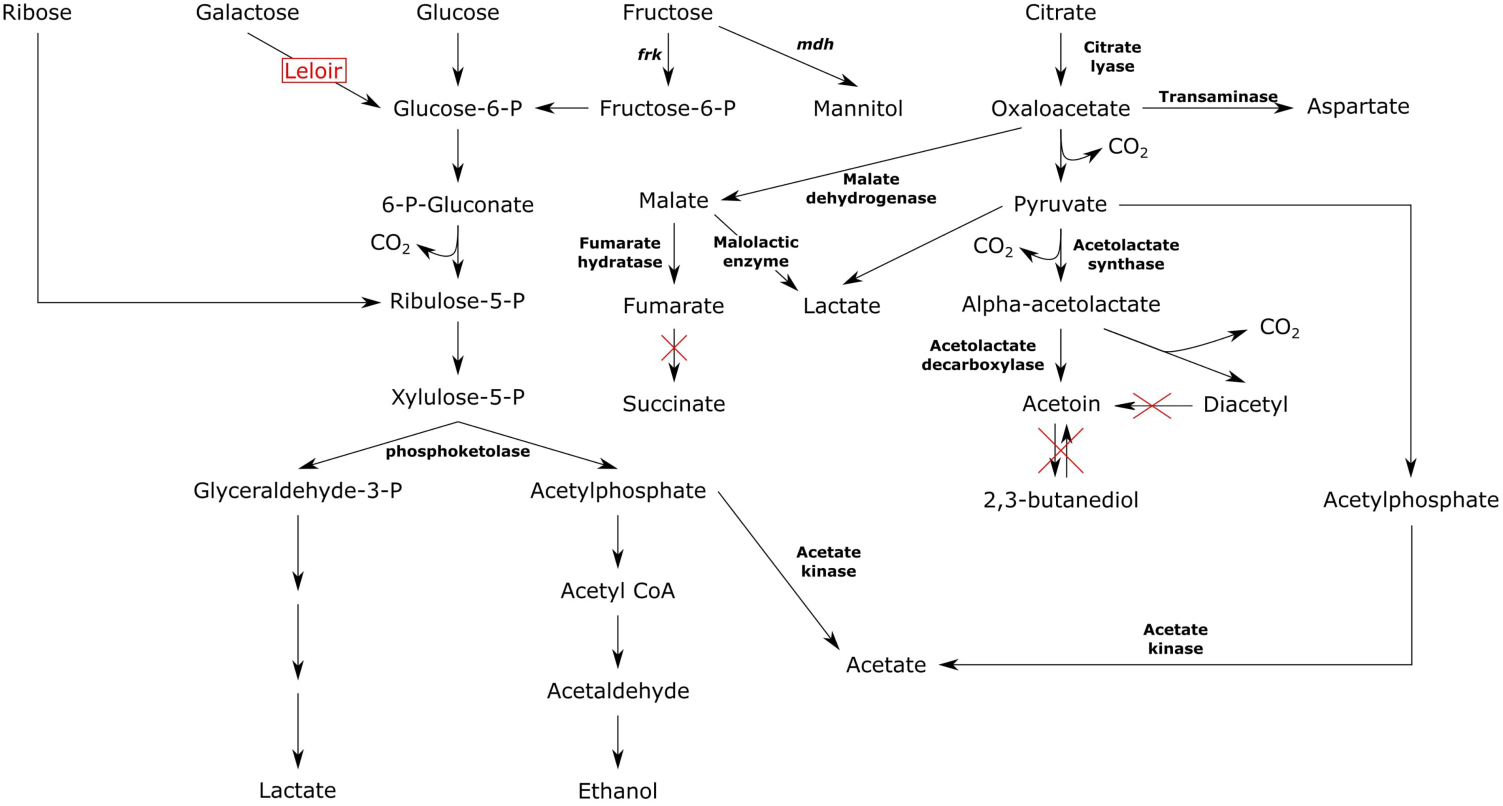
Putative carbohydrate and citrate metabolism pathway of *Ln. pseudomesenteroides.*

All *Ln. pseudomesenteroides* strains studied in the present work were predicted to carry phosphoketolase, a key enzyme in pentose phosphate pathway. The genus *Leuconostoc* is obligatory heterofermentative meaning six-carbon sugars are being utilized through pentose phosphate pathway also known as phosphoketolase pathway (Axelsson, 2004). Phosphofructokinase encoding gene was not found in any of the *Ln. pseudomesenteroides* genomes indicating Embden-Meyerhof pathway is not functional in this species (Frantzen et al., 2017). In contrast, fructose-bisphosphate aldolase encoding gene was available across all *Ln. pseudomesenteroides* genomes. This might imply a potential biosynthesis of fructose 1,6-bisphosphate and glyceraldehyde 3-phoshate via fructose 1-phosphate, therefore homofermentative cleavage of fructose in *Ln. pseudomesenteroides* (Frantzen et al., 2017). However, Grobben et al. (2001) reported that a mannitol producing *Ln. pseudomesenteroides* variant grown with sucrose could produce mannitol, CO_2_, lactate, acetate or ethanol through phosphoketolase shunt (Grobben et al., 2001). Putative fructose fermentation route in mannitol producing heterofermentative lactic acid bacteria is shown in Figure 7. In the presence of sucrose or fructose, *Ln. pseudomesenteroides* could potentially produce mannitol through mannitol dehydrogenase enzyme. Mannitol was reported to have osmoprotectant effect which improves the survival of dried *Lactococcus lactis* cells (Efiuvwevwere et al., 1999). Moreover, it is an antioxidant (Shen et al., 1997) and about 50% as sweet as sucrose thus considered to be a low calorie sweetener (Furia, 1972). Fructose could also be converted to fructose 6-phosphate by fructokinase at the expense of one ATP. Fructose 6-phosphate then feeds into pentose phosphate pathway because it lacks the 1-phosphofructokinase which is the key enzyme in Embden-Meyerhof pathway. Even though *Ln. pseudomesenteroides* carried fructose bisphosphate aldolase, the metabolism of hexose sugars such as fructose likely occurs through phosphoketolase pathway (Grobben et al., 2001).

While all *Ln. pseudomesenteroides* genomes contained *lacLM*, interestingly only TMW21195, a strain clustered with plant-associated isolates, harbored *lacZ*. To our knowledge, *lacZ* was not reported in *Ln. pseudomesenteroides* until present study as Frantzen et al. (2017) reported that *Ln. pseudomesenteroides* only encode beta-galactosidase through *lacLM* (Frantzen et al., 2017). The *lacZ* gene found in TMW21195 was severely truncated which might imply gene decay, a sign of prolonged degenerative evolution perhaps due to long period of growth in plant-based systems where no lactose exist. In *Leuconostoc* lactose is transported into the cytoplasm via *lacS*, lactose-specific transporter. *lacS* includes a C-terminal EIIAGlc-like domain and it can be phosphorylated leading an improved rate of lactose uptake in *Streptococcus thermophilus* (Gunnewijk and Poolman, 2000). All *Ln. pseudomesenteroides* isolates screened in the present study have *lacS* but in *Ln. cremoris* this gene is truncated and lacked C-terminal domain perhaps impacting lactose transport and thus growth rate on lactose (Frantzen et al., 2017).

Genetic potential for arabinose metabolism (*araBAD*) was only found in plant-associated isolates of CBA3630, Dm-9, FDAARGOS_1003, LMG 11482, LMG 11483, NCDO 768, TR070, and food isolates of TMW21073 and TMW21195. These isolates clustered together in PCoA plot and closely related in neighbor joining phylogenetic tree. None of dairy-associated *Ln. pseudomesenteroides* carried genes encoding for arabinose metabolism implying that these lineages are not capable of metabolizing arabinose from the environment. We speculate that dairy *Ln. pseudomesenteroides* might lost *araBAD* operon as a consequence of repetitive and prolonged period of growth in milk. Dextransucrase, functional in transformation of sucrose into fructose and dextran, is a crucial biotechnological trait of *Ln. pseudomesenteroides* as dextran contributes to textural and sensorial attributes of food systems by improving their viscosity and final stability (Duboc and Mollet, 2001). All plant-associated genomes evaluated in the present study contained complete dextransucrase gene except for Dm-9. However, all dairy-associated *Ln. pseudomesenteroides* strains possessed a deletion in the dextransucrase gene. The dairy *Ln. pseudomesenteroides* show telltale signs of prolonged degenerative evolution perhaps as a consequence of a long period of proliferation in milk where no sucrose exists.

All dairy-associated *Ln. pseudomesenteroides* genomes analyzed contained the *cit* operon composed of *citC*, *citDEF*, *citG*, *citO*, and *citS*. The existence of *citCDEFGOS* operon allows co-fermentation of citrate and sugar yielding increased proton motive force and energy yield to the cell (Marty-Teysset et al., 1996). This perhaps indicates that capability to metabolize citrate plays a crucial role in successful adaptation to milk environment (Frantzen et al., 2017). ~40% of non-dairy associated *Ln. pseudomesenteroides* strains lacked the complete operon for the citrate metabolism, perhaps a sign of evolutionary gene loss as a result of long period of proliferation in non-dairy related niches where no citrate exist.

Lactic acid bacteria produce acetoin, lactate, acetate, ethanol, succinate, and aspartate from citrate (Gänzle, 2015). The final products obtained from pyruvate was heavily relied on the pH such as acetoin formation favors low pH (Ramos et al., 1995). An elevated transformation of citrate or pyruvate to acetoin at low pH was reportedly shown in both heterofermentative (Drinan et al., 1976; Cogan et al., 1981) and homofermentative (McFall and Montville, 1989; Starrenburg and Hugenholtz, 1991) lactic acid bacteria. Citrate is first converted to oxaloacetic acid by citrate lyase and then transformed into pyruvate via oxaloacetate decarboxylase. Pyruvate is further decarboxylated to acetaldehyde-TPP after which it is converted to alpha-acetolactic acid by alpha-acetolactate synthase. Alpha-acetolactic acid can divert into acetoin or diacetyl. While conversion into acetoin requires alpha-acetolactate decarboxylase, transformation to diacetyl occurs through non-enzymatic decarboxylative oxidation of alpha-acetolactate. Acetoin can also be produced from diacetyl by diacetyl reductase (Ramos et al., 1995; Gänzle, 2015). *Leuconostoc pseudomesenteroides* could break down citrate to lactate, acetate, acetoin, and diacetyl due to carrying relevant enzymes required for such conversions. However, it is not capable of producing succinate and 2,3-butanediol because of not carrying succinate dehydrogenase and acetoin reductase, respectively (Figure 7). Moreover, none of the dairy-associated genomes were predicted to encode diacetyl reductase preventing conversion of diacetyl to acetoin. This was supported by previous studies that *Ln. pseudomesenteroides* P4 isolates missed the genes required for reduction of diacetyl to acetoin and 2,3-butanediol (Frantzen et al., 2017). The transformation of oxaloacetate to aspartate occurs in a single step reaction catalyzed by transaminase. Aspartate is the precursor of other amino acids such as asparagine, threonine, and methionine (Gottschalk, 1986). It was reported that aspartate is used to biosynthesize amino acids in *Ln. oenos* (Ramos et al., 1995). It was also reported that aspartate was not an essential amino acid for the proliferation of *Ln. oenos* when citrate and malate were supplemented to the growth medium (Amoroso et al., 1993), indicating that aspartate could be synthesized from citrate.

In dairy fermentations *Leuconostoc* spp. grow in relation with Lactococcus spp. It is not clear yet that whether the associative growth is of mutual interest to *Leuconostoc* spp. and *Lactococcus* spp. (Frantzen et al., 2017). Although *Leuconostoc* spp. was reported to grow poorly due to the lack of proteolytic activity (Thunell, 1995), Frantzen et al. (2017) described the genetic potential for caseinolytic activity and Cardamone et al. (2011) reported the capacity of milk acidification by *Ln. pseudomesenteroides* (Cardamone et al., 2011). It has been shown that *Ln. pseudomesenteroides* is an important species in the production of cheese (Frantzen et al., 2017).

Lactic acid bacteria use ADI pathway for transforming arginine into ornithine by citrulline and producing ATP and ammonia. The ammonia being produced elevates the pH and protects the bacteria against stressful acidic conditions (Cotter and Hill, 2003). We found that only 17–2 and TMW211195 carried putative *arcA* (arginine deiminase), *arcB* (ornithine transcarbamoylase), *arcC* (carbamate kinase), and *arcD* (arginine-ornithine transporter), which catalyze the ADI pathway (Figure 5). Although majority of *Ln. pseudomesenteroides* genomes analyzed in the present study encoded ornithine transcarbamoylase they lacked the remaining genes completing ADI metabolism.

Typically, strains from the same species would be highly similar. However, only about 12% of COGs were shared across 38 *Ln. pseudomesenteroides* strains would perhaps be explained by the presence of mobile genetic elements. It may also be due to inaccurate assemblies (Brandt et al., 2020). The putative mobilome of *Ln. pseudomesenteroides* provided a certain portion of plasmids, transposable elements, IS elements, and prophage-like elements. Despite mobilome accounting for a minor part of *Ln. pseudomesenteroides* genome, occasional transposition of these elements to the other regions of the genome is a significant contributor to genomic plasticity and the evolution of bacteria (Arber, 1991). The foreign DNA acquired from transposition results in the existence of the CRISPR-Cas system in variable sites of bacterial species (Li et al., 2021), which confers adaptive immunity to bacterial species to combat invasive elements (Barrangou et al., 2007).

Because the CRISPR-Cas system is an instrumental toolbox for Cas-based genome editing, we identified the presence and diversification of CRISPR in 38 *Ln. pseudomesenteroides* genomes. We found that 74% of genomes encoded a putative CRISPR system on a species level. This is larger than lactobacilli (62%) and bacteria (46%), suggesting that *Ln. pseudomesenteroides* could be a potential reservoir for new CRISPR-based tools (Sun et al., 2015). Type IIA CRISPR-Cas system was the primary and single type found in *Ln. pseudomesenteroides* genomes. Type IIA is the signature cas9 programmable endonuclease and the most useful CRISPR tool (Jinek et al., 2012). Generally, strains belonging to same species have similar vaccination records yielding sharing of spacers or similar spacer history (Brandt et al., 2020). We end up seeing limited shared spacer content across *Ln. pseudomesenteroides* strains. Of the putative type IIAs [LN23’s and LMGH100’s loci] or [LMGCF15’s, LMGCF06’s, and HPK01’s loci] or [LN02’s, LMGTW3’s, and LMGTW6’s loci] or [LMGH278’s and LMGH61’s loci] or [LMGH97’s and LMGTW1’s loci] or [IM1374’s and BM2’s loci] shared common spacer history. In a parallel manner, these genomes shared the same clade in the phylogenetic tree (Figure 3B). The distribution of these genomes on the phylogenetic tree implied the possible relationship between immunity system against exterior genetic material and the evolutionary route (Jiang et al., 2020). Despite the discrepancy in the spacer content, a relatively higher degree of similarity was observed in putative repeats of *Ln. pseudomesenteroides* (Figure 6B). This perhaps implies a hypervariability across *Ln. pseudomesenteroides* strains with regards to CRISPR and genomic rearrangements.

A few reports described bacteriocin production in *Ln. pseudomesenteroides* (Chen et al., 2018; Wang et al., 2018). We found three types of bacteriocins among 38 *Ln. pseudomesenteroides* strains, namely two unidentified bacteriocin-like structures in AMBR10 and CBA3630 and garvicin Q family class II bacteriocin in MGBC116435. Garvicin Q is a non-lantibiotic class II bacteriocin showing robust activity against Listeria spp and Lactococcus spp. (Tymoszewska et al., 2017). GarQ is a subclass IID bacteriocin biosynthesized by *Lactococcus garviae* BCC43578 isolated from sausage. Among the IS elements found in 38 *Ln. pseudomesenteroides*, both *Lactococcus garviae* and *Lactococcus lactis* appeared to be IS donors to MGBC116435. *Leuconostoc pseudomesenteroides* strains are used in mesophilic dairy starter co-cultures in conjunction with lactococci (Server-Busson et al., 1999). We speculate that *Ln. pseudomesenteroides* MGBC116435 acquired the garvicin Q biosynthesis capability as a competitive inhibition strategy, perhaps to compete with the competitor strain in the same ecological niche (Chaucheyras-Durand and Durand, 2010; Li et al., 2020). Since AMBR10 is a clinical isolate, we do not emphasize its bacteriocin-like structure owing to the potential pathogenicity of this strain. Bacteriocin-like structure found in CBA3630 and garvicin Q in MGBC116435 suggest that screening for unique antimicrobials requires further attention as a consequence of diverse microniches occupied by *Ln. pseudomesenteroides* strains and a large number of genes without known function.

## 5. Conclusion

Overall, the present study comparatively evaluated 38 *Ln. pseudomesenteroides* to determine genetic diversity across strains from different ecological niches and their biotechnological potential. Whole genome analysis demonstrated high genomic diversity across the strains, perhaps due to a large portion of accessory genomes, mobile genetic elements, and genes with unknown functions (i.e., hypothetical genes). Furthermore, comparative genomic analysis of the strains paves the way for describing ecological fitness to the host environment, for example, immunity against foreign DNA invasion through the CRISPR-Cas system and carbohydrate fermentation capacity differences seen between plant vs. non-plant associated strains. Only the plant-associated strains were predicted to carry arabinose sugar metabolism, which empowers the adaptation and survival of these strains in plant-associated environments. The present work explored the bacteriocin production capacity, evolutionary adaptation, and ecological fitness of *Ln. pseudomesenteroides* in the light of comparative genomics and enables genome-guided strain selection for industrial biomanufacturing. The findings of the current study set the baseline for the genetic characterization of *Ln. pseudomesenteroides* strains. Moreover, present work facilitates genome-guided strain selection with specific biotechnological features for industrial bioprocesses and creates a groundwork for characterizing traits of commercial relevance.

## Data availability statement

Publicly available datasets were analyzed in this study. The names of the repository/repositories and accession number(s) can be found in the article/Supplementary material.

## Author contributions

FO: conceptualization and supervision. FO and IG: investigation, data curation, formal analysis, visualization, and writing—original draft. All authors contributed to the article and approved the submitted version.

## Conflict of interest

The authors declare that the research was conducted in the absence of any commercial or financial relationships that could be construed as a potential conflict of interest.

## Publisher’s note

All claims expressed in this article are solely those of the authors and do not necessarily represent those of their affiliated organizations, or those of the publisher, the editors and the reviewers. Any product that may be evaluated in this article, or claim that may be made by its manufacturer, is not guaranteed or endorsed by the publisher.

## Supplementary material

The Supplementary material for this article can be found online at: https://www.frontiersin.org/articles/10.3389/fmicb.2022.1074366/full#supplementary-material
