# Supplementary material for: Analyzing the genetic diversity and biotechnological potential of Leuconostoc pseudomesenteroides by comparative genomics
Source: Front Microbiol. 2023 Jan 11;13:1074366. doi: 10.3389/fmicb.2022.1074366 (PMC9875049; doi:10.3389/fmicb.2022.1074366)
Supplement: Supplementary file 1 [file Table_1.DOCX]

***Supplementary Files***

**Supplementary Table 1.** Putative bacteriocin biosynthesis in *Ln. pseudomesenteroides*

| Strain | Name | Start | End | Strand |
| --- | --- | --- | --- | --- |
| AMBR10 | Undefined bacteriocin-like structure | 1151955 | 1152446 | + |
| CBA3630 | Undefined bacteriocin-like structure | 2264404 | 2264895 | + |
| MGBC116435 | garvicin Q family class II bacteriocin | 2061491 | 2061664 | + |

**Supplementary Table 2.** Putative plasmids found in *Ln. pseudomesenteroides*

| **Strain** | **Identity** | **p-value** | **Accession Number** | **Topology** | **Length (bp)** |
| --- | --- | --- | --- | --- | --- |
| 1159 | 0.994253 | 0 | NC_006145.1 | circular | 1828 |
| 1159 | 0.998941 | 0 | NZ_CP065974.1 | circular | 22838 |
| 1159 | 0.996805 | 0 | NZ_CP065977.1 | circular | 27304 |
| 1159 | 0.99776 | 0 | NZ_CP065979.1 | circular | 1817 |
| AMBR10 | 0.998157 | 0 | NZ_CP042384.1 | circular | 34525 |
| BM2 | 0.996601 | 0 | NC_006145.1 | circular | 1828 |
| BM2 | 0.999425 | 0 | NZ_CP065973.1 | circular | 43806 |
| BM2 | 1 | 0 | NZ_CP065974.1 | circular | 22838 |
| BM2 | 1 | 0 | NZ_CP065979.1 | circular | 1817 |
| CBA3630 | 1 | 0 | NZ_CP042384.1 | circular | 34525 |
| CBA3630 | 1 | 0 | NZ_CP042385.1 | circular | 23962 |
| CBA3630 | 1 | 0 | NZ_CP042386.1 | circular | 23536 |
| FDAARGOS_1004 | 0.995779 | 0 | NC_006145.1 | circular | 1828 |
| FDAARGOS_1004 | 1 | 0 | NZ_CP065973.1 | circular | 43806 |
| FDAARGOS_1004 | 1 | 0 | NZ_CP065974.1 | circular | 22838 |
| FDAARGOS_1004 | 1 | 0 | NZ_CP065976.1 | circular | 21876 |
| FDAARGOS_1004 | 1 | 0 | NZ_CP065977.1 | circular | 27304 |
| FDAARGOS_1004 | 1 | 0 | NZ_CP065978.1 | linear | 24161 |
| FDAARGOS_1004 | 1 | 0 | NZ_CP065979.1 | circular | 1817 |
| HPK01 | 0.994626 | 0 | NZ_CP065976.1 | circular | 21876 |
| IM1374 | 0.999809 | 0 | NZ_CP065973.1 | circular | 43806 |
| IM1374 | 1 | 0 | NZ_CP065974.1 | circular | 22838 |
| IM1374 | 0.999809 | 0 | NZ_CP065976.1 | circular | 21876 |
| IM1374 | 0.999713 | 0 | NZ_CP065977.1 | circular | 27304 |
| IM1427 | 0.998649 | 0 | NZ_CP065976.1 | circular | 21876 |
| IM1427 | 0.999713 | 0 | NZ_CP065977.1 | circular | 27304 |
| LMGCF06 | 0.99741 | 0 | NZ_CP065976.1 | circular | 21876 |
| LMGCF15 | 0.997108 | 0 | NZ_CP065976.1 | circular | 21876 |
| LMGH100 | 0.998941 | 0 | NZ_CP065974.1 | circular | 22838 |
| LMGH278 | 0.997108 | 0 | NZ_CP065976.1 | circular | 21876 |
| LMGH280 | 0.999522 | 0 | NZ_CP065976.1 | circular | 21876 |
| LMGH284 | 0.999761 | 0 | NZ_CP065974.1 | circular | 22838 |
| LMGH61 | 0.999522 | 0 | NZ_CP065976.1 | circular | 21876 |
| LMGH83 | 0.999761 | 0 | NZ_CP065974.1 | circular | 22838 |
| LMGH95 | 0.99766 | 0 | NZ_CP065974.1 | circular | 22838 |
| LMGH97 | 0.99756 | 0 | NZ_CP065973.1 | circular | 43806 |
| LMGH97 | 0.996397 | 0 | NZ_CP065974.1 | circular | 22838 |
| LMGH97 | 0.99771 | 0 | NZ_CP065976.1 | circular | 21876 |
| LMGH97 | 0.993607 | 0 | NZ_CP065977.1 | circular | 27304 |
| LMGTW1 | 0.99726 | 0 | NZ_CP065973.1 | circular | 43806 |
| LMGTW1 | 0.999135 | 0 | NZ_CP065976.1 | circular | 21876 |
| LMGTW1 | 0.994039 | 0 | NZ_CP065977.1 | circular | 27304 |
| LMGTW3 | 0.998403 | 0 | NZ_CP065974.1 | circular | 22838 |
| LMGTW3 | 0.995675 | 0 | NZ_CP065976.1 | circular | 21876 |
| LMGTW3 | 0.995519 | 0 | NZ_CP065977.1 | circular | 27304 |
| LMGTW6 | 0.99771 | 0 | NZ_CP065974.1 | circular | 22838 |
| LMGTW6 | 0.995258 | 0 | NZ_CP065976.1 | circular | 21876 |
| LMGTW6 | 0.996294 | 0 | NZ_CP065977.1 | circular | 27304 |
| LMGTW8 | 0.996906 | 0 | NZ_CP065973.1 | circular | 43806 |
| LMGTW8 | 0.99761 | 0 | NZ_CP065974.1 | circular | 22838 |
| LMGTW8 | 0.99655 | 0 | NZ_CP065976.1 | circular | 21876 |
| LMGTW8 | 0.994943 | 0 | NZ_CP065977.1 | circular | 27304 |
| LN02 | 0.998305 | 0 | NZ_CP065974.1 | circular | 22838 |
| LN02 | 0.997007 | 0 | NZ_CP065976.1 | circular | 21876 |
| LN02 | 0.995363 | 0 | NZ_CP065977.1 | circular | 27304 |
| LN12 | 0.996906 | 0 | NZ_CP065976.1 | circular | 21876 |
| LN12 | 0.99531 | 0 | NZ_CP065977.1 | circular | 27304 |
| LN23 | 0.999761 | 0 | NZ_CP065974.1 | circular | 22838 |
| PS12 | 0.999522 | 0 | NZ_CP065973.1 | circular | 43806 |
| PS12 | 0.999952 | 0 | NZ_CP065974.1 | circular | 22838 |
| PS12 | 0.999329 | 0 | NZ_CP065976.1 | circular | 21876 |
| PS12 | 0.99756 | 0 | NZ_CP065977.1 | circular | 27304 |

**Supplementary Table 3.** Insertion sequences predicted in *Ln. pseudomesenteroides* strains.

| **Strain** | **Sequences producing significant alignments** | **IS Family** | **Group** | **Origin** | **Score (bits)** | **E. value** |
| --- | --- | --- | --- | --- | --- | --- |
| 1559 | ISLhe30 | IS30 |  | Lactobacillus helveticus | 1558 | 0 |
| 1559 | IS1070 | IS30 |  | Leuconostoc lactis | 807 | 0 |
| 1559 | ISWci2 | IS3 | IS3 | Weissella cibaria | 799 | 0 |
| 1559 | ISLhe11 | ISLre2 |  | Lactobacillus helveticus | 763 | 0 |
| 1559 | ISLhe10 | ISLre2 |  | Lactobacillus helveticus | 519 | 6E-144 |
| 1559 | ISLhe13 | ISLre2 |  | Lactobacillus helveticus | 456 | 7E-125 |
| 1559 | ISLke1 | ISLre2 |  | Lactobacillus kefiranofaciens | 440 | 4E-120 |
| 1559 | ISLcr1 | ISLre2 |  | Lactobacillus crispatus | 387 | 5E-104 |
| 1559 | ISLpl1 | IS30 |  | Lactobacillus plantarum | 232 | 2E-57 |
| 1559 | ISPp1 | IS30 |  | Pediococcus pentosaceus | 184 | 4E-43 |
| 1559 | ISLcr2 | ISLre2 |  | Lactobacillus crispatus | 137 | 8E-29 |
| 1559 | ISLde1 | ISLre2 |  | Lactobacillus delbrueckii | 135 | 3E-28 |
| 1559 | ISLmo16 | IS3 | IS150 | Listeria monocytogenes | 61.9 | 0.000004 |
| 1559 | ISBwe2 | IS6 |  | Bacillus weihenstephanensis | 56.0 | 0.0002 |
| 1559 | ISBwe3 | IS6 |  | Bacillus weihenstephanensis | 54.0 | 0.001 |
| 4882 | IS1163 | IS3 | IS3 | Lactobacillus sake | 2230 | 0 |
| 4882 | IS1310 | IS256 |  | Enterococcus hirae | 2157 | 0 |
| 4882 | IS1070 | IS30 |  | Leuconostoc lactis | 1925 | 0 |
| 4882 | ISLhe30 | IS30 |  | Lactobacillus helveticus | 1566 | 0 |
| 4882 | ISWci2 | IS3 | IS3 | Weissella cibaria | 1072 | 0 |
| 4882 | ISLhe11 | ISLre2 |  | Lactobacillus helveticus | 763 | 0 |
| 4882 | ISLhe10 | ISLre2 |  | Lactobacillus helveticus | 519 | 5E-144 |
| 4882 | ISLhe13 | ISLre2 |  | Lactobacillus helveticus | 456 | 7E-125 |
| 4882 | ISLke1 | ISLre2 |  | Lactobacillus kefiranofaciens | 440 | 4E-120 |
| 4882 | ISLcr1 | ISLre2 |  | Lactobacillus crispatus | 387 | 5E-104 |
| 4882 | ISS1A | IS6 |  | Lactococcus lactis | 230 | 7E-57 |
| 4882 | ISS1E | IS6 |  | Lactococcus lactis | 226 | 1E-55 |
| 4882 | ISS1N | IS6 |  | Lactococcus lactis | 222 | 2E-54 |
| 4882 | IS1297 | IS6 |  | Leuconostoc mesenteroides | 222 | 2E-54 |
| 4882 | ISS1D | IS6 |  | Lactococcus lactis | 214 | 4E-52 |
| 4882 | ISS1M | IS6 |  | Lactococcus lactis | 204 | 4E-49 |
| 4882 | ISS1CH | IS6 |  | Lactococcus lactis | 204 | 4E-49 |
| 4882 | ISLcr2 | ISLre2 |  | Lactobacillus crispatus | 137 | 8E-29 |
| 4882 | ISLde1 | ISLre2 |  | Lactobacillus delbrueckii | 135 | 3E-28 |
| 4882 | ISS1X | IS6 |  | Lactococcus lactis | 89.7 | 2E-14 |
| 4882 | ISS1T | IS6 |  | Lactococcus lactis | 89.7 | 2E-14 |
| 4882 | ISS1S | IS6 |  | Lactococcus lactis | 89.7 | 2E-14 |
| 4882 | ISS1RS | IS6 |  | Lactococcus lactis | 89.7 | 2E-14 |
| 4882 | IS946V | IS6 |  | Lactococcus lactis | 87.7 | 7E-14 |
| 4882 | ISS1B | IS6 |  | Lactococcus lactis | 81.8 | 4E-12 |
| 4882 | ISS1Z | IS6 |  | Lactococcus lactis | 67.9 | 0.00000006 |
| 4882 | ISLmo16 | IS3 | IS150 | Listeria monocytogenes | 61.9 | 0.000004 |
| 4882 | ISTeha2 | IS6 |  | Tetragenococcus halophilus | 60.0 | 0.00002 |
| 4882 | ISBwe2 | IS6 |  | Bacillus weihenstephanensis | 56.0 | 0.0002 |
| 4882 | ISBwe3 | IS6 |  | Bacillus weihenstephanensis | 54.0 | 0.001 |
| 4882 | ISLmo4 | IS6 |  | Listeria monocytogenes | 52.0 | 0.004 |
| 17-2 | IS1070 | IS30 |  | Leuconostoc lactis | 176 | 1E-40 |
| 17-2 | ISCth3 | IS30 |  | Clostridium thermocellum | 52.0 | 0.004 |
| AMBR10 | IS1163 | IS3 | IS3 | Lactobacillus sake | 2252 | 0 |
| AMBR10 | ISLpl1 | IS30 |  | Lactobacillus plantarum | 1925 | 0 |
| AMBR10 | ISPp1 | IS30 |  | Pediococcus pentosaceus | 1853 | 0 |
| AMBR10 | IS1070 | IS30 |  | Leuconostoc lactis | 1600 | 0 |
| AMBR10 | ISWci2 | IS3 | IS3 | Weissella cibaria | 1241 | 0 |
| AMBR10 | IS15DII | IS6 |  | Salmonella panama | 1009 | 0 |
| AMBR10 | IS15 | IS6 |  | Salmonella panama | 1009 | 0 |
| AMBR10 | IS26 | IS6 |  | Proteus vulgaris | 1009 | 0 |
| AMBR10 | IS15DIV | IS6 |  | Salmonella typhimurium | 1001 | 0 |
| AMBR10 | IS15DI | IS6 |  | Salmonella panama | 1001 | 0 |
| AMBR10 | IS1SD | IS1 |  | Shigella dysenteriae | 900 | 0 |
| AMBR10 | ISEc23 | IS66 |  | Escherichia coli | 896 | 0 |
| AMBR10 | IS1R | IS1 |  | Escherichia coli | 892 | 0 |
| AMBR10 | IS1G | IS1 |  | Escherichia coli | 892 | 0 |
| AMBR10 | IS1D | IS1 |  | Escherichia coli | 892 | 0 |
| AMBR10 | IS1B | IS1 |  | Escherichia coli | 892 | 0 |
| AMBR10 | IS1A | IS1 |  | Escherichia coli | 892 | 0 |
| AMBR10 | IS1S | IS1 |  | Shigella sonnei | 884 | 0 |
| AMBR10 | IS621 | IS110 |  | Escherichia coli | 779 | 0 |
| AMBR10 | IS4 | IS4 | IS4 | Escherichia coli | 741 | 0 |
| AMBR10 | ISEc47 | IS66 |  | Escherichia coli | 682 | 0 |
| AMBR10 | ISSen9 | IS1 |  | Salmonella enterica | 676 | 0 |
| AMBR10 | IS1X2 | IS1 |  | Escherichia vulneris | 668 | 0 |
| AMBR10 | ISShdy4 | IS66 |  | Shigella dysenteriae | 666 | 0 |
| AMBR10 | ISEc43 | IS66 |  | Escherichia coli | 658 | 0 |
| AMBR10 | ISSen15 | IS6 |  | Salmonella enterica | 615 | 1E-172 |
| AMBR10 | IS1X4 | IS1 |  | Escherichia hermannii | 615 | 1E-172 |
| AMBR10 | IS1X1 | IS1 |  | Shigella flexneri | 605 | 1E-169 |
| AMBR10 | ISEc17 | IS3 | IS3 | Escherichia coli | 595 | 1E-166 |
| AMBR10 | IS3 | IS3 | IS3 | Escherichia coli | 595 | 1E-166 |
| AMBR10 | IS1F | IS1 |  | Escherichia coli | 589 | 8E-165 |
| AMBR10 | IS1X3 | IS1 |  | Escherichia fergusonii | 575 | 1E-160 |
| AMBR10 | IS682 | IS66 |  | Escherichia coli | 571 | 2E-159 |
| AMBR10 | IS3H | IS3 | IS3 | Shigella dysenteriae | 533 | 4E-148 |
| AMBR10 | IS3F | IS3 | IS3 | Escherichia fergusonii | 490 | 6E-135 |
| AMBR10 | ISKpn14 | IS1 |  | Klebsiella pneumoniae | 416 | 7E-113 |
| AMBR10 | ISCro4 | IS110 |  | Citrobacter rodentium | 311 | 3E-81 |
| AMBR10 | ISPcc3 | IS4 | IS4 | Pectobacterium carotovorum | 204 | 5E-49 |
| AMBR10 | ISEal1 | IS3 | IS3 | Escherichia albertii | 155 | 4E-34 |
| AMBR10 | ISKox1 | IS66 |  | Klebsiella oxytoca | 153 | 2E-33 |
| AMBR10 | IS1006 | IS6 |  | Acinetobacter junii | 153 | 2E-33 |
| AMBR10 | ISEc16 | IS3 | IS3 | Escherichia coli | 147 | 1E-31 |
| AMBR10 | ISEsp1 | IS66 |  | Enterobacter sp. | 145 | 4E-31 |
| AMBR10 | ISAba52 | IS6 |  | Acinetobacter baumannii | 137 | 9E-29 |
| AMBR10 | IS1007 | IS6 |  | Acinetobacter sp. | 137 | 9E-29 |
| AMBR10 | ISSty2 | IS3 | IS3 | Salmonella typhimurium | 133 | 1E-27 |
| AMBR10 | ISOur1 | IS6 |  | Oligella urethralis | 121 | 5E-24 |
| AMBR10 | IS1008 | IS6 |  | Acinetobacter calcoaceticus | 113 | 1E-21 |
| AMBR10 | ISKpn33 | IS66 |  | Klebsiella pneumoniae | 111 | 5E-21 |
| AMBR10 | IS1230B | IS3 | IS3 | Salmonella enteritidis | 109 | 2E-20 |
| AMBR10 | ISEcl6 | IS66 |  | Enterobacter cloacae | 95.6 | 3E-16 |
| AMBR10 | ISPeat2 | IS3 | IS3 | Pectobacterium atrosepticum | 91.7 | 5E-15 |
| AMBR10 | ISStag1 | IS6 |  | Stappia aggregata | 65.9 | 0.0000003 |
| AMBR10 | ISLmo16 | IS3 | IS150 | Listeria monocytogenes | 61.9 | 0.000004 |
| AMBR10 | ISAcr2 | IS6 |  | Acidiphilium cryptum | 58.0 | 0.00007 |
| AMBR10 | ISBwe2 | IS6 |  | Bacillus weihenstephanensis | 56.0 | 0.0003 |
| AMBR10 | ISSba6 | IS4 | IS4 | Shewanella baltica | 52.0 | 0.004 |
| AMBR10 | ISBwe3 | IS6 |  | Bacillus weihenstephanensis | 52.0 | 0.004 |
| BM2 | IS1070 | IS30 |  | Leuconostoc lactis | 1917 | 0 |
| BM2 | ISLhe30 | IS30 |  | Lactobacillus helveticus | 1550 | 0 |
| BM2 | ISLhe11 | ISLre2 |  | Lactobacillus helveticus | 763 | 0 |
| BM2 | ISLhe10 | ISLre2 |  | Lactobacillus helveticus | 519 | 5E-144 |
| BM2 | ISLhe13 | ISLre2 |  | Lactobacillus helveticus | 456 | 7E-125 |
| BM2 | ISLke1 | ISLre2 |  | Lactobacillus kefiranofaciens | 440 | 4E-120 |
| BM2 | ISLcr1 | ISLre2 |  | Lactobacillus crispatus | 387 | 5E-104 |
| BM2 | ISWci2 | IS3 | IS3 | Weissella cibaria | 204 | 4E-49 |
| BM2 | ISLcr2 | ISLre2 |  | Lactobacillus crispatus | 137 | 8E-29 |
| BM2 | ISLde1 | ISLre2 |  | Lactobacillus delbrueckii | 135 | 3E-28 |
| BM2 | ISLmo16 | IS3 | IS150 | Listeria monocytogenes | 61.9 | 0.000004 |
| BM2 | ISBwe2 | IS6 |  | Bacillus weihenstephanensis | 56.0 | 0.0002 |
| BM2 | ISBwe3 | IS6 |  | Bacillus weihenstephanensis | 54.0 | 0.001 |
| CBA3630 | IS1070 | IS30 |  | Leuconostoc lactis | 1885 | 0 |
| CBA3630 | ISWci2 | IS3 | IS3 | Weissella cibaria | 1249 | 0 |
| CBA3630 | ISLpl1 | IS30 |  | Lactobacillus plantarum | 480 | 5E-132 |
| CBA3630 | ISPp1 | IS30 |  | Pediococcus pentosaceus | 432 | 1E-117 |
| Dm-9 | IS1070 | IS30 |  | Leuconostoc lactis | 184 | 4E-43 |
| Dm-9 | IS1165 | ISL3 |  | Leuconostoc mesenteroides | 115 | 3E-22 |
| Dm-9 | ISCth3 | IS30 |  | Clostridium thermocellum | 56.0 | 0.0003 |
| FDAARGOS_1003 | IS1070 | IS30 |  | Leuconostoc lactis | 180 | 6E-42 |
| FDAARGOS_1004 | IS1070 | IS30 |  | Leuconostoc lactis | 2036 | 0 |
| FDAARGOS_1004 | ISLhe30 | IS30 |  | Lactobacillus helveticus | 1558 | 0 |
| FDAARGOS_1004 | ISWci2 | IS3 | IS3 | Weissella cibaria | 1049 | 0 |
| FDAARGOS_1004 | ISLhe11 | ISLre2 |  | Lactobacillus helveticus | 553 | 4E-154 |
| FDAARGOS_1004 | ISLhe10 | ISLre2 |  | Lactobacillus helveticus | 519 | 6E-144 |
| FDAARGOS_1004 | ISLhe13 | ISLre2 |  | Lactobacillus helveticus | 456 | 7E-125 |
| FDAARGOS_1004 | ISLke1 | ISLre2 |  | Lactobacillus kefiranofaciens | 440 | 4E-120 |
| FDAARGOS_1004 | ISLcr1 | ISLre2 |  | Lactobacillus crispatus | 387 | 5E-104 |
| FDAARGOS_1004 | ISLpl1 | IS30 |  | Lactobacillus plantarum | 232 | 2E-57 |
| FDAARGOS_1004 | ISPp1 | IS30 |  | Pediococcus pentosaceus | 184 | 4E-43 |
| FDAARGOS_1004 | ISLcr2 | ISLre2 |  | Lactobacillus crispatus | 137 | 8E-29 |
| FDAARGOS_1004 | ISLde1 | ISLre2 |  | Lactobacillus delbrueckii | 135 | 3E-28 |
| FDAARGOS_1004 | ISLmo16 | IS3 | IS150 | Listeria monocytogenes | 61.9 | 0.000004 |
| FDAARGOS_1004 | ISBwe2 | IS6 |  | Bacillus weihenstephanensis | 56.0 | 0.0002 |
| FDAARGOS_1004 | ISBwe3 | IS6 |  | Bacillus weihenstephanensis | 54.0 | 0.001 |
| HPK01 | ISLhe30 | IS30 |  | Lactobacillus helveticus | 1566 | 0 |
| HPK01 | IS1070 | IS30 |  | Leuconostoc lactis | 1164 | 0 |
| HPK01 | ISWci2 | IS3 | IS3 | Weissella cibaria | 652 | 0 |
| HPK01 | ISLpl1 | IS30 |  | Lactobacillus plantarum | 232 | 2E-57 |
| HPK01 | ISPp1 | IS30 |  | Pediococcus pentosaceus | 184 | 4E-43 |
| HPK01 | ISLmo16 | IS3 | IS150 | Listeria monocytogenes | 61.9 | 0.000004 |
| IM1374 | IS1070 | IS30 |  | Leuconostoc lactis | 1933 | 0 |
| IM1374 | ISLhe30 | IS30 |  | Lactobacillus helveticus | 1558 | 0 |
| IM1374 | ISWci2 | IS3 | IS3 | Weissella cibaria | 805 | 0 |
| IM1374 | ISLhe11 | ISLre2 |  | Lactobacillus helveticus | 763 | 0 |
| IM1374 | ISLhe10 | ISLre2 |  | Lactobacillus helveticus | 519 | 6E-144 |
| IM1374 | ISLhe13 | ISLre2 |  | Lactobacillus helveticus | 456 | 7E-125 |
| IM1374 | ISLke1 | ISLre2 |  | Lactobacillus kefiranofaciens | 440 | 4E-120 |
| IM1374 | ISLcr1 | ISLre2 |  | Lactobacillus crispatus | 387 | 5E-104 |
| IM1374 | ISLpl1 | IS30 |  | Lactobacillus plantarum | 232 | 2E-57 |
| IM1374 | ISPp1 | IS30 |  | Pediococcus pentosaceus | 184 | 4E-43 |
| IM1374 | ISLcr2 | ISLre2 |  | Lactobacillus crispatus | 137 | 8E-29 |
| IM1374 | ISLde1 | ISLre2 |  | Lactobacillus delbrueckii | 135 | 3E-28 |
| IM1374 | ISLmo16 | IS3 | IS150 | Listeria monocytogenes | 61.9 | 0.000004 |
| IM1374 | ISBwe2 | IS6 |  | Bacillus weihenstephanensis | 56.0 | 0.0002 |
| IM1374 | ISBwe3 | IS6 |  | Bacillus weihenstephanensis | 54.0 | 0.001 |
| IM1427 | ISLpl1 | IS30 |  | Lactobacillus plantarum | 1972 | 0 |
| IM1427 | IS1070 | IS30 |  | Leuconostoc lactis | 1925 | 0 |
| IM1427 | ISPp1 | IS30 |  | Pediococcus pentosaceus | 1877 | 0 |
| IM1427 | IS1165 | ISL3 |  | Leuconostoc mesenteroides | 1798 | 0 |
| IM1427 | ISLhe30 | IS30 |  | Lactobacillus helveticus | 1558 | 0 |
| IM1427 | ISWci2 | IS3 | IS3 | Weissella cibaria | 805 | 0 |
| IM1427 | ISLmo16 | IS3 | IS150 | Listeria monocytogenes | 61.9 | 0.000004 |
| KCTC3652 | IS1070 | IS30 |  | Leuconostoc lactis | 1035 | 0 |
| KCTC3652 | ISWci2 | IS3 | IS3 | Weissella cibaria | 601 | 3E-168 |
| KCTC3652 | ISLpl1 | IS30 |  | Lactobacillus plantarum | 321 | 4E-84 |
| KCTC3652 | ISPp1 | IS30 |  | Pediococcus pentosaceus | 321 | 4E-84 |
| KMB610 | IS1070 | IS30 |  | Leuconostoc lactis | 1871 | 0 |
| KMB610 | ISLpl1 | IS30 |  | Lactobacillus plantarum | 1822 | 0 |
| KMB610 | ISPp1 | IS30 |  | Pediococcus pentosaceus | 1766 | 0 |
| KMB610 | ISWci2 | IS3 | IS3 | Weissella cibaria | 1076 | 0 |
| KMB610 | ISLmo16 | IS3 | IS150 | Listeria monocytogenes | 61.9 | 0.000004 |
| KMB610 | ISBwe2 | IS6 |  | Bacillus weihenstephanensis | 56.0 | 0.0002 |
| KMB610 | ISBwe3 | IS6 |  | Bacillus weihenstephanensis | 52.0 | 0.004 |
| LMG_11482 | IS1070 | IS30 |  | Leuconostoc lactis | 180 | 6E-42 |
| LMG_11483 | ISWci2 | IS3 | IS3 | Weissella cibaria | 1689 | 0 |
| LMG_11483 | IS153 | IS3 | IS3 | Lactobacillus sanfranciscensis | 1158 | 0 |
| LMG_11483 | IS1070 | IS30 |  | Leuconostoc lactis | 662 | 0 |
| LMGCF06 | ISLhe30 | IS30 |  | Lactobacillus helveticus | 1566 | 0 |
| LMGCF06 | IS1070 | IS30 |  | Leuconostoc lactis | 1164 | 0 |
| LMGCF06 | ISWci2 | IS3 | IS3 | Weissella cibaria | 654 | 0 |
| LMGCF06 | ISLpl1 | IS30 |  | Lactobacillus plantarum | 232 | 2E-57 |
| LMGCF06 | ISPp1 | IS30 |  | Pediococcus pentosaceus | 184 | 4E-43 |
| LMGCF06 | ISLmo16 | IS3 | IS150 | Listeria monocytogenes | 61.9 | 0.000004 |
| LMGCF08 | IS1165 | ISL3 |  | Leuconostoc mesenteroides | 1978 | 0 |
| LMGCF08 | ISWci2 | IS3 | IS3 | Weissella cibaria | 1719 | 0 |
| LMGCF08 | ISLhe30 | IS30 |  | Lactobacillus helveticus | 1566 | 0 |
| LMGCF08 | IS1297 | IS6 |  | Leuconostoc mesenteroides | 1546 | 0 |
| LMGCF08 | ISS1N | IS6 |  | Lactococcus lactis | 1499 | 0 |
| LMGCF08 | ISS1E | IS6 |  | Lactococcus lactis | 1487 | 0 |
| LMGCF08 | ISS1M | IS6 |  | Lactococcus lactis | 1465 | 0 |
| LMGCF08 | ISS1D | IS6 |  | Lactococcus lactis | 1451 | 0 |
| LMGCF08 | ISS1CH | IS6 |  | Lactococcus lactis | 1390 | 0 |
| LMGCF08 | ISLll1 | IS982 |  | Lactococcus lactis | 1193 | 0 |
| LMGCF08 | IS982C | IS982 |  | Lactococcus lactis | 1178 | 0 |
| LMGCF08 | IS982B | IS982 |  | Lactococcus lactis | 1170 | 0 |
| LMGCF08 | IS1070 | IS30 |  | Leuconostoc lactis | 1164 | 0 |
| LMGCF08 | IS982 | IS982 |  | Lactococcus lactis | 1162 | 0 |
| LMGCF08 | IS153 | IS3 | IS3 | Lactobacillus sanfranciscensis | 1094 | 0 |
| LMGCF08 | ISS1A | IS6 |  | Lactococcus lactis | 858 | 0 |
| LMGCF08 | ISLla2 | IS982 |  | Lactococcus lactis | 844 | 0 |
| LMGCF08 | IS946V | IS6 |  | Lactococcus lactis | 842 | 0 |
| LMGCF08 | ISS1T | IS6 |  | Lactococcus lactis | 628 | 9E-177 |
| LMGCF08 | ISS1S | IS6 |  | Lactococcus lactis | 605 | 1E-169 |
| LMGCF08 | ISS1RS | IS6 |  | Lactococcus lactis | 605 | 1E-169 |
| LMGCF08 | ISS1B | IS6 |  | Lactococcus lactis | 605 | 1E-169 |
| LMGCF08 | ISS1X | IS6 |  | Lactococcus lactis | 597 | 3E-167 |
| LMGCF08 | ISLgar4 | IS6 |  | Lactococcus garvieae | 593 | 5E-166 |
| LMGCF08 | ISS1Z | IS6 |  | Lactococcus lactis | 549 | 7E-153 |
| LMGCF08 | ISLgar2 | IS982 |  | Lactococcus garvieae | 458 | 2E-125 |
| LMGCF08 | ISLpl1 | IS30 |  | Lactobacillus plantarum | 232 | 2E-57 |
| LMGCF08 | ISS1W | IS6 |  | Lactococcus lactis | 208 | 3E-50 |
| LMGCF08 | IS1216E | IS6 |  | Enterococcus faecium | 198 | 3E-47 |
| LMGCF08 | ISWco1 | IS3 | IS150 | Weissella confusa | 196 | 1E-46 |
| LMGCF08 | ISPp1 | IS30 |  | Pediococcus pentosaceus | 184 | 4E-43 |
| LMGCF08 | IS1216V | IS6 |  | Enterococcus sp. | 182 | 2E-42 |
| LMGCF08 | IS1216 | IS6 |  | Enterococcus hirae | 167 | 1E-37 |
| LMGCF08 | ISTeha2 | IS6 |  | Tetragenococcus halophilus | 155 | 4E-34 |
| LMGCF08 | IS1310 | IS256 |  | Enterococcus hirae | 155 | 4E-34 |
| LMGCF08 | ISLmo14 | IS6 |  | Listeria monocytogenes | 137 | 9E-29 |
| LMGCF08 | ISLmo13 | IS6 |  | Listeria monocytogenes | 125 | 3E-25 |
| LMGCF08 | ISEnfa1 | IS6 |  | Enterococcus faecium | 125 | 3E-25 |
| LMGCF08 | ISLmo19 | IS6 |  | Listeria monocytogenes | 117 | 8E-23 |
| LMGCF08 | ISLmo16 | IS3 | IS150 | Listeria monocytogenes | 61.9 | 0.000004 |
| LMGCF08 | ISLmo4 | IS6 |  | Listeria monocytogenes | 61.9 | 0.000004 |
| LMGCF08 | ISBame1 | IS256 |  | Bacillus megaterium | 58.0 | 0.00007 |
| LMGCF08 | ISLmo3 | IS6 |  | Listeria monocytogenes | 56.0 | 0.0003 |
| LMGCF15 | ISLhe30 | IS30 |  | Lactobacillus helveticus | 1566 | 0 |
| LMGCF15 | IS1070 | IS30 |  | Leuconostoc lactis | 1164 | 0 |
| LMGCF15 | ISWci2 | IS3 | IS3 | Weissella cibaria | 652 | 0 |
| LMGCF15 | ISLpl1 | IS30 |  | Lactobacillus plantarum | 232 | 2E-57 |
| LMGCF15 | ISPp1 | IS30 |  | Pediococcus pentosaceus | 184 | 4E-43 |
| LMGCF15 | ISLmo16 | IS3 | IS150 | Listeria monocytogenes | 61.9 | 0.000004 |
| LMGH100 | IS1163 | IS3 | IS3 | Lactobacillus sake | 2230 | 0 |
| LMGH100 | IS1310 | IS256 |  | Enterococcus hirae | 2157 | 0 |
| LMGH100 | IS1070 | IS30 |  | Leuconostoc lactis | 1925 | 0 |
| LMGH100 | ISLhe30 | IS30 |  | Lactobacillus helveticus | 1566 | 0 |
| LMGH100 | ISWci2 | IS3 | IS3 | Weissella cibaria | 1073 | 0 |
| LMGH100 | ISLhe11 | ISLre2 |  | Lactobacillus helveticus | 763 | 0 |
| LMGH100 | ISLhe10 | ISLre2 |  | Lactobacillus helveticus | 519 | 5E-144 |
| LMGH100 | ISLhe13 | ISLre2 |  | Lactobacillus helveticus | 456 | 7E-125 |
| LMGH100 | ISLke1 | ISLre2 |  | Lactobacillus kefiranofaciens | 440 | 4E-120 |
| LMGH100 | ISLcr1 | ISLre2 |  | Lactobacillus crispatus | 387 | 5E-104 |
| LMGH100 | ISS1A | IS6 |  | Lactococcus lactis | 230 | 7E-57 |
| LMGH100 | ISS1E | IS6 |  | Lactococcus lactis | 226 | 1E-55 |
| LMGH100 | ISS1N | IS6 |  | Lactococcus lactis | 222 | 2E-54 |
| LMGH100 | IS1297 | IS6 |  | Leuconostoc mesenteroides | 222 | 2E-54 |
| LMGH100 | ISS1D | IS6 |  | Lactococcus lactis | 214 | 4E-52 |
| LMGH100 | ISS1M | IS6 |  | Lactococcus lactis | 204 | 4E-49 |
| LMGH100 | ISS1CH | IS6 |  | Lactococcus lactis | 204 | 4E-49 |
| LMGH100 | ISLcr2 | ISLre2 |  | Lactobacillus crispatus | 137 | 8E-29 |
| LMGH100 | ISLde1 | ISLre2 |  | Lactobacillus delbrueckii | 135 | 3E-28 |
| LMGH100 | ISS1X | IS6 |  | Lactococcus lactis | 89.7 | 2E-14 |
| LMGH100 | ISS1T | IS6 |  | Lactococcus lactis | 89.7 | 2E-14 |
| LMGH100 | ISS1S | IS6 |  | Lactococcus lactis | 89.7 | 2E-14 |
| LMGH100 | ISS1RS | IS6 |  | Lactococcus lactis | 89.7 | 2E-14 |
| LMGH100 | IS946V | IS6 |  | Lactococcus lactis | 87.7 | 7E-14 |
| LMGH100 | ISS1B | IS6 |  | Lactococcus lactis | 81.8 | 4E-12 |
| LMGH100 | ISS1Z | IS6 |  | Lactococcus lactis | 67.9 | 0.00000006 |
| LMGH100 | ISLmo16 | IS3 | IS150 | Listeria monocytogenes | 62.0 | 0.000004 |
| LMGH100 | ISTeha2 | IS6 |  | Tetragenococcus halophilus | 60.0 | 0.00002 |
| LMGH100 | ISBwe2 | IS6 |  | Bacillus weihenstephanensis | 56.0 | 0.0002 |
| LMGH100 | ISBwe3 | IS6 |  | Bacillus weihenstephanensis | 54.0 | 0.001 |
| LMGH100 | ISLmo4 | IS6 |  | Listeria monocytogenes | 52.0 | 0.004 |
| LMGH278 | ISLhe30 | IS30 |  | Lactobacillus helveticus | 1566 | 0 |
| LMGH278 | IS1070 | IS30 |  | Leuconostoc lactis | 1164 | 0 |
| LMGH278 | ISWci2 | IS3 | IS3 | Weissella cibaria | 654 | 0 |
| LMGH278 | ISLpl1 | IS30 |  | Lactobacillus plantarum | 232 | 2E-57 |
| LMGH278 | ISPp1 | IS30 |  | Pediococcus pentosaceus | 184 | 4E-43 |
| LMGH278 | ISLmo16 | IS3 | IS150 | Listeria monocytogenes | 61.9 | 0.000004 |
| LMGH280 | IS1070 | IS30 |  | Leuconostoc lactis | 1925 | 0 |
| LMGH280 | ISLhe30 | IS30 |  | Lactobacillus helveticus | 1566 | 0 |
| LMGH280 | ISWci2 | IS3 | IS3 | Weissella cibaria | 1057 | 0 |
| LMGH280 | ISLpl1 | IS30 |  | Lactobacillus plantarum | 232 | 2E-57 |
| LMGH280 | IS1165 | ISL3 |  | Leuconostoc mesenteroides | 228 | 3E-56 |
| LMGH280 | ISPp1 | IS30 |  | Pediococcus pentosaceus | 184 | 4E-43 |
| LMGH280 | ISLmo16 | IS3 | IS150 | Listeria monocytogenes | 61.9 | 0.000004 |
| LMGH284 | IS1163 | IS3 | IS3 | Lactobacillus sake | 2230 | 0 |
| LMGH284 | IS1310 | IS256 |  | Enterococcus hirae | 2157 | 0 |
| LMGH284 | IS1070 | IS30 |  | Leuconostoc lactis | 1933 | 0 |
| LMGH284 | ISLhe30 | IS30 |  | Lactobacillus helveticus | 1566 | 0 |
| LMGH284 | ISWci2 | IS3 | IS3 | Weissella cibaria | 1072 | 0 |
| LMGH284 | ISLhe11 | ISLre2 |  | Lactobacillus helveticus | 763 | 0 |
| LMGH284 | ISLhe10 | ISLre2 |  | Lactobacillus helveticus | 519 | 6E-144 |
| LMGH284 | ISLhe13 | ISLre2 |  | Lactobacillus helveticus | 456 | 7E-125 |
| LMGH284 | ISLke1 | ISLre2 |  | Lactobacillus kefiranofaciens | 440 | 4E-120 |
| LMGH284 | ISLcr1 | ISLre2 |  | Lactobacillus crispatus | 387 | 5E-104 |
| LMGH284 | ISLcr2 | ISLre2 |  | Lactobacillus crispatus | 137 | 8E-29 |
| LMGH284 | ISLde1 | ISLre2 |  | Lactobacillus delbrueckii | 135 | 3E-28 |
| LMGH284 | ISLmo16 | IS3 | IS150 | Listeria monocytogenes | 61.9 | 0.000004 |
| LMGH284 | ISBwe2 | IS6 |  | Bacillus weihenstephanensis | 56.0 | 0.0002 |
| LMGH284 | ISBwe3 | IS6 |  | Bacillus weihenstephanensis | 54.0 | 0.001 |
| LMGH61 | IS1070 | IS30 |  | Leuconostoc lactis | 1925 | 0 |
| LMGH61 | ISLhe30 | IS30 |  | Lactobacillus helveticus | 1566 | 0 |
| LMGH61 | ISWci2 | IS3 | IS3 | Weissella cibaria | 1057 | 0 |
| LMGH61 | ISLpl1 | IS30 |  | Lactobacillus plantarum | 232 | 2E-57 |
| LMGH61 | IS1165 | ISL3 |  | Leuconostoc mesenteroides | 228 | 3E-56 |
| LMGH61 | ISPp1 | IS30 |  | Pediococcus pentosaceus | 184 | 4E-43 |
| LMGH61 | ISLmo16 | IS3 | IS150 | Listeria monocytogenes | 61.9 | 0.000004 |
| LMGH83 | IS1163 | IS3 | IS3 | Lactobacillus sake | 2230 | 0 |
| LMGH83 | IS1310 | IS256 |  | Enterococcus hirae | 2157 | 0 |
| LMGH83 | IS1070 | IS30 |  | Leuconostoc lactis | 1933 | 0 |
| LMGH83 | ISLhe30 | IS30 |  | Lactobacillus helveticus | 1566 | 0 |
| LMGH83 | ISWci2 | IS3 | IS3 | Weissella cibaria | 1073 | 0 |
| LMGH83 | ISLhe11 | ISLre2 |  | Lactobacillus helveticus | 763 | 0 |
| LMGH83 | ISLhe10 | ISLre2 |  | Lactobacillus helveticus | 519 | 5E-144 |
| LMGH83 | ISLhe13 | ISLre2 |  | Lactobacillus helveticus | 456 | 7E-125 |
| LMGH83 | ISLke1 | ISLre2 |  | Lactobacillus kefiranofaciens | 440 | 4E-120 |
| LMGH83 | ISLcr1 | ISLre2 |  | Lactobacillus crispatus | 387 | 5E-104 |
| LMGH83 | ISS1A | IS6 |  | Lactococcus lactis | 230 | 7E-57 |
| LMGH83 | ISS1E | IS6 |  | Lactococcus lactis | 226 | 1E-55 |
| LMGH83 | ISS1N | IS6 |  | Lactococcus lactis | 222 | 2E-54 |
| LMGH83 | IS1297 | IS6 |  | Leuconostoc mesenteroides | 222 | 2E-54 |
| LMGH83 | ISS1D | IS6 |  | Lactococcus lactis | 214 | 4E-52 |
| LMGH83 | ISS1M | IS6 |  | Lactococcus lactis | 204 | 4E-49 |
| LMGH83 | ISS1CH | IS6 |  | Lactococcus lactis | 204 | 4E-49 |
| LMGH83 | ISLcr2 | ISLre2 |  | Lactobacillus crispatus | 137 | 8E-29 |
| LMGH83 | ISLde1 | ISLre2 |  | Lactobacillus delbrueckii | 135 | 3E-28 |
| LMGH83 | ISS1X | IS6 |  | Lactococcus lactis | 89.7 | 2E-14 |
| LMGH83 | ISS1T | IS6 |  | Lactococcus lactis | 89.7 | 2E-14 |
| LMGH83 | ISS1S | IS6 |  | Lactococcus lactis | 89.7 | 2E-14 |
| LMGH83 | ISS1RS | IS6 |  | Lactococcus lactis | 89.7 | 2E-14 |
| LMGH83 | IS946V | IS6 |  | Lactococcus lactis | 87.7 | 7E-14 |
| LMGH83 | ISS1B | IS6 |  | Lactococcus lactis | 81.8 | 4E-12 |
| LMGH83 | ISS1Z | IS6 |  | Lactococcus lactis | 67.9 | 0.00000006 |
| LMGH83 | ISLmo16 | IS3 | IS150 | Listeria monocytogenes | 62.0 | 0.000004 |
| LMGH83 | ISTeha2 | IS6 |  | Tetragenococcus halophilus | 60.0 | 0.00002 |
| LMGH83 | ISBwe2 | IS6 |  | Bacillus weihenstephanensis | 56.0 | 0.0002 |
| LMGH83 | ISBwe3 | IS6 |  | Bacillus weihenstephanensis | 54.0 | 0.001 |
| LMGH83 | ISLmo4 | IS6 |  | Listeria monocytogenes | 52.0 | 0.004 |
| LMGH95 | IS1163 | IS3 | IS3 | Lactobacillus sake | 2230 | 0 |
| LMGH95 | IS1310 | IS256 |  | Enterococcus hirae | 2157 | 0 |
| LMGH95 | ISLhe30 | IS30 |  | Lactobacillus helveticus | 1566 | 0 |
| LMGH95 | ISWci2 | IS3 | IS3 | Weissella cibaria | 1072 | 0 |
| LMGH95 | ISLhe11 | ISLre2 |  | Lactobacillus helveticus | 763 | 0 |
| LMGH95 | IS1070 | IS30 |  | Leuconostoc lactis | 607 | 3E-170 |
| LMGH95 | ISLhe10 | ISLre2 |  | Lactobacillus helveticus | 519 | 5E-144 |
| LMGH95 | ISLhe13 | ISLre2 |  | Lactobacillus helveticus | 456 | 7E-125 |
| LMGH95 | ISLke1 | ISLre2 |  | Lactobacillus kefiranofaciens | 440 | 4E-120 |
| LMGH95 | ISLcr1 | ISLre2 |  | Lactobacillus crispatus | 387 | 5E-104 |
| LMGH95 | ISS1A | IS6 |  | Lactococcus lactis | 230 | 7E-57 |
| LMGH95 | ISS1E | IS6 |  | Lactococcus lactis | 226 | 1E-55 |
| LMGH95 | ISS1N | IS6 |  | Lactococcus lactis | 222 | 2E-54 |
| LMGH95 | IS1297 | IS6 |  | Leuconostoc mesenteroides | 222 | 2E-54 |
| LMGH95 | ISS1D | IS6 |  | Lactococcus lactis | 214 | 4E-52 |
| LMGH95 | ISS1M | IS6 |  | Lactococcus lactis | 204 | 4E-49 |
| LMGH95 | ISS1CH | IS6 |  | Lactococcus lactis | 204 | 4E-49 |
| LMGH95 | ISLcr2 | ISLre2 |  | Lactobacillus crispatus | 137 | 8E-29 |
| LMGH95 | ISLde1 | ISLre2 |  | Lactobacillus delbrueckii | 135 | 3E-28 |
| LMGH95 | ISS1X | IS6 |  | Lactococcus lactis | 89.7 | 2E-14 |
| LMGH95 | ISS1T | IS6 |  | Lactococcus lactis | 89.7 | 2E-14 |
| LMGH95 | ISS1S | IS6 |  | Lactococcus lactis | 89.7 | 2E-14 |
| LMGH95 | ISS1RS | IS6 |  | Lactococcus lactis | 89.7 | 2E-14 |
| LMGH95 | IS946V | IS6 |  | Lactococcus lactis | 87.7 | 7E-14 |
| LMGH95 | ISS1B | IS6 |  | Lactococcus lactis | 81.8 | 4E-12 |
| LMGH95 | ISS1Z | IS6 |  | Lactococcus lactis | 67.9 | 0.00000006 |
| LMGH95 | ISLmo16 | IS3 | IS150 | Listeria monocytogenes | 61.9 | 0.000004 |
| LMGH95 | ISTeha2 | IS6 |  | Tetragenococcus halophilus | 60.0 | 0.00001 |
| LMGH95 | ISBwe2 | IS6 |  | Bacillus weihenstephanensis | 56.0 | 0.0002 |
| LMGH95 | ISBwe3 | IS6 |  | Bacillus weihenstephanensis | 54.0 | 0.001 |
| LMGH95 | ISLmo4 | IS6 |  | Listeria monocytogenes | 52.0 | 0.004 |
| LMGH97 | ISLhe30 | IS30 |  | Lactobacillus helveticus | 1558 | 0 |
| LMGH97 | IS1070 | IS30 |  | Leuconostoc lactis | 1275 | 0 |
| LMGH97 | ISLhe11 | ISLre2 |  | Lactobacillus helveticus | 763 | 0 |
| LMGH97 | ISWci2 | IS3 | IS3 | Weissella cibaria | 543 | 4E-151 |
| LMGH97 | ISLhe10 | ISLre2 |  | Lactobacillus helveticus | 519 | 5E-144 |
| LMGH97 | ISLhe13 | ISLre2 |  | Lactobacillus helveticus | 456 | 7E-125 |
| LMGH97 | ISLke1 | ISLre2 |  | Lactobacillus kefiranofaciens | 440 | 4E-120 |
| LMGH97 | ISLcr1 | ISLre2 |  | Lactobacillus crispatus | 387 | 5E-104 |
| LMGH97 | ISLpl1 | IS30 |  | Lactobacillus plantarum | 232 | 2E-57 |
| LMGH97 | ISPp1 | IS30 |  | Pediococcus pentosaceus | 184 | 4E-43 |
| LMGH97 | ISLcr2 | ISLre2 |  | Lactobacillus crispatus | 137 | 8E-29 |
| LMGH97 | ISLde1 | ISLre2 |  | Lactobacillus delbrueckii | 135 | 3E-28 |
| LMGH97 | ISLmo16 | IS3 | IS150 | Listeria monocytogenes | 61.9 | 0.000004 |
| LMGH97 | ISBwe2 | IS6 |  | Bacillus weihenstephanensis | 56.0 | 0.0002 |
| LMGH97 | ISBwe3 | IS6 |  | Bacillus weihenstephanensis | 54.0 | 0.001 |
| LMGTW1 | ISLhe30 | IS30 |  | Lactobacillus helveticus | 1558 | 0 |
| LMGTW1 | IS1070 | IS30 |  | Leuconostoc lactis | 1275 | 0 |
| LMGTW1 | ISWci2 | IS3 | IS3 | Weissella cibaria | 543 | 4E-151 |
| LMGTW1 | ISLpl1 | IS30 |  | Lactobacillus plantarum | 232 | 2E-57 |
| LMGTW1 | ISPp1 | IS30 |  | Pediococcus pentosaceus | 184 | 4E-43 |
| LMGTW1 | ISLmo16 | IS3 | IS150 | Listeria monocytogenes | 61.9 | 0.000004 |
| LMGTW3 | IS1165 | ISL3 |  | Leuconostoc mesenteroides | 1798 | 0 |
| LMGTW3 | IS1297 | IS6 |  | Leuconostoc mesenteroides | 1602 | 0 |
| LMGTW3 | ISLhe30 | IS30 |  | Lactobacillus helveticus | 1566 | 0 |
| LMGTW3 | ISS1N | IS6 |  | Lactococcus lactis | 1515 | 0 |
| LMGTW3 | ISS1E | IS6 |  | Lactococcus lactis | 1511 | 0 |
| LMGTW3 | ISS1M | IS6 |  | Lactococcus lactis | 1489 | 0 |
| LMGTW3 | ISS1D | IS6 |  | Lactococcus lactis | 1475 | 0 |
| LMGTW3 | ISS1CH | IS6 |  | Lactococcus lactis | 1413 | 0 |
| LMGTW3 | IS1070 | IS30 |  | Leuconostoc lactis | 1082 | 0 |
| LMGTW3 | ISWci2 | IS3 | IS3 | Weissella cibaria | 1049 | 0 |
| LMGTW3 | ISS1A | IS6 |  | Lactococcus lactis | 882 | 0 |
| LMGTW3 | IS946V | IS6 |  | Lactococcus lactis | 858 | 0 |
| LMGTW3 | ISLhe11 | ISLre2 |  | Lactobacillus helveticus | 763 | 0 |
| LMGTW3 | ISS1T | IS6 |  | Lactococcus lactis | 652 | 0 |
| LMGTW3 | ISS1S | IS6 |  | Lactococcus lactis | 628 | 8E-177 |
| LMGTW3 | ISS1RS | IS6 |  | Lactococcus lactis | 628 | 8E-177 |
| LMGTW3 | ISS1B | IS6 |  | Lactococcus lactis | 628 | 8E-177 |
| LMGTW3 | ISS1X | IS6 |  | Lactococcus lactis | 620 | 2E-174 |
| LMGTW3 | ISLgar4 | IS6 |  | Lactococcus garvieae | 618 | 7E-174 |
| LMGTW3 | ISS1Z | IS6 |  | Lactococcus lactis | 573 | 4E-160 |
| LMGTW3 | ISLhe10 | ISLre2 |  | Lactobacillus helveticus | 505 | 8E-140 |
| LMGTW3 | ISLhe13 | ISLre2 |  | Lactobacillus helveticus | 442 | 1E-120 |
| LMGTW3 | ISLke1 | ISLre2 |  | Lactobacillus kefiranofaciens | 426 | 6E-116 |
| LMGTW3 | ISLcr1 | ISLre2 |  | Lactobacillus crispatus | 373 | 7E-100 |
| LMGTW3 | ISLpl1 | IS30 |  | Lactobacillus plantarum | 232 | 2E-57 |
| LMGTW3 | IS1216E | IS6 |  | Enterococcus faecium | 228 | 3E-56 |
| LMGTW3 | ISS1W | IS6 |  | Lactococcus lactis | 220 | 7E-54 |
| LMGTW3 | IS1216V | IS6 |  | Enterococcus sp. | 212 | 2E-51 |
| LMGTW3 | IS1216 | IS6 |  | Enterococcus hirae | 196 | 1E-46 |
| LMGTW3 | ISPp1 | IS30 |  | Pediococcus pentosaceus | 184 | 4E-43 |
| LMGTW3 | ISLmo19 | IS6 |  | Listeria monocytogenes | 149 | 2E-32 |
| LMGTW3 | ISTeha2 | IS6 |  | Tetragenococcus halophilus | 149 | 2E-32 |
| LMGTW3 | ISLmo14 | IS6 |  | Listeria monocytogenes | 137 | 8E-29 |
| LMGTW3 | ISLcr2 | ISLre2 |  | Lactobacillus crispatus | 137 | 8E-29 |
| LMGTW3 | ISLde1 | ISLre2 |  | Lactobacillus delbrueckii | 135 | 3E-28 |
| LMGTW3 | ISLmo13 | IS6 |  | Listeria monocytogenes | 133 | 1E-27 |
| LMGTW3 | ISEnfa1 | IS6 |  | Enterococcus faecium | 133 | 1E-27 |
| LMGTW3 | ISLmo4 | IS6 |  | Listeria monocytogenes | 81.8 | 4E-12 |
| LMGTW3 | ISLmo16 | IS3 | IS150 | Listeria monocytogenes | 61.9 | 0.000004 |
| LMGTW3 | ISLmo3 | IS6 |  | Listeria monocytogenes | 58.0 | 0.00006 |
| LMGTW3 | ISBwe2 | IS6 |  | Bacillus weihenstephanensis | 56.0 | 0.0002 |
| LMGTW3 | ISBwe3 | IS6 |  | Bacillus weihenstephanensis | 54.0 | 0.0009 |
| LMGTW6 | IS1165 | ISL3 |  | Leuconostoc mesenteroides | 1798 | 0 |
| LMGTW6 | IS1297 | IS6 |  | Leuconostoc mesenteroides | 1602 | 0 |
| LMGTW6 | ISLhe30 | IS30 |  | Lactobacillus helveticus | 1566 | 0 |
| LMGTW6 | ISS1N | IS6 |  | Lactococcus lactis | 1515 | 0 |
| LMGTW6 | ISS1E | IS6 |  | Lactococcus lactis | 1511 | 0 |
| LMGTW6 | ISS1M | IS6 |  | Lactococcus lactis | 1489 | 0 |
| LMGTW6 | ISS1D | IS6 |  | Lactococcus lactis | 1475 | 0 |
| LMGTW6 | ISS1CH | IS6 |  | Lactococcus lactis | 1413 | 0 |
| LMGTW6 | IS1070 | IS30 |  | Leuconostoc lactis | 1051 | 0 |
| LMGTW6 | ISS1A | IS6 |  | Lactococcus lactis | 882 | 0 |
| LMGTW6 | IS946V | IS6 |  | Lactococcus lactis | 858 | 0 |
| LMGTW6 | ISLhe11 | ISLre2 |  | Lactobacillus helveticus | 763 | 0 |
| LMGTW6 | ISWci2 | IS3 | IS3 | Weissella cibaria | 753 | 0 |
| LMGTW6 | ISS1T | IS6 |  | Lactococcus lactis | 652 | 0 |
| LMGTW6 | ISS1S | IS6 |  | Lactococcus lactis | 628 | 8E-177 |
| LMGTW6 | ISS1RS | IS6 |  | Lactococcus lactis | 628 | 8E-177 |
| LMGTW6 | ISS1B | IS6 |  | Lactococcus lactis | 628 | 8E-177 |
| LMGTW6 | ISS1X | IS6 |  | Lactococcus lactis | 620 | 2E-174 |
| LMGTW6 | ISLgar4 | IS6 |  | Lactococcus garvieae | 618 | 7E-174 |
| LMGTW6 | ISS1Z | IS6 |  | Lactococcus lactis | 573 | 4E-160 |
| LMGTW6 | ISLhe10 | ISLre2 |  | Lactobacillus helveticus | 505 | 8E-140 |
| LMGTW6 | ISLhe13 | ISLre2 |  | Lactobacillus helveticus | 442 | 1E-120 |
| LMGTW6 | ISLke1 | ISLre2 |  | Lactobacillus kefiranofaciens | 426 | 6E-116 |
| LMGTW6 | ISLcr1 | ISLre2 |  | Lactobacillus crispatus | 373 | 7E-100 |
| LMGTW6 | ISLpl1 | IS30 |  | Lactobacillus plantarum | 232 | 2E-57 |
| LMGTW6 | IS1216E | IS6 |  | Enterococcus faecium | 228 | 3E-56 |
| LMGTW6 | ISS1W | IS6 |  | Lactococcus lactis | 220 | 7E-54 |
| LMGTW6 | IS1216V | IS6 |  | Enterococcus sp. | 212 | 2E-51 |
| LMGTW6 | IS1216 | IS6 |  | Enterococcus hirae | 196 | 9E-47 |
| LMGTW6 | ISPp1 | IS30 |  | Pediococcus pentosaceus | 184 | 4E-43 |
| LMGTW6 | ISLmo19 | IS6 |  | Listeria monocytogenes | 149 | 2E-32 |
| LMGTW6 | ISTeha2 | IS6 |  | Tetragenococcus halophilus | 149 | 2E-32 |
| LMGTW6 | ISLmo14 | IS6 |  | Listeria monocytogenes | 137 | 8E-29 |
| LMGTW6 | ISLcr2 | ISLre2 |  | Lactobacillus crispatus | 137 | 8E-29 |
| LMGTW6 | ISLde1 | ISLre2 |  | Lactobacillus delbrueckii | 135 | 3E-28 |
| LMGTW6 | ISLmo13 | IS6 |  | Listeria monocytogenes | 133 | 1E-27 |
| LMGTW6 | ISEnfa1 | IS6 |  | Enterococcus faecium | 133 | 1E-27 |
| LMGTW6 | ISLmo4 | IS6 |  | Listeria monocytogenes | 81.8 | 4E-12 |
| LMGTW6 | ISLmo16 | IS3 | IS150 | Listeria monocytogenes | 61.9 | 0.000004 |
| LMGTW6 | ISLmo3 | IS6 |  | Listeria monocytogenes | 58.0 | 0.00006 |
| LMGTW6 | ISBwe2 | IS6 |  | Bacillus weihenstephanensis | 56.0 | 0.0002 |
| LMGTW6 | ISBwe3 | IS6 |  | Bacillus weihenstephanensis | 54.0 | 0.0009 |
| LMGTW8 | ISLhe30 | IS30 |  | Lactobacillus helveticus | 1558 | 0 |
| LMGTW8 | IS1070 | IS30 |  | Leuconostoc lactis | 1275 | 0 |
| LMGTW8 | ISLhe11 | ISLre2 |  | Lactobacillus helveticus | 763 | 0 |
| LMGTW8 | ISWci2 | IS3 | IS3 | Weissella cibaria | 543 | 4E-151 |
| LMGTW8 | ISLhe10 | ISLre2 |  | Lactobacillus helveticus | 519 | 5E-144 |
| LMGTW8 | ISLhe13 | ISLre2 |  | Lactobacillus helveticus | 456 | 7E-125 |
| LMGTW8 | ISLke1 | ISLre2 |  | Lactobacillus kefiranofaciens | 440 | 4E-120 |
| LMGTW8 | ISLcr1 | ISLre2 |  | Lactobacillus crispatus | 387 | 5E-104 |
| LMGTW8 | ISLpl1 | IS30 |  | Lactobacillus plantarum | 232 | 2E-57 |
| LMGTW8 | ISPp1 | IS30 |  | Pediococcus pentosaceus | 184 | 4E-43 |
| LMGTW8 | ISLcr2 | ISLre2 |  | Lactobacillus crispatus | 137 | 8E-29 |
| LMGTW8 | ISLde1 | ISLre2 |  | Lactobacillus delbrueckii | 135 | 3E-28 |
| LMGTW8 | ISLmo16 | IS3 | IS150 | Listeria monocytogenes | 61.9 | 0.000004 |
| LMGTW8 | ISBwe2 | IS6 |  | Bacillus weihenstephanensis | 56.0 | 0.0002 |
| LMGTW8 | ISBwe3 | IS6 |  | Bacillus weihenstephanensis | 54.0 | 0.001 |
| LN02 | IS1070 | IS30 |  | Leuconostoc lactis | 1933 | 0 |
| LN02 | IS1165 | ISL3 |  | Leuconostoc mesenteroides | 1798 | 0 |
| LN02 | IS1297 | IS6 |  | Leuconostoc mesenteroides | 1602 | 0 |
| LN02 | ISLhe30 | IS30 |  | Lactobacillus helveticus | 1566 | 0 |
| LN02 | ISS1N | IS6 |  | Lactococcus lactis | 1515 | 0 |
| LN02 | ISS1E | IS6 |  | Lactococcus lactis | 1511 | 0 |
| LN02 | ISS1M | IS6 |  | Lactococcus lactis | 1489 | 0 |
| LN02 | ISS1D | IS6 |  | Lactococcus lactis | 1475 | 0 |
| LN02 | ISS1CH | IS6 |  | Lactococcus lactis | 1413 | 0 |
| LN02 | ISS1A | IS6 |  | Lactococcus lactis | 882 | 0 |
| LN02 | IS946V | IS6 |  | Lactococcus lactis | 858 | 0 |
| LN02 | ISLhe11 | ISLre2 |  | Lactobacillus helveticus | 763 | 0 |
| LN02 | ISWci2 | IS3 | IS3 | Weissella cibaria | 753 | 0 |
| LN02 | ISS1T | IS6 |  | Lactococcus lactis | 652 | 0 |
| LN02 | ISS1S | IS6 |  | Lactococcus lactis | 628 | 8E-177 |
| LN02 | ISS1RS | IS6 |  | Lactococcus lactis | 628 | 8E-177 |
| LN02 | ISS1B | IS6 |  | Lactococcus lactis | 628 | 8E-177 |
| LN02 | ISS1X | IS6 |  | Lactococcus lactis | 620 | 2E-174 |
| LN02 | ISLgar4 | IS6 |  | Lactococcus garvieae | 618 | 8E-174 |
| LN02 | ISS1Z | IS6 |  | Lactococcus lactis | 573 | 4E-160 |
| LN02 | ISLhe10 | ISLre2 |  | Lactobacillus helveticus | 505 | 8E-140 |
| LN02 | ISLhe13 | ISLre2 |  | Lactobacillus helveticus | 442 | 1E-120 |
| LN02 | ISLke1 | ISLre2 |  | Lactobacillus kefiranofaciens | 426 | 6E-116 |
| LN02 | ISLcr1 | ISLre2 |  | Lactobacillus crispatus | 373 | 7E-100 |
| LN02 | ISLpl1 | IS30 |  | Lactobacillus plantarum | 232 | 2E-57 |
| LN02 | IS1216E | IS6 |  | Enterococcus faecium | 228 | 3E-56 |
| LN02 | ISS1W | IS6 |  | Lactococcus lactis | 220 | 7E-54 |
| LN02 | IS1216V | IS6 |  | Enterococcus sp. | 212 | 2E-51 |
| LN02 | IS1216 | IS6 |  | Enterococcus hirae | 196 | 1E-46 |
| LN02 | ISPp1 | IS30 |  | Pediococcus pentosaceus | 184 | 4E-43 |
| LN02 | ISLmo19 | IS6 |  | Listeria monocytogenes | 149 | 2E-32 |
| LN02 | ISTeha2 | IS6 |  | Tetragenococcus halophilus | 149 | 2E-32 |
| LN02 | ISLmo14 | IS6 |  | Listeria monocytogenes | 137 | 8E-29 |
| LN02 | ISLcr2 | ISLre2 |  | Lactobacillus crispatus | 137 | 8E-29 |
| LN02 | ISLde1 | ISLre2 |  | Lactobacillus delbrueckii | 135 | 3E-28 |
| LN02 | ISLmo13 | IS6 |  | Listeria monocytogenes | 133 | 1E-27 |
| LN02 | ISEnfa1 | IS6 |  | Enterococcus faecium | 133 | 1E-27 |
| LN02 | ISLmo4 | IS6 |  | Listeria monocytogenes | 81.8 | 4E-12 |
| LN02 | ISLmo16 | IS3 | IS150 | Listeria monocytogenes | 61.9 | 0.000004 |
| LN02 | ISLmo3 | IS6 |  | Listeria monocytogenes | 58.0 | 0.00006 |
| LN02 | ISBwe2 | IS6 |  | Bacillus weihenstephanensis | 56.0 | 0.0002 |
| LN02 | ISBwe3 | IS6 |  | Bacillus weihenstephanensis | 54.0 | 0.0009 |
| LN12 | IS1070 | IS30 |  | Leuconostoc lactis | 1865 | 0 |
| LN12 | IS1165 | ISL3 |  | Leuconostoc mesenteroides | 1798 | 0 |
| LN12 | IS1297 | IS6 |  | Leuconostoc mesenteroides | 1602 | 0 |
| LN12 | ISLhe30 | IS30 |  | Lactobacillus helveticus | 1566 | 0 |
| LN12 | ISS1N | IS6 |  | Lactococcus lactis | 1515 | 0 |
| LN12 | ISS1E | IS6 |  | Lactococcus lactis | 1511 | 0 |
| LN12 | ISS1M | IS6 |  | Lactococcus lactis | 1489 | 0 |
| LN12 | ISS1D | IS6 |  | Lactococcus lactis | 1475 | 0 |
| LN12 | ISS1CH | IS6 |  | Lactococcus lactis | 1413 | 0 |
| LN12 | ISWci2 | IS3 | IS3 | Weissella cibaria | 1049 | 0 |
| LN12 | ISS1A | IS6 |  | Lactococcus lactis | 882 | 0 |
| LN12 | IS946V | IS6 |  | Lactococcus lactis | 858 | 0 |
| LN12 | ISS1T | IS6 |  | Lactococcus lactis | 652 | 0 |
| LN12 | ISS1S | IS6 |  | Lactococcus lactis | 628 | 8E-177 |
| LN12 | ISS1RS | IS6 |  | Lactococcus lactis | 628 | 8E-177 |
| LN12 | ISS1B | IS6 |  | Lactococcus lactis | 628 | 8E-177 |
| LN12 | ISS1X | IS6 |  | Lactococcus lactis | 620 | 2E-174 |
| LN12 | ISLgar4 | IS6 |  | Lactococcus garvieae | 618 | 7E-174 |
| LN12 | ISS1Z | IS6 |  | Lactococcus lactis | 573 | 4E-160 |
| LN12 | ISLpl1 | IS30 |  | Lactobacillus plantarum | 232 | 2E-57 |
| LN12 | IS1216E | IS6 |  | Enterococcus faecium | 228 | 3E-56 |
| LN12 | ISS1W | IS6 |  | Lactococcus lactis | 220 | 6E-54 |
| LN12 | IS1216V | IS6 |  | Enterococcus sp. | 212 | 2E-51 |
| LN12 | IS1216 | IS6 |  | Enterococcus hirae | 196 | 9E-47 |
| LN12 | ISPp1 | IS30 |  | Pediococcus pentosaceus | 184 | 4E-43 |
| LN12 | ISLmo19 | IS6 |  | Listeria monocytogenes | 149 | 2E-32 |
| LN12 | ISTeha2 | IS6 |  | Tetragenococcus halophilus | 149 | 2E-32 |
| LN12 | ISLmo14 | IS6 |  | Listeria monocytogenes | 137 | 7E-29 |
| LN12 | ISLmo13 | IS6 |  | Listeria monocytogenes | 133 | 1E-27 |
| LN12 | ISEnfa1 | IS6 |  | Enterococcus faecium | 133 | 1E-27 |
| LN12 | ISLmo4 | IS6 |  | Listeria monocytogenes | 81.8 | 4E-12 |
| LN12 | ISLmo16 | IS3 | IS150 | Listeria monocytogenes | 61.9 | 0.000004 |
| LN12 | ISLmo3 | IS6 |  | Listeria monocytogenes | 58.0 | 0.00006 |
| LN23 | IS1163 | IS3 | IS3 | Lactobacillus sake | 2230 | 0 |
| LN23 | IS1310 | IS256 |  | Enterococcus hirae | 2157 | 0 |
| LN23 | IS1070 | IS30 |  | Leuconostoc lactis | 1923 | 0 |
| LN23 | ISWci2 | IS3 | IS3 | Weissella cibaria | 1072 | 0 |
| LN23 | ISLhe11 | ISLre2 |  | Lactobacillus helveticus | 763 | 0 |
| LN23 | ISLhe10 | ISLre2 |  | Lactobacillus helveticus | 519 | 5E-144 |
| LN23 | ISLhe13 | ISLre2 |  | Lactobacillus helveticus | 456 | 7E-125 |
| LN23 | ISLke1 | ISLre2 |  | Lactobacillus kefiranofaciens | 440 | 4E-120 |
| LN23 | ISLcr1 | ISLre2 |  | Lactobacillus crispatus | 387 | 5E-104 |
| LN23 | ISLhe30 | IS30 |  | Lactobacillus helveticus | 242 | 2E-60 |
| LN23 | ISLcr2 | ISLre2 |  | Lactobacillus crispatus | 137 | 8E-29 |
| LN23 | ISLde1 | ISLre2 |  | Lactobacillus delbrueckii | 135 | 3E-28 |
| LN23 | ISLmo16 | IS3 | IS150 | Listeria monocytogenes | 61.9 | 0.000004 |
| LN23 | ISBwe2 | IS6 |  | Bacillus weihenstephanensis | 56.0 | 0.0002 |
| LN23 | ISBwe3 | IS6 |  | Bacillus weihenstephanensis | 54.0 | 0.001 |
| MGBC116435 | IS1070 | IS30 |  | Leuconostoc lactis | 1877 | 0 |
| MGBC116435 | ISLgar4 | IS6 |  | Lactococcus garvieae | 1394 | 0 |
| MGBC116435 | ISWci2 | IS3 | IS3 | Weissella cibaria | 1013 | 0 |
| MGBC116435 | IS1310 | IS256 |  | Enterococcus hirae | 860 | 0 |
| MGBC116435 | ISLmo19 | IS6 |  | Listeria monocytogenes | 846 | 0 |
| MGBC116435 | IS1216E | IS6 |  | Enterococcus faecium | 841 | 0 |
| MGBC116435 | IS1216V | IS6 |  | Enterococcus sp. | 825 | 0 |
| MGBC116435 | IS1216 | IS6 |  | Enterococcus hirae | 771 | 0 |
| MGBC116435 | IS946V | IS6 |  | Lactococcus lactis | 644 | 0 |
| MGBC116435 | IS1297 | IS6 |  | Leuconostoc mesenteroides | 618 | 8E-174 |
| MGBC116435 | ISS1N | IS6 |  | Lactococcus lactis | 591 | 2E-165 |
| MGBC116435 | ISS1E | IS6 |  | Lactococcus lactis | 587 | 3E-164 |
| MGBC116435 | ISS1M | IS6 |  | Lactococcus lactis | 581 | 2E-162 |
| MGBC116435 | ISS1D | IS6 |  | Lactococcus lactis | 579 | 7E-162 |
| MGBC116435 | ISS1W | IS6 |  | Lactococcus lactis | 571 | 2E-159 |
| MGBC116435 | ISS1CH | IS6 |  | Lactococcus lactis | 571 | 2E-159 |
| MGBC116435 | ISS1X | IS6 |  | Lactococcus lactis | 565 | 1E-157 |
| MGBC116435 | ISS1S | IS6 |  | Lactococcus lactis | 565 | 1E-157 |
| MGBC116435 | ISS1B | IS6 |  | Lactococcus lactis | 557 | 3E-155 |
| MGBC116435 | ISS1T | IS6 |  | Lactococcus lactis | 549 | 6E-153 |
| MGBC116435 | ISS1RS | IS6 |  | Lactococcus lactis | 549 | 6E-153 |
| MGBC116435 | ISS1A | IS6 |  | Lactococcus lactis | 547 | 3E-152 |
| MGBC116435 | ISS1Z | IS6 |  | Lactococcus lactis | 533 | 4E-148 |
| MGBC116435 | ISLmo13 | IS6 |  | Listeria monocytogenes | 442 | 1E-120 |
| MGBC116435 | ISTeha2 | IS6 |  | Tetragenococcus halophilus | 244 | 5E-61 |
| MGBC116435 | ISEnfa1 | IS6 |  | Enterococcus faecium | 226 | 1E-55 |
| MGBC116435 | ISLmo4 | IS6 |  | Listeria monocytogenes | 216 | 1E-52 |
| MGBC116435 | ISLmo14 | IS6 |  | Listeria monocytogenes | 192 | 2E-45 |
| MGBC116435 | IS1076 | IS3 | IS3 | Lactococcus lactis | 77.8 | 7E-11 |
| MGBC116435 | IS1069 | IS3 | IS3 | Lactococcus lactis | 77.8 | 7E-11 |
| MGBC116435 | IS1068 | IS3 | IS3 | Lactococcus lactis | 77.8 | 7E-11 |
| MGBC116435 | ISLmo16 | IS3 | IS150 | Listeria monocytogenes | 61.9 | 0.000004 |
| MGBC116435 | ISLla3 | IS3 | IS150 | Lactococcus lactis | 58.0 | 0.00006 |
| MGBC116435 | IS240A | IS6 |  | Bacillus thuringiensis | 52.0 | 0.004 |
| NCDO_768 | IS1070 | IS30 |  | Leuconostoc lactis | 180 | 6E-42 |
| PS12 | ISLhe30 | IS30 |  | Lactobacillus helveticus | 1550 | 0 |
| PS12 | IS1070 | IS30 |  | Leuconostoc lactis | 1292 | 0 |
| PS12 | ISWci2 | IS3 | IS3 | Weissella cibaria | 805 | 0 |
| PS12 | ISLhe11 | ISLre2 |  | Lactobacillus helveticus | 763 | 0 |
| PS12 | ISLhe10 | ISLre2 |  | Lactobacillus helveticus | 519 | 5E-144 |
| PS12 | ISLhe13 | ISLre2 |  | Lactobacillus helveticus | 456 | 7E-125 |
| PS12 | ISLke1 | ISLre2 |  | Lactobacillus kefiranofaciens | 440 | 4E-120 |
| PS12 | ISLcr1 | ISLre2 |  | Lactobacillus crispatus | 387 | 5E-104 |
| PS12 | ISLpl1 | IS30 |  | Lactobacillus plantarum | 232 | 2E-57 |
| PS12 | ISPp1 | IS30 |  | Pediococcus pentosaceus | 184 | 4E-43 |
| PS12 | ISLcr2 | ISLre2 |  | Lactobacillus crispatus | 137 | 8E-29 |
| PS12 | ISLde1 | ISLre2 |  | Lactobacillus delbrueckii | 135 | 3E-28 |
| PS12 | ISLmo16 | IS3 | IS150 | Listeria monocytogenes | 61.9 | 0.000004 |
| PS12 | ISBwe2 | IS6 |  | Bacillus weihenstephanensis | 56.0 | 0.0002 |
| PS12 | ISBwe3 | IS6 |  | Bacillus weihenstephanensis | 54.0 | 0.0009 |
| TMW21073 | IS1070 | IS30 |  | Leuconostoc lactis | 1941 | 0 |
| TMW21073 | IS1520 | IS3 | IS3 | Lactobacillus sakei | 71.9 | 5E-09 |
| TMW21073 | ISLmo21 | IS3 | IS3 | Listeria monocytogenes | 54.0 | 0.001 |
| TMW21073 | ISWci2 | IS3 | IS3 | Weissella cibaria | 54.0 | 0.001 |
| TMW21073 | IS712 | IS21 |  | Lactococcus lactis | 52.0 | 0.004 |
| TMW21195 | IS1070 | IS30 |  | Leuconostoc lactis | 1941 | 0 |
| TMW21195 | ISWci2 | IS3 | IS3 | Weissella cibaria | 1750 | 0 |
| TMW21195 | ISLhe30 | IS30 |  | Lactobacillus helveticus | 1542 | 0 |
| TMW21195 | Tn3 | Tn3 |  | Salmonella enterica | 264 | 5E-67 |
| TMW21195 | Tn2 | Tn3 |  | Escherichia coli | 256 | 1E-64 |
| TMW21195 | TnAs3 | Tn3 |  | Aeromonas salmonicida | 93.7 | 1E-15 |
| TMW21195 | IS712 | IS21 |  | Lactococcus lactis | 52.0 | 0.004 |
| TR070 | IS1070 | IS30 |  | Leuconostoc lactis | 1933 | 0 |
| TR070 | ISLpl1 | IS30 |  | Lactobacillus plantarum | 1707 | 0 |
| TR070 | ISPp1 | IS30 |  | Pediococcus pentosaceus | 1612 | 0 |
| TR070 | ISWci2 | IS3 | IS3 | Weissella cibaria | 377 | 6E-101 |
| UBA11295 | ISLhe30 | IS30 |  | Lactobacillus helveticus | 1558 | 0 |
| UBA11295 | ISLmo16 | IS3 | IS150 | Listeria monocytogenes | 61.9 | 0.000003 |

**Supplementary Table 4.** Number of horizontally transferred genes across forty *Ln. pseudomesenteroides* strains.

| Strain | Bacillus akibai JCM 9157 | Bacillus cellulosilyticus DSM 2522 | Bacillus massilioanorexius AP8 | Bacillus megaterium MSP20.1 | Bacillus niacini | Bacillus sp. JGI 001006-L10 | Bacillus sp. UNC438CL73TsuS30 | Desulfovibrio frigidus DSM 17176 | Facklamia hominis CCUG 36813 | Flavobacterium frigoris PS1 | Galbibacter marinus | Gracilibacillus boraciitolerans JCM 21714 | Lacticigenium naphtae DSM 19658 | Lewinella cohaerens DSM 23179 | Maribacter sp. HTCC2170 | Myroides [odoratimimus] CIP 103059 | Oceanobacillus manasiensis | Paenibacillus sp. URHA0014 | Psychroserpens burtonensis DSM 12212 | Salsuginibacillus kocurii DSM 18087 | Staphylococcus epidermidis | Staphylococcus epidermidis 14.1.R1.SE | Staphylococcus epidermidis 36-1 | Staphylococcus epidermidis APO35 | Staphylococcus epidermidis Scl19 | Staphylococcus epidermidis Scl22 | Staphylococcus epidermidis W23144 | Streptococcus criceti HS-6 | Streptococcus devriesei DSM 19639 | Virgibacillus alimentarius | Wolbachia sp. wRi | Yersinia enterocolitica (type O:9) str. YE56/03 | Yersinia pestis PY-03 |
| --- | --- | --- | --- | --- | --- | --- | --- | --- | --- | --- | --- | --- | --- | --- | --- | --- | --- | --- | --- | --- | --- | --- | --- | --- | --- | --- | --- | --- | --- | --- | --- | --- | --- |
| 1159 | 3 | 0 | 0 | 0 | 0 | 0 | 0 | 0 | 0 | 0 | 0 | 0 | 1 | 0 | 1 | 0 | 0 | 0 | 0 | 0 | 0 | 0 | 0 | 0 | 0 | 0 | 0 | 0 | 0 | 0 | 0 | 0 | 0 |
| 4882 | 1 | 0 | 0 | 0 | 0 | 0 | 0 | 0 | 1 | 0 | 0 | 0 | 0 | 0 | 0 | 0 | 0 | 0 | 0 | 0 | 0 | 0 | 0 | 0 | 0 | 0 | 0 | 0 | 0 | 0 | 0 | 0 | 0 |
| 17-2 | 2 | 0 | 0 | 0 | 0 | 0 | 0 | 0 | 0 | 0 | 0 | 1 | 0 | 0 | 0 | 0 | 0 | 0 | 4 | 0 | 0 | 0 | 0 | 0 | 0 | 0 | 0 | 0 | 0 | 0 | 0 | 0 | 0 |
| AMBR10 | 2 | 0 | 0 | 0 | 1 | 0 | 0 | 0 | 0 | 0 | 0 | 0 | 0 | 0 | 0 | 0 | 0 | 0 | 0 | 2 | 0 | 0 | 0 | 0 | 0 | 2 | 0 | 0 | 0 | 0 | 0 | 1 | 1 |
| BM_ | 2 | 0 | 0 | 0 | 0 | 0 | 0 | 0 | 0 | 0 | 0 | 0 | 0 | 0 | 0 | 0 | 0 | 0 | 0 | 0 | 0 | 0 | 0 | 0 | 0 | 0 | 0 | 0 | 0 | 0 | 0 | 0 | 0 |
| CBA3630 | 0 | 1 | 0 | 0 | 0 | 0 | 1 | 0 | 0 | 0 | 0 | 0 | 0 | 0 | 0 | 0 | 0 | 0 | 2 | 0 | 0 | 0 | 0 | 0 | 0 | 0 | 0 | 0 | 0 | 0 | 0 | 0 | 0 |
| Dm-9 | 1 | 0 | 0 | 0 | 0 | 0 | 0 | 0 | 0 | 0 | 0 | 0 | 0 | 0 | 0 | 0 | 0 | 0 | 1 | 0 | 1 | 1 | 0 | 0 | 1 | 0 | 0 | 0 | 0 | 1 | 0 | 0 | 0 |
| FDAARGOS_1003 | 0 | 0 | 0 | 0 | 0 | 0 | 0 | 0 | 0 | 0 | 0 | 0 | 0 | 1 | 0 | 0 | 0 | 0 | 2 | 0 | 1 | 0 | 0 | 0 | 0 | 0 | 0 | 0 | 0 | 0 | 0 | 0 | 0 |
| FDAARGOS_1004 | 2 | 0 | 0 | 0 | 0 | 0 | 0 | 0 | 1 | 0 | 0 | 0 | 0 | 1 | 0 | 0 | 0 | 1 | 0 | 0 | 0 | 0 | 1 | 0 | 0 | 0 | 0 | 0 | 0 | 0 | 0 | 0 | 0 |
| HPK01 | 0 | 0 | 0 | 0 | 0 | 0 | 0 | 0 | 0 | 2 | 0 | 0 | 0 | 0 | 0 | 0 | 0 | 0 | 0 | 0 | 0 | 0 | 0 | 0 | 0 | 0 | 0 | 0 | 0 | 0 | 0 | 0 | 0 |
| IM1374 | 4 | 0 | 0 | 0 | 0 | 0 | 0 | 0 | 0 | 0 | 0 | 0 | 0 | 0 | 0 | 1 | 0 | 0 | 1 | 0 | 0 | 0 | 0 | 0 | 0 | 0 | 0 | 0 | 0 | 0 | 0 | 0 | 0 |
| IM1427 | 2 | 0 | 0 | 0 | 0 | 0 | 0 | 0 | 0 | 0 | 0 | 0 | 0 | 0 | 0 | 0 | 0 | 0 | 0 | 0 | 1 | 0 | 0 | 0 | 0 | 0 | 0 | 0 | 0 | 1 | 0 | 0 | 0 |
| KCTC_3652 | 0 | 0 | 1 | 0 | 0 | 0 | 0 | 0 | 0 | 0 | 0 | 0 | 0 | 0 | 0 | 0 | 0 | 0 | 0 | 0 | 0 | 0 | 0 | 0 | 0 | 0 | 0 | 0 | 0 | 0 | 0 | 0 | 0 |
| KMB610 | 0 | 0 | 0 | 0 | 0 | 0 | 0 | 0 | 0 | 2 | 0 | 0 | 0 | 0 | 0 | 0 | 0 | 0 | 0 | 0 | 0 | 0 | 0 | 0 | 0 | 0 | 0 | 0 | 0 | 0 | 0 | 0 | 0 |
| LMG_11482 | 1 | 0 | 0 | 0 | 0 | 0 | 0 | 0 | 0 | 0 | 0 | 0 | 0 | 0 | 0 | 0 | 0 | 0 | 1 | 0 | 1 | 0 | 0 | 0 | 0 | 0 | 0 | 0 | 0 | 0 | 0 | 0 | 0 |
| LMG_11483 | 0 | 0 | 0 | 0 | 0 | 0 | 0 | 0 | 0 | 0 | 1 | 0 | 0 | 0 | 0 | 0 | 0 | 0 | 0 | 0 | 0 | 0 | 0 | 0 | 1 | 0 | 0 | 1 | 0 | 0 | 0 | 0 | 0 |
| LMGCF06 | 2 | 0 | 0 | 0 | 0 | 0 | 0 | 0 | 0 | 1 | 0 | 0 | 0 | 0 | 0 | 0 | 0 | 0 | 0 | 0 | 0 | 0 | 0 | 0 | 0 | 0 | 0 | 0 | 0 | 0 | 0 | 0 | 0 |
| LMGCF08 | 0 | 0 | 0 | 1 | 0 | 0 | 0 | 0 | 0 | 0 | 0 | 0 | 0 | 0 | 0 | 0 | 0 | 0 | 0 | 0 | 0 | 0 | 0 | 0 | 0 | 0 | 0 | 0 | 0 | 0 | 0 | 0 | 0 |
| LMGCF15 | 1 | 0 | 0 | 0 | 0 | 0 | 0 | 0 | 0 | 0 | 0 | 0 | 0 | 0 | 0 | 0 | 0 | 0 | 0 | 0 | 0 | 0 | 0 | 0 | 0 | 0 | 0 | 0 | 0 | 0 | 0 | 0 | 0 |
| LMGH100 | 0 | 0 | 0 | 0 | 0 | 1 | 0 | 0 | 0 | 0 | 0 | 0 | 0 | 0 | 0 | 0 | 0 | 0 | 0 | 0 | 0 | 0 | 0 | 0 | 0 | 0 | 0 | 0 | 0 | 0 | 0 | 0 | 0 |
| LMGH278 | 1 | 0 | 0 | 0 | 0 | 0 | 0 | 0 | 0 | 0 | 0 | 0 | 0 | 0 | 0 | 0 | 0 | 0 | 0 | 0 | 1 | 0 | 1 | 0 | 0 | 0 | 0 | 0 | 0 | 0 | 0 | 0 | 0 |
| LMGH280 | 2 | 0 | 0 | 0 | 0 | 0 | 0 | 0 | 0 | 0 | 0 | 0 | 0 | 0 | 0 | 0 | 1 | 0 | 0 | 0 | 0 | 0 | 0 | 0 | 0 | 0 | 0 | 0 | 1 | 0 | 0 | 0 | 0 |
| LMGH284 | 0 | 0 | 0 | 0 | 0 | 0 | 0 | 0 | 1 | 1 | 0 | 1 | 0 | 0 | 0 | 0 | 0 | 0 | 0 | 0 | 0 | 0 | 0 | 0 | 0 | 0 | 0 | 0 | 0 | 0 | 0 | 0 | 0 |
| LMGH61 | 1 | 0 | 0 | 0 | 0 | 0 | 0 | 0 | 0 | 1 | 0 | 0 | 0 | 0 | 0 | 0 | 0 | 0 | 0 | 0 | 0 | 0 | 0 | 0 | 0 | 0 | 0 | 1 | 0 | 0 | 0 | 0 | 0 |
| LMGH83 | 2 | 0 | 0 | 0 | 0 | 0 | 0 | 0 | 0 | 0 | 0 | 0 | 0 | 0 | 0 | 0 | 0 | 0 | 0 | 0 | 0 | 0 | 0 | 0 | 0 | 0 | 0 | 0 | 0 | 0 | 0 | 0 | 0 |
| LMGH95 | 2 | 0 | 0 | 0 | 0 | 0 | 0 | 0 | 0 | 0 | 0 | 0 | 0 | 0 | 0 | 0 | 0 | 0 | 0 | 0 | 0 | 0 | 0 | 0 | 0 | 0 | 0 | 0 | 0 | 0 | 0 | 0 | 0 |
| LMGH97 | 1 | 0 | 0 | 0 | 0 | 0 | 0 | 0 | 0 | 0 | 0 | 0 | 0 | 0 | 0 | 0 | 0 | 0 | 0 | 0 | 1 | 0 | 0 | 0 | 0 | 0 | 0 | 0 | 0 | 0 | 0 | 0 | 0 |
| LMGTW1 | 1 | 0 | 0 | 0 | 0 | 0 | 0 | 0 | 1 | 1 | 0 | 0 | 0 | 0 | 0 | 0 | 0 | 0 | 0 | 0 | 0 | 0 | 0 | 0 | 0 | 0 | 0 | 0 | 0 | 0 | 0 | 0 | 0 |
| LMGTW3 | 1 | 0 | 0 | 0 | 0 | 0 | 0 | 0 | 0 | 0 | 0 | 0 | 0 | 0 | 0 | 0 | 0 | 0 | 1 | 0 | 0 | 0 | 0 | 0 | 0 | 0 | 0 | 0 | 0 | 0 | 0 | 0 | 0 |
| LMGTW6 | 2 | 0 | 0 | 0 | 0 | 0 | 0 | 0 | 0 | 0 | 0 | 1 | 0 | 0 | 0 | 0 | 0 | 0 | 0 | 0 | 0 | 0 | 0 | 0 | 0 | 0 | 0 | 0 | 0 | 0 | 0 | 0 | 0 |
| LMGTW8 | 0 | 0 | 0 | 0 | 0 | 0 | 0 | 0 | 0 | 0 | 0 | 0 | 0 | 0 | 0 | 0 | 0 | 0 | 1 | 0 | 0 | 0 | 0 | 0 | 1 | 0 | 0 | 0 | 0 | 1 | 0 | 0 | 0 |
| LN02 | 1 | 0 | 0 | 0 | 0 | 0 | 0 | 0 | 0 | 1 | 0 | 0 | 0 | 0 | 0 | 0 | 0 | 0 | 0 | 0 | 0 | 0 | 0 | 0 | 0 | 0 | 0 | 0 | 0 | 0 | 0 | 0 | 0 |
| LN12 | 1 | 0 | 0 | 0 | 0 | 0 | 0 | 0 | 0 | 0 | 0 | 0 | 0 | 0 | 0 | 0 | 0 | 0 | 1 | 0 | 0 | 0 | 0 | 0 | 0 | 0 | 0 | 0 | 0 | 0 | 0 | 0 | 0 |
| MGBC116435 | 0 | 0 | 0 | 0 | 0 | 0 | 0 | 0 | 0 | 0 | 0 | 0 | 0 | 0 | 0 | 0 | 0 | 0 | 2 | 0 | 1 | 0 | 0 | 0 | 1 | 0 | 0 | 0 | 0 | 0 | 1 | 0 | 0 |
| NCDO_768 | 0 | 0 | 0 | 0 | 0 | 0 | 0 | 1 | 0 | 0 | 0 | 0 | 0 | 0 | 0 | 0 | 0 | 0 | 2 | 0 | 1 | 0 | 0 | 0 | 0 | 0 | 0 | 0 | 0 | 0 | 0 | 0 | 0 |
| PS12 | 0 | 0 | 0 | 0 | 0 | 0 | 0 | 0 | 1 | 0 | 0 | 0 | 0 | 0 | 0 | 0 | 0 | 0 | 0 | 0 | 0 | 0 | 0 | 0 | 0 | 1 | 0 | 0 | 0 | 0 | 0 | 0 | 0 |
| TMW21073 | 0 | 0 | 0 | 0 | 0 | 0 | 0 | 0 | 0 | 0 | 0 | 0 | 0 | 0 | 0 | 0 | 0 | 0 | 1 | 0 | 0 | 0 | 0 | 1 | 1 | 0 | 1 | 0 | 0 | 0 | 0 | 0 | 0 |
| TMW21195 | 1 | 0 | 0 | 0 | 0 | 0 | 0 | 0 | 0 | 0 | 0 | 0 | 0 | 0 | 0 | 0 | 0 | 0 | 1 | 0 | 0 | 0 | 0 | 0 | 0 | 0 | 0 | 0 | 0 | 0 | 0 | 0 | 0 |
| TR070 | 3 | 0 | 0 | 0 | 0 | 0 | 0 | 0 | 0 | 0 | 0 | 1 | 0 | 0 | 0 | 0 | 0 | 0 | 0 | 0 | 0 | 0 | 1 | 0 | 0 | 0 | 0 | 0 | 0 | 0 | 0 | 0 | 0 |

**Supplementary Table 5.** Putative secondary metabolites potential of thirty-eight *Ln. pseudomesenteroides* genomes.

| **Strain** | **Reference** | **Similarity score** | **Type** | **Compound(s)** | **Organism** |
| --- | --- | --- | --- | --- | --- |
| 1159 | [BGC0001120.1](https://mibig.secondarymetabolites.org/repository/BGC0001120/index.html#r1c1) | 0.16 | NRP, Polyketide | burkholderic acid | Burkholderia thailandensis E264 |
| 1159 | [BGC0000719.1](https://mibig.secondarymetabolites.org/repository/BGC0000719/index.html#r1c1) | 0.16 | Saccharide | tobramycin | Streptoalloteichus hindustanus |
| 1159 | [BGC0000554.1](https://mibig.secondarymetabolites.org/repository/BGC0000554/index.html#r1c1) | 0.16 | RiPP | SRO15-3108 | Streptomyces filamentosus NRRL 15998 |
| 1159 | [BGC0000720.1](https://mibig.secondarymetabolites.org/repository/BGC0000720/index.html#r1c1) | 0.16 | Saccharide | tobramycin | Streptoalloteichus tenebrarius |
| 1159 | [BGC0001850.1](https://mibig.secondarymetabolites.org/repository/BGC0001850/index.html#r1c1) | 0.14 | Other (Shikimate-derived) | diazaquinomycin A, diazaquinomycin E, diazaquinomycin F, diazaquinomycin G | Streptomyces sp. F001 |
| 1159 | [BGC0000783.1](https://mibig.secondarymetabolites.org/repository/BGC0000783/index.html#r1c1) | 0.14 | Saccharide | O-antigen | Xanthomonas oryzae pv. oryzae |
| 1159 | [BGC0000934.1](https://mibig.secondarymetabolites.org/repository/BGC0000934/index.html#r1c1) | 0.13 | Other (Phenazine) | 5-acetyl-5,10-dihydrophenazine-1-carboxylic acid, 5-(2-hydroxyacetyl)-5,10-dihydrophenazine-1-carboxylic acid, endophenazine A1, endophenazine F, endophenazine G | Kitasatospora sp. HKI 714 |
| 1159 | [BGC0000243.1](https://mibig.secondarymetabolites.org/repository/BGC0000243/index.html#r1c1) | 0.13 | Polyketide | macrotetrolide | Streptomyces griseus subsp. griseus |
| 1159 | [BGC0000916.1](https://mibig.secondarymetabolites.org/repository/BGC0000916/index.html#r1c1) | 0.13 | Other | molybdenum cofactor | Staphylococcus carnosus |
| 1159 | [BGC0001540.1](https://mibig.secondarymetabolites.org/repository/BGC0001540/index.html#r1c1) | 0.13 | Other | CC-1065 | Streptomyces zelensis |
| 17-2 | BGC0001594.1 | 0.26 | Alkaloid | fischerindole L | Fischerella muscicola UTEX 1829 |
| 17-2 | BGC0000668.1 | 0.24 | Terpene, Alkaloid | 12-epi-hapalindole C isonitrile, 12-epi-hapalindole E, 12-epi-fischerindole U isonitrile, fischerindole L, 12-epi-fischerindole I isonitrile, welwitindolinone A isonitrile, welwitindolinone B isothiocyanate, welwitindolinone C isothiocyanate, N-methylwelwitindolinone C isothiocyanate, N-methylwelwitinsolinone C isonitrile, 3-epi-welwitindolinone B isothiocyanate, 3-(Z-2'-isocyanoethenyl)-indole | Hapalosiphon welwitschii UTEX B 1830 |
| 17-2 | BGC0001595.1 | 0.22 | Alkaloid | fischerindole | Fischerella sp. SAG 46.79 |
| 17-2 | BGC0001126.1 | 0.21 | Terpene, Alkaloid | 12-epi-hapalindole J isonitrile, ambiguine A isonitrile, ambiguine B isonitrile, ambiguine C isonitrile, ambiguine D isonitrile, ambiguine E isonitrile, ambiguine K isonitrile, ambiguine L isonitrile, ambiguine I isonitrile, ambiguine J isonitrile | Fischerella ambigua UTEX 1903 |
| 17-2 | BGC0001612.1 | 0.21 | Alkaloid | ambiguine H isonitrile | Fischerella ambigua UTEX 1903 |
| 17-2 | BGC0001501.1 | 0.21 | Alkaloid | ambiguine P | Fischerella sp. TAU |
| 17-2 | BGC0001120.1 | 0.16 | NRP, Polyketide | burkholderic acid | Burkholderia thailandensis E264 |
| 17-2 | BGC0001664.1 | 0.16 | Terpene | merosterol | Scytonema sp. PCC 10023 |
| 17-2 | BGC0002009.1 | 0.16 | Polyketide | kanglemycin A, kanglemycin V1, kanglemycin V2 | Amycolatopsis vancoresmycina |
| 17-2 | BGC0001591.1 | 0.14 | Other | fatty acid enol ester | uncultured bacterium CSLC2 |
| 17-2 | BGC0000647.1 | 0.3 | Terpene | carotenoid | Rhodobacter sphaeroides |
| 17-2 | BGC0000648.1 | 0.22 | Terpene | carotenoid | Myxococcus xanthus |
| 17-2 | BGC0000646.1 | 0.21 | Terpene | β-carotein | uncultured bacterium |
| 17-2 | BGC0000643.1 | 0.2 | Terpene | carotenoid | Brevundimonas vesicularis |
| 17-2 | BGC0000634.1 | 0.2 | Terpene | carotenoid | Brevundimonas sp. SD212 |
| 17-2 | BGC0000664.1 | 0.2 | Terpene | isorenieratene | Streptomyces griseus subsp. griseus NBRC 13350 |
| 17-2 | BGC0000630.1 | 0.19 | Terpene | (2R,3S,3'S)-2-hydroxyastaxanthin | Paracoccus haeundaensis |
| 17-2 | BGC0000645.1 | 0.19 | Terpene | carotenoid | Halobacillus halophilus DSM 2266 |
| 17-2 | BGC0001456.1 | 0.18 | Terpene | isorenieratene | Streptomyces argillaceus |
| 17-2 | BGC0000640.1 | 0.18 | Terpene | carotenoid | Enterobacteriaceae bacterium DC404 |
| AMBR10 | BGC0000617.1 | 0.21 | RiPP | coagulin | Bacillus coagulans |
| AMBR10 | BGC0000802.1 | 0.16 | Saccharide | succinoglycan | Sinorhizobium meliloti |
| AMBR10 | BGC0001862.1 | 0.16 | RiPP | geocillicin | Aeribacillus pallidus |
| AMBR10 | BGC0001930.1 | 0.16 | RiPP | pallidocin | Aeribacillus pallidus |
| AMBR10 | BGC0001863.1 | 0.16 | RiPP, Terpene | bacillicn CER074 | Bacillus mycoides |
| AMBR10 | BGC0001388.1 | 0.16 | RiPP | gassericin E | Lactobacillus gasseri |
| AMBR10 | BGC0000619.1 | 0.16 | RiPP | gassericin T | Lactobacillus gasseri |
| AMBR10 | BGC0001602.1 | 0.16 | RiPP | gassericin-T | Lactobacillus gasseri |
| AMBR10 | BGC0001861.1 | 0.16 | RiPP | bacillicin BAG2O | Bacillus cereus BAG2O-1 |
| AMBR10 | BGC0000558.1 | 0.16 | RiPP | sublancin 168 | Bacillus subtilis subsp. subtilis str. 168 |
| AMBR10 | BGC0001594.1 | 0.26 | Alkaloid | fischerindole L | Fischerella muscicola UTEX 1829 |
| AMBR10 | BGC0000668.1 | 0.24 | Terpene, Alkaloid | 12-epi-hapalindole C isonitrile, 12-epi-hapalindole E, 12-epi-fischerindole U isonitrile, fischerindole L, 12-epi-fischerindole I isonitrile, welwitindolinone A isonitrile, welwitindolinone B isothiocyanate, welwitindolinone C isothiocyanate, N-methylwelwitindolinone C isothiocyanate, N-methylwelwitinsolinone C isonitrile, 3-epi-welwitindolinone B isothiocyanate, 3-(Z-2'-isocyanoethenyl)-indole | Hapalosiphon welwitschii UTEX B 1830 |
| AMBR10 | BGC0001595.1 | 0.22 | Alkaloid | fischerindole | Fischerella sp. SAG 46.79 |
| AMBR10 | BGC0001126.1 | 0.21 | Terpene, Alkaloid | 12-epi-hapalindole J isonitrile, ambiguine A isonitrile, ambiguine B isonitrile, ambiguine C isonitrile, ambiguine D isonitrile, ambiguine E isonitrile, ambiguine K isonitrile, ambiguine L isonitrile, ambiguine I isonitrile, ambiguine J isonitrile | Fischerella ambigua UTEX 1903 |
| AMBR10 | BGC0001612.1 | 0.21 | Alkaloid | ambiguine H isonitrile | Fischerella ambigua UTEX 1903 |
| AMBR10 | BGC0001501.1 | 0.21 | Alkaloid | ambiguine P | Fischerella sp. TAU |
| AMBR10 | BGC0001120.1 | 0.16 | NRP, Polyketide | burkholderic acid | Burkholderia thailandensis E264 |
| AMBR10 | BGC0001664.1 | 0.16 | Terpene | merosterol | Scytonema sp. PCC 10023 |
| AMBR10 | BGC0000554.1 | 0.16 | RiPP | SRO15-3108 | Streptomyces filamentosus NRRL 15998 |
| AMBR10 | BGC0002009.1 | 0.15 | Polyketide | kanglemycin A, kanglemycin V1, kanglemycin V2 | Amycolatopsis vancoresmycina |
| BM2 | BGC0001120.1 | 0.16 | NRP, Polyketide | burkholderic acid | Burkholderia thailandensis E264 |
| BM2 | BGC0000719.1 | 0.16 | Saccharide | tobramycin | Streptoalloteichus hindustanus |
| BM2 | BGC0000554.1 | 0.16 | RiPP | SRO15-3108 | Streptomyces filamentosus NRRL 15998 |
| BM2 | BGC0000720.1 | 0.16 | Saccharide | tobramycin | Streptoalloteichus tenebrarius |
| BM2 | BGC0001104.1 | 0.16 | NRP, Polyketide | myxovirescin A1 | Myxococcus xanthus DK 1622 |
| BM2 | BGC0000528.1 | 0.15 | RiPP | michiganin A | Clavibacter michiganensis subsp. michiganensis NCPPB 382 |
| BM2 | BGC0001964.1 | 0.15 | Polyketide | alkylpyrone-407, alkylpyrone-393 | Cystobacterineae bacterium |
| BM2 | BGC0001591.1 | 0.14 | Other | fatty acid enol ester | uncultured bacterium CSLC2 |
| BM2 | BGC0001688.1 | 0.14 | Other | N-tetradecanoyl tyrosine | uncultured bacterium CSLC2 |
| BM2 | BGC0001927.1 | 0.14 | Other | A-94964 | Streptomyces sp. |
| CBA3630 | BGC0001594.1 | 0.26 | Alkaloid | fischerindole L | Fischerella muscicola UTEX 1829 |
| CBA3630 | BGC0000668.1 | 0.24 | Terpene, Alkaloid | 12-epi-hapalindole C isonitrile, 12-epi-hapalindole E, 12-epi-fischerindole U isonitrile, fischerindole L, 12-epi-fischerindole I isonitrile, welwitindolinone A isonitrile, welwitindolinone B isothiocyanate, welwitindolinone C isothiocyanate, N-methylwelwitindolinone C isothiocyanate, N-methylwelwitinsolinone C isonitrile, 3-epi-welwitindolinone B isothiocyanate, 3-(Z-2'-isocyanoethenyl)-indole | Hapalosiphon welwitschii UTEX B 1830 |
| CBA3630 | BGC0001595.1 | 0.22 | Alkaloid | fischerindole | Fischerella sp. SAG 46.79 |
| CBA3630 | BGC0001126.1 | 0.21 | Terpene, Alkaloid | 12-epi-hapalindole J isonitrile, ambiguine A isonitrile, ambiguine B isonitrile, ambiguine C isonitrile, ambiguine D isonitrile, ambiguine E isonitrile, ambiguine K isonitrile, ambiguine L isonitrile, ambiguine I isonitrile, ambiguine J isonitrile | Fischerella ambigua UTEX 1903 |
| CBA3630 | BGC0001612.1 | 0.21 | Alkaloid | ambiguine H isonitrile | Fischerella ambigua UTEX 1903 |
| CBA3630 | BGC0001501.1 | 0.21 | Alkaloid | ambiguine P | Fischerella sp. TAU |
| CBA3630 | BGC0001120.1 | 0.16 | NRP, Polyketide | burkholderic acid | Burkholderia thailandensis E264 |
| CBA3630 | BGC0000719.1 | 0.16 | Saccharide | tobramycin | Streptoalloteichus hindustanus |
| CBA3630 | BGC0000720.1 | 0.16 | Saccharide | tobramycin | Streptoalloteichus tenebrarius |
| CBA3630 | BGC0002009.1 | 0.16 | Polyketide | kanglemycin A, kanglemycin V1, kanglemycin V2 | Amycolatopsis vancoresmycina |
| Dm-9 | BGC0000617.1 | 0.21 | RiPP | coagulin | Bacillus coagulans |
| Dm-9 | BGC0000802.1 | 0.16 | Saccharide | succinoglycan | Sinorhizobium meliloti |
| Dm-9 | BGC0001862.1 | 0.16 | RiPP | geocillicin | Aeribacillus pallidus |
| Dm-9 | BGC0001930.1 | 0.16 | RiPP | pallidocin | Aeribacillus pallidus |
| Dm-9 | BGC0001388.1 | 0.16 | RiPP | gassericin E | Lactobacillus gasseri |
| Dm-9 | BGC0001863.1 | 0.16 | RiPP, Terpene | bacillicn CER074 | Bacillus mycoides |
| Dm-9 | BGC0000619.1 | 0.16 | RiPP | gassericin T | Lactobacillus gasseri |
| Dm-9 | BGC0001602.1 | 0.16 | RiPP | gassericin-T | Lactobacillus gasseri |
| Dm-9 | BGC0001861.1 | 0.16 | RiPP | bacillicin BAG2O | Bacillus cereus BAG2O-1 |
| Dm-9 | BGC0000558.1 | 0.16 | RiPP | sublancin 168 | Bacillus subtilis subsp. subtilis str. 168 |
| Dm-9 | BGC0001594.1 | 0.26 | Alkaloid | fischerindole L | Fischerella muscicola UTEX 1829 |
| Dm-9 | BGC0000668.1 | 0.24 | Terpene, Alkaloid | 12-epi-hapalindole C isonitrile, 12-epi-hapalindole E, 12-epi-fischerindole U isonitrile, fischerindole L, 12-epi-fischerindole I isonitrile, welwitindolinone A isonitrile, welwitindolinone B isothiocyanate, welwitindolinone C isothiocyanate, N-methylwelwitindolinone C isothiocyanate, N-methylwelwitinsolinone C isonitrile, 3-epi-welwitindolinone B isothiocyanate, 3-(Z-2'-isocyanoethenyl)-indole | Hapalosiphon welwitschii UTEX B 1830 |
| Dm-9 | BGC0001595.1 | 0.22 | Alkaloid | fischerindole | Fischerella sp. SAG 46.79 |
| Dm-9 | BGC0001126.1 | 0.21 | Terpene, Alkaloid | 12-epi-hapalindole J isonitrile, ambiguine A isonitrile, ambiguine B isonitrile, ambiguine C isonitrile, ambiguine D isonitrile, ambiguine E isonitrile, ambiguine K isonitrile, ambiguine L isonitrile, ambiguine I isonitrile, ambiguine J isonitrile | Fischerella ambigua UTEX 1903 |
| Dm-9 | BGC0001612.1 | 0.21 | Alkaloid | ambiguine H isonitrile | Fischerella ambigua UTEX 1903 |
| Dm-9 | BGC0001501.1 | 0.21 | Alkaloid | ambiguine P | Fischerella sp. TAU |
| Dm-9 | BGC0001120.1 | 0.16 | NRP, Polyketide | burkholderic acid | Burkholderia thailandensis E264 |
| Dm-9 | BGC0001664.1 | 0.16 | Terpene | merosterol | Scytonema sp. PCC 10023 |
| Dm-9 | BGC0002009.1 | 0.15 | Polyketide | kanglemycin A, kanglemycin V1, kanglemycin V2 | Amycolatopsis vancoresmycina |
| Dm-9 | BGC0001591.1 | 0.14 | Other | fatty acid enol ester | uncultured bacterium CSLC2 |
| Dm-9 | BGC0000647.1 | 0.3 | Terpene | carotenoid | Rhodobacter sphaeroides |
| Dm-9 | BGC0000648.1 | 0.22 | Terpene | carotenoid | Myxococcus xanthus |
| Dm-9 | BGC0000643.1 | 0.2 | Terpene | carotenoid | Brevundimonas vesicularis |
| Dm-9 | BGC0000646.1 | 0.2 | Terpene | β-carotein | uncultured bacterium |
| Dm-9 | BGC0000634.1 | 0.2 | Terpene | carotenoid | Brevundimonas sp. SD212 |
| Dm-9 | BGC0000664.1 | 0.19 | Terpene | isorenieratene | Streptomyces griseus subsp. griseus NBRC 13350 |
| Dm-9 | BGC0000630.1 | 0.19 | Terpene | (2R,3S,3'S)-2-hydroxyastaxanthin | Paracoccus haeundaensis |
| Dm-9 | BGC0000645.1 | 0.18 | Terpene | carotenoid | Halobacillus halophilus DSM 2266 |
| Dm-9 | BGC0001456.1 | 0.18 | Terpene | isorenieratene | Streptomyces argillaceus |
| Dm-9 | BGC0000640.1 | 0.18 | Terpene | carotenoid | Enterobacteriaceae bacterium DC404 |
| FDAARGOS_1003 | BGC0001594.1 | 0.26 | Alkaloid | fischerindole L | Fischerella muscicola UTEX 1829 |
| FDAARGOS_1003 | BGC0000668.1 | 0.24 | Terpene, Alkaloid | 12-epi-hapalindole C isonitrile, 12-epi-hapalindole E, 12-epi-fischerindole U isonitrile, fischerindole L, 12-epi-fischerindole I isonitrile, welwitindolinone A isonitrile, welwitindolinone B isothiocyanate, welwitindolinone C isothiocyanate, N-methylwelwitindolinone C isothiocyanate, N-methylwelwitinsolinone C isonitrile, 3-epi-welwitindolinone B isothiocyanate, 3-(Z-2'-isocyanoethenyl)-indole | Hapalosiphon welwitschii UTEX B 1830 |
| FDAARGOS_1003 | BGC0001595.1 | 0.22 | Alkaloid | fischerindole | Fischerella sp. SAG 46.79 |
| FDAARGOS_1003 | BGC0001126.1 | 0.21 | Terpene, Alkaloid | 12-epi-hapalindole J isonitrile, ambiguine A isonitrile, ambiguine B isonitrile, ambiguine C isonitrile, ambiguine D isonitrile, ambiguine E isonitrile, ambiguine K isonitrile, ambiguine L isonitrile, ambiguine I isonitrile, ambiguine J isonitrile | Fischerella ambigua UTEX 1903 |
| FDAARGOS_1003 | BGC0001612.1 | 0.21 | Alkaloid | ambiguine H isonitrile | Fischerella ambigua UTEX 1903 |
| FDAARGOS_1003 | BGC0001501.1 | 0.21 | Alkaloid | ambiguine P | Fischerella sp. TAU |
| FDAARGOS_1003 | BGC0001120.1 | 0.16 | NRP, Polyketide | burkholderic acid | Burkholderia thailandensis E264 |
| FDAARGOS_1003 | BGC0000719.1 | 0.16 | Saccharide | tobramycin | Streptoalloteichus hindustanus |
| FDAARGOS_1003 | BGC0000720.1 | 0.16 | Saccharide | tobramycin | Streptoalloteichus tenebrarius |
| FDAARGOS_1003 | BGC0002009.1 | 0.16 | Polyketide | kanglemycin A, kanglemycin V1, kanglemycin V2 | Amycolatopsis vancoresmycina |
| FDAARGOS_1004 | BGC0001594.1 | 0.26 | Alkaloid | fischerindole L | Fischerella muscicola UTEX 1829 |
| FDAARGOS_1004 | BGC0000668.1 | 0.24 | Terpene, Alkaloid | 12-epi-hapalindole C isonitrile, 12-epi-hapalindole E, 12-epi-fischerindole U isonitrile, fischerindole L, 12-epi-fischerindole I isonitrile, welwitindolinone A isonitrile, welwitindolinone B isothiocyanate, welwitindolinone C isothiocyanate, N-methylwelwitindolinone C isothiocyanate, N-methylwelwitinsolinone C isonitrile, 3-epi-welwitindolinone B isothiocyanate, 3-(Z-2'-isocyanoethenyl)-indole | Hapalosiphon welwitschii UTEX B 1830 |
| FDAARGOS_1004 | BGC0001595.1 | 0.22 | Alkaloid | fischerindole | Fischerella sp. SAG 46.79 |
| FDAARGOS_1004 | BGC0001126.1 | 0.21 | Terpene, Alkaloid | 12-epi-hapalindole J isonitrile, ambiguine A isonitrile, ambiguine B isonitrile, ambiguine C isonitrile, ambiguine D isonitrile, ambiguine E isonitrile, ambiguine K isonitrile, ambiguine L isonitrile, ambiguine I isonitrile, ambiguine J isonitrile | Fischerella ambigua UTEX 1903 |
| FDAARGOS_1004 | BGC0001612.1 | 0.21 | Alkaloid | ambiguine H isonitrile | Fischerella ambigua UTEX 1903 |
| FDAARGOS_1004 | BGC0001501.1 | 0.21 | Alkaloid | ambiguine P | Fischerella sp. TAU |
| FDAARGOS_1004 | BGC0001120.1 | 0.16 | NRP, Polyketide | burkholderic acid | Burkholderia thailandensis E264 |
| FDAARGOS_1004 | BGC0001664.1 | 0.16 | Terpene | merosterol | Scytonema sp. PCC 10023 |
| FDAARGOS_1004 | BGC0000554.1 | 0.16 | RiPP | SRO15-3108 | Streptomyces filamentosus NRRL 15998 |
| FDAARGOS_1004 | BGC0002009.1 | 0.15 | Polyketide | kanglemycin A, kanglemycin V1, kanglemycin V2 | Amycolatopsis vancoresmycina |
| HPK01 | BGC0000286.1 | 0.22 | Polyketide | viguiepinol | Streptomyces sp. KO-3988 |
| HPK01 | BGC0001120.1 | 0.16 | NRP, Polyketide | burkholderic acid | Burkholderia thailandensis E264 |
| HPK01 | BGC0000205.1 | 0.16 | Polyketide | bryostatin | Candidatus Endobugula sertula |
| HPK01 | BGC0000554.1 | 0.16 | RiPP | SRO15-3108 | Streptomyces filamentosus NRRL 15998 |
| HPK01 | BGC0001551.1 | 0.15 | RiPP | citrulassin E | Streptomyces glaucescens |
| HPK01 | BGC0001407.1 | 0.15 | RiPP | bicereucin | Bacillus cereus SJ1 |
| HPK01 | BGC0000504.1 | 0.15 | RiPP | cytolysin ClyLl, cytolysin ClyLs | Plasmid pAD1 |
| HPK01 | BGC0001863.1 | 0.15 | RiPP, Terpene | bacillicn CER074 | Bacillus mycoides |
| HPK01 | BGC0001783.1 | 0.15 | Other | streptonigrin | Streptomyces flocculus |
| HPK01 | BGC0000255.1 | 0.15 | Polyketide | pederin | Uncultured bacterium |
| IM1374 | BGC0001120.1 | 0.16 | NRP, Polyketide | burkholderic acid | Burkholderia thailandensis E264 |
| IM1374 | BGC0000719.1 | 0.16 | Saccharide | tobramycin | Streptoalloteichus hindustanus |
| IM1374 | BGC0000554.1 | 0.16 | RiPP | SRO15-3108 | Streptomyces filamentosus NRRL 15998 |
| IM1374 | BGC0001850.1 | 0.14 | Other (Shikimate-derived) | diazaquinomycin A, diazaquinomycin E, diazaquinomycin F, diazaquinomycin G | Streptomyces sp. F001 |
| IM1374 | BGC0000783.1 | 0.14 | Saccharide | O-antigen | Xanthomonas oryzae pv. oryzae |
| IM1374 | BGC0000934.1 | 0.13 | Other (Phenazine) | 5-acetyl-5,10-dihydrophenazine-1-carboxylic acid, 5-(2-hydroxyacetyl)-5,10-dihydrophenazine-1-carboxylic acid, endophenazine A1, endophenazine F, endophenazine G | Kitasatospora sp. HKI 714 |
| IM1374 | BGC0000720.1 | 0.13 | Saccharide | tobramycin | Streptoalloteichus tenebrarius |
| IM1374 | BGC0000243.1 | 0.13 | Polyketide | macrotetrolide | Streptomyces griseus subsp. griseus |
| IM1374 | BGC0001540.1 | 0.13 | Other | CC-1065 | Streptomyces zelensis |
| IM1374 | BGC0000785.1 | 0.13 | Saccharide | O-antigen | Burkholderia mallei |
| IM1427 | BGC0001120.1 | 0.16 | NRP, Polyketide | burkholderic acid | Burkholderia thailandensis E264 |
| IM1427 | BGC0001639.1 | 0.16 | RiPP | klebsidin | Klebsiella pneumoniae |
| IM1427 | BGC0000719.1 | 0.16 | Saccharide | tobramycin | Streptoalloteichus hindustanus |
| IM1427 | BGC0001779.1 | 0.16 | RiPP | siamycin | Streptomyces nodosus |
| IM1427 | BGC0000554.1 | 0.16 | RiPP | SRO15-3108 | Streptomyces filamentosus NRRL 15998 |
| IM1427 | BGC0000720.1 | 0.16 | Saccharide | tobramycin | Streptoalloteichus tenebrarius |
| IM1427 | BGC0000539.1 | 0.16 | RiPP | nukacin ISK-1 | Staphylococcus warneri |
| IM1427 | BGC0001591.1 | 0.14 | Other | fatty acid enol ester | uncultured bacterium CSLC2 |
| IM1427 | BGC0001688.1 | 0.14 | Other | N-tetradecanoyl tyrosine | uncultured bacterium CSLC2 |
| IM1427 | BGC0001225.1 | 0.14 | NRP | celesticetin | Streptomyces caelestis |
| KMB_610 | BGC0001594.1 | 0.26 | Alkaloid | fischerindole L | Fischerella muscicola UTEX 1829 |
| KMB_610 | BGC0000668.1 | 0.24 | Terpene, Alkaloid | 12-epi-hapalindole C isonitrile, 12-epi-hapalindole E, 12-epi-fischerindole U isonitrile, fischerindole L, 12-epi-fischerindole I isonitrile, welwitindolinone A isonitrile, welwitindolinone B isothiocyanate, welwitindolinone C isothiocyanate, N-methylwelwitindolinone C isothiocyanate, N-methylwelwitinsolinone C isonitrile, 3-epi-welwitindolinone B isothiocyanate, 3-(Z-2'-isocyanoethenyl)-indole | Hapalosiphon welwitschii UTEX B 1830 |
| KMB_610 | BGC0001595.1 | 0.22 | Alkaloid | fischerindole | Fischerella sp. SAG 46.79 |
| KMB_610 | BGC0001126.1 | 0.21 | Terpene, Alkaloid | 12-epi-hapalindole J isonitrile, ambiguine A isonitrile, ambiguine B isonitrile, ambiguine C isonitrile, ambiguine D isonitrile, ambiguine E isonitrile, ambiguine K isonitrile, ambiguine L isonitrile, ambiguine I isonitrile, ambiguine J isonitrile | Fischerella ambigua UTEX 1903 |
| KMB_610 | BGC0001612.1 | 0.21 | Alkaloid | ambiguine H isonitrile | Fischerella ambigua UTEX 1903 |
| KMB_610 | BGC0001501.1 | 0.21 | Alkaloid | ambiguine P | Fischerella sp. TAU |
| KMB_610 | BGC0001120.1 | 0.16 | NRP, Polyketide | burkholderic acid | Burkholderia thailandensis E264 |
| KMB_610 | BGC0002009.1 | 0.16 | Polyketide | kanglemycin A, kanglemycin V1, kanglemycin V2 | Amycolatopsis vancoresmycina |
| KMB_610 | BGC0001664.1 | 0.16 | Terpene | merosterol | Scytonema sp. PCC 10023 |
| KMB_610 | BGC0000554.1 | 0.16 | RiPP | SRO15-3108 | Streptomyces filamentosus NRRL 15998 |
| LMG_11482 | BGC0001594.1 | 0.26 | Alkaloid | fischerindole L | Fischerella muscicola UTEX 1829 |
| LMG_11482 | BGC0000668.1 | 0.24 | Terpene, Alkaloid | 12-epi-hapalindole C isonitrile, 12-epi-hapalindole E, 12-epi-fischerindole U isonitrile, fischerindole L, 12-epi-fischerindole I isonitrile, welwitindolinone A isonitrile, welwitindolinone B isothiocyanate, welwitindolinone C isothiocyanate, N-methylwelwitindolinone C isothiocyanate, N-methylwelwitinsolinone C isonitrile, 3-epi-welwitindolinone B isothiocyanate, 3-(Z-2'-isocyanoethenyl)-indole | Hapalosiphon welwitschii UTEX B 1830 |
| LMG_11482 | BGC0001595.1 | 0.22 | Alkaloid | fischerindole | Fischerella sp. SAG 46.79 |
| LMG_11482 | BGC0001126.1 | 0.21 | Terpene, Alkaloid | 12-epi-hapalindole J isonitrile, ambiguine A isonitrile, ambiguine B isonitrile, ambiguine C isonitrile, ambiguine D isonitrile, ambiguine E isonitrile, ambiguine K isonitrile, ambiguine L isonitrile, ambiguine I isonitrile, ambiguine J isonitrile | Fischerella ambigua UTEX 1903 |
| LMG_11482 | BGC0001612.1 | 0.21 | Alkaloid | ambiguine H isonitrile | Fischerella ambigua UTEX 1903 |
| LMG_11482 | BGC0001501.1 | 0.21 | Alkaloid | ambiguine P | Fischerella sp. TAU |
| LMG_11482 | BGC0001120.1 | 0.16 | NRP, Polyketide | burkholderic acid | Burkholderia thailandensis E264 |
| LMG_11482 | BGC0000719.1 | 0.16 | Saccharide | tobramycin | Streptoalloteichus hindustanus |
| LMG_11482 | BGC0000720.1 | 0.16 | Saccharide | tobramycin | Streptoalloteichus tenebrarius |
| LMG_11482 | BGC0002009.1 | 0.16 | Polyketide | kanglemycin A, kanglemycin V1, kanglemycin V2 | Amycolatopsis vancoresmycina |
| LMG_11483 | BGC0001594.1 | 0.26 | Alkaloid | fischerindole L | Fischerella muscicola UTEX 1829 |
| LMG_11483 | BGC0001595.1 | 0.25 | Alkaloid | fischerindole | Fischerella sp. SAG 46.79 |
| LMG_11483 | BGC0000668.1 | 0.24 | Terpene, Alkaloid | 12-epi-hapalindole C isonitrile, 12-epi-hapalindole E, 12-epi-fischerindole U isonitrile, fischerindole L, 12-epi-fischerindole I isonitrile, welwitindolinone A isonitrile, welwitindolinone B isothiocyanate, welwitindolinone C isothiocyanate, N-methylwelwitindolinone C isothiocyanate, N-methylwelwitinsolinone C isonitrile, 3-epi-welwitindolinone B isothiocyanate, 3-(Z-2'-isocyanoethenyl)-indole | Hapalosiphon welwitschii UTEX B 1830 |
| LMG_11483 | BGC0001126.1 | 0.21 | Terpene, Alkaloid | 12-epi-hapalindole J isonitrile, ambiguine A isonitrile, ambiguine B isonitrile, ambiguine C isonitrile, ambiguine D isonitrile, ambiguine E isonitrile, ambiguine K isonitrile, ambiguine L isonitrile, ambiguine I isonitrile, ambiguine J isonitrile | Fischerella ambigua UTEX 1903 |
| LMG_11483 | BGC0001612.1 | 0.21 | Alkaloid | ambiguine H isonitrile | Fischerella ambigua UTEX 1903 |
| LMG_11483 | BGC0001501.1 | 0.21 | Alkaloid | ambiguine P | Fischerella sp. TAU |
| LMG_11483 | BGC0001120.1 | 0.16 | NRP, Polyketide | burkholderic acid | Burkholderia thailandensis E264 |
| LMG_11483 | BGC0001664.1 | 0.16 | Terpene | merosterol | Scytonema sp. PCC 10023 |
| LMG_11483 | BGC0002009.1 | 0.16 | Polyketide | kanglemycin A, kanglemycin V1, kanglemycin V2 | Amycolatopsis vancoresmycina |
| LMG_11483 | BGC0001591.1 | 0.14 | Other | fatty acid enol ester | uncultured bacterium CSLC2 |
| LMGCF06 | BGC0000913.1 | 0.2 | Other | MK-8 | Enterobacter cloacae |
| LMGCF06 | BGC0001120.1 | 0.16 | NRP, Polyketide | burkholderic acid | Burkholderia thailandensis E264 |
| LMGCF06 | BGC0000768.1 | 0.16 | Saccharide | gellan polysaccharide | Sphingomonas elodea ATCC 31461 |
| LMGCF06 | BGC0001387.1 | 0.16 | Other | nucleocidin | Streptomyces calvus |
| LMGCF06 | BGC0000719.1 | 0.16 | Saccharide | tobramycin | Streptoalloteichus hindustanus |
| LMGCF06 | BGC0000720.1 | 0.16 | Saccharide | tobramycin | Streptoalloteichus tenebrarius |
| LMGCF06 | BGC0000554.1 | 0.16 | RiPP | SRO15-3108 | Streptomyces filamentosus NRRL 15998 |
| LMGCF06 | BGC0001104.1 | 0.16 | NRP, Polyketide | myxovirescin A1 | Myxococcus xanthus DK 1622 |
| LMGCF06 | BGC0001184.1 | 0.15 | Other | bacilysin | Bacillus velezensis FZB42 |
| LMGCF06 | BGC0001591.1 | 0.14 | Other | fatty acid enol ester | uncultured bacterium CSLC2 |
| LMGCF15 | BGC0000719.1 | 0.16 | Saccharide | tobramycin | Streptoalloteichus hindustanus |
| LMGCF15 | BGC0000720.1 | 0.16 | Saccharide | tobramycin | Streptoalloteichus tenebrarius |
| LMGCF15 | BGC0000554.1 | 0.16 | RiPP | SRO15-3108 | Streptomyces filamentosus NRRL 15998 |
| LMGCF15 | BGC0001591.1 | 0.14 | Other | fatty acid enol ester | uncultured bacterium CSLC2 |
| LMGCF15 | BGC0001688.1 | 0.14 | Other | N-tetradecanoyl tyrosine | uncultured bacterium CSLC2 |
| LMGCF15 | BGC0001927.1 | 0.14 | Other | A-94964 | Streptomyces sp. |
| LMGCF15 | BGC0001850.1 | 0.14 | Other (Shikimate-derived) | diazaquinomycin A, diazaquinomycin E, diazaquinomycin F, diazaquinomycin G | Streptomyces sp. F001 |
| LMGCF15 | BGC0000783.1 | 0.14 | Saccharide | O-antigen | Xanthomonas oryzae pv. oryzae |
| LMGCF15 | BGC0000243.1 | 0.13 | Polyketide | macrotetrolide | Streptomyces griseus subsp. griseus |
| LMGCF15 | BGC0001540.1 | 0.13 | Other | CC-1065 | Streptomyces zelensis |
| LMGH100 | BGC0001534.1 | 0.19 | Other | branched-chain fatty acids | Streptomyces filamentosus |
| LMGH100 | BGC0000523.1 | 0.17 | RiPP | lactocin S | Lactobacillus sakei |
| LMGH100 | BGC0000709.1 | 0.17 | Saccharide | neomycin | Streptomyces fradiae ATCC 10745 = DSM 40063 |
| LMGH100 | BGC0001535.1 | 0.17 | Other | branched-chain fatty acids | Streptomyces filamentosus |
| LMGH100 | BGC0000880.1 | 0.17 | Other (Nucleoside) | tunicamycin B1 | Streptomyces chartreusis NRRL 3882 |
| LMGH100 | BGC0001120.1 | 0.16 | NRP, Polyketide | burkholderic acid | Burkholderia thailandensis E264 |
| LMGH100 | BGC0000554.1 | 0.16 | RiPP | SRO15-3108 | Streptomyces filamentosus NRRL 15998 |
| LMGH100 | BGC0001104.1 | 0.16 | NRP, Polyketide | myxovirescin A1 | Myxococcus xanthus DK 1622 |
| LMGH100 | BGC0000539.1 | 0.15 | RiPP | nukacin ISK-1 | Staphylococcus warneri |
| LMGH100 | BGC0001521.1 | 0.14 | NRP | auriculamide | Herpetosiphon aurantiacus DSM 785 |
| LMGH278 | BGC0001526.1 | 0.18 | Other | bartolosides E, bartolosides F, bartolosides G, bartolosides H, bartolosides I, bartolosides J, bartolosides K | Synechocystis salina LEGE 06099 |
| LMGH278 | BGC0001525.1 | 0.18 | Other | bartoloside 2, bartoloside 3, bartoloside 4 | Synechocystis salina LEGE 06155 |
| LMGH278 | BGC0000767.1 | 0.17 | Saccharide | exopolysaccharide | Lactobacillus johnsonii |
| LMGH278 | BGC0000765.1 | 0.17 | Saccharide | exopolysaccharide | Lactobacillus johnsonii |
| LMGH278 | BGC0000766.1 | 0.17 | Saccharide | exopolysaccharide | Lactobacillus johnsonii |
| LMGH278 | BGC0001210.1 | 0.16 | RiPP | pseudomycoicidin | Bacillus pseudomycoides DSM 12442 |
| LMGH278 | BGC0000521.1 | 0.16 | RiPP | lacticin 481 | Lactococcus lactis subsp. lactis |
| LMGH278 | BGC0000719.1 | 0.16 | Saccharide | tobramycin | Streptoalloteichus hindustanus |
| LMGH278 | BGC0001407.1 | 0.16 | RiPP | bicereucin | Bacillus cereus SJ1 |
| LMGH278 | BGC0000720.1 | 0.16 | Saccharide | tobramycin | Streptoalloteichus tenebrarius |
| LMGH280 | BGC0000554.1 | 0.17 | RiPP | SRO15-3108 | Streptomyces filamentosus NRRL 15998 |
| LMGH280 | BGC0001526.1 | 0.16 | Other | bartolosides E, bartolosides F, bartolosides G, bartolosides H, bartolosides I, bartolosides J, bartolosides K | Synechocystis salina LEGE 06099 |
| LMGH280 | BGC0001525.1 | 0.16 | Other | bartoloside 2, bartoloside 3, bartoloside 4 | Synechocystis salina LEGE 06155 |
| LMGH280 | BGC0001948.1 | 0.16 | Other | naseseazine C, C3-aryl pyrroloindolines | Streptomyces sp. |
| LMGH280 | BGC0000500.1 | 0.16 | RiPP | carnolysin A1, carnolysin A2 | Carnobacterium maltaromaticum |
| LMGH280 | BGC0000551.1 | 0.16 | RiPP | SapB | Streptomyces coelicolor A3(2) |
| LMGH280 | BGC0000487.1 | 0.16 | RiPP | carnocyclin | Carnobacterium maltaromaticum |
| LMGH280 | BGC0001210.1 | 0.16 | RiPP | pseudomycoicidin | Bacillus pseudomycoides DSM 12442 |
| LMGH280 | BGC0000513.1 | 0.16 | RiPP | Ery-9, Ery-6, Ery-8, Ery-7, Ery-5, Ery-4, Ery-3 | Saccharopolyspora erythraea NRRL 2338 |
| LMGH280 | BGC0001241.1 | 0.16 | Terpene | ergotamine | Claviceps fusiformis |
| LMGH284 | BGC0000719.1 | 0.21 | Saccharide | tobramycin | Streptoalloteichus hindustanus |
| LMGH284 | BGC0000720.1 | 0.19 | Saccharide | tobramycin | Streptoalloteichus tenebrarius |
| LMGH284 | BGC0000494.1 | 0.19 | RiPP | uberolysin | Streptococcus uberis |
| LMGH284 | BGC0001120.1 | 0.16 | NRP, Polyketide | burkholderic acid | Burkholderia thailandensis E264 |
| LMGH284 | BGC0000721.1 | 0.16 | Saccharide | tobramycin | Streptoalloteichus tenebrarius |
| LMGH284 | BGC0000554.1 | 0.16 | RiPP | SRO15-3108 | Streptomyces filamentosus NRRL 15998 |
| LMGH284 | BGC0001104.1 | 0.16 | NRP, Polyketide | myxovirescin A1 | Myxococcus xanthus DK 1622 |
| LMGH284 | BGC0000716.1 | 0.15 | Saccharide | spectinomycin | Streptomyces netropsis |
| LMGH284 | BGC0001654.1 | 0.14 | Saccharide | lipooligosaccharide | Bacteroides thetaiotaomicron VPI-5482 |
| LMGH284 | BGC0001591.1 | 0.14 | Other | fatty acid enol ester | uncultured bacterium CSLC2 |
| LMGH61 | BGC0001579.1 | 0.18 | RiPP | duramycin | Streptomyces cinnamoneus |
| LMGH61 | BGC0000563.1 | 0.17 | RiPP | venezuelin | Streptomyces venezuelae ATCC 10712 |
| LMGH61 | BGC0001120.1 | 0.16 | NRP, Polyketide | burkholderic acid | Burkholderia thailandensis E264 |
| LMGH61 | BGC0000616.1 | 0.16 | RiPP | amylocyclicin | Bacillus velezensis FZB42 |
| LMGH61 | BGC0000521.1 | 0.16 | RiPP | lacticin 481 | Lactococcus lactis subsp. lactis |
| LMGH61 | BGC0000539.1 | 0.16 | RiPP | nukacin ISK-1 | Staphylococcus warneri |
| LMGH61 | BGC0001743.1 | 0.16 | RiPP | plantaricyclin | Lactobacillus plantarum |
| LMGH61 | BGC0000719.1 | 0.16 | Saccharide | tobramycin | Streptoalloteichus hindustanus |
| LMGH61 | BGC0001210.1 | 0.16 | RiPP | pseudomycoicidin | Bacillus pseudomycoides DSM 12442 |
| LMGH61 | BGC0000720.1 | 0.16 | Saccharide | tobramycin | Streptoalloteichus tenebrarius |
| LMGH83 | BGC0000753.1 | 0.2 | Saccharide | capsular polysaccharide | Actinobacillus pleuropneumoniae |
| LMGH83 | BGC0000752.1 | 0.2 | Saccharide | capsular polysaccharide | Actinobacillus pleuropneumoniae serovar 6 str. Femo |
| LMGH83 | BGC0000763.1 | 0.17 | Saccharide | stewartan | Pantoea stewartii subsp. stewartii DC283 |
| LMGH83 | BGC0000730.1 | 0.17 | Saccharide | capsular polysaccharide | Klebsiella pneumoniae |
| LMGH83 | BGC0000731.1 | 0.17 | Saccharide | capsular polysaccharide | Klebsiella pneumoniae |
| LMGH83 | BGC0001526.1 | 0.17 | Other | bartolosides E, bartolosides F, bartolosides G, bartolosides H, bartolosides I, bartolosides J, bartolosides K | Synechocystis salina LEGE 06099 |
| LMGH83 | BGC0001525.1 | 0.17 | Other | bartoloside 2, bartoloside 3, bartoloside 4 | Synechocystis salina LEGE 06155 |
| LMGH83 | BGC0000795.1 | 0.17 | Saccharide | S-layer glycan | Aneurinibacillus thermoaerophilus |
| LMGH83 | BGC0000783.1 | 0.17 | Saccharide | O-antigen | Xanthomonas oryzae pv. oryzae |
| LMGH83 | BGC0000487.1 | 0.16 | RiPP | carnocyclin | Carnobacterium maltaromaticum |
| LMGH95 | BGC0000887.1 | 0.17 | Alkaloid | saxitoxin, gonyautoxin 2, gonyautoxin 3, toxin C2, toxin C3, gonyautoxin 5, decarbamoylsaxitoxin, decarbamoylgonyautoxin 2, decarbamoylgonyautoxin 3 | Dolichospermum circinale AWQC131C |
| LMGH95 | BGC0001120.1 | 0.16 | NRP, Polyketide | burkholderic acid | Burkholderia thailandensis E264 |
| LMGH95 | BGC0000554.1 | 0.16 | RiPP | SRO15-3108 | Streptomyces filamentosus NRRL 15998 |
| LMGH95 | BGC0001222.1 | 0.16 | RiPP | acidocin B | Lactobacillus acidophilus |
| LMGH95 | BGC0000491.1 | 0.16 | RiPP | gassericin A | Lactobacillus gasseri |
| LMGH95 | BGC0000558.1 | 0.15 | RiPP | sublancin 168 | Bacillus subtilis subsp. subtilis str. 168 |
| LMGH95 | BGC0001862.1 | 0.15 | RiPP | geocillicin | Aeribacillus pallidus |
| LMGH95 | BGC0001930.1 | 0.15 | RiPP | pallidocin | Aeribacillus pallidus |
| LMGH95 | BGC0000626.1 | 0.15 | RiPP | thuricin | Bacillus thuringiensis |
| LMGH95 | BGC0001861.1 | 0.15 | RiPP | bacillicin BAG2O | Bacillus cereus BAG2O-1 |
| LMGH97 | BGC0000487.1 | 0.17 | RiPP | carnocyclin | Carnobacterium maltaromaticum |
| LMGH97 | BGC0001526.1 | 0.16 | Other | bartolosides E, bartolosides F, bartolosides G, bartolosides H, bartolosides I, bartolosides J, bartolosides K | Synechocystis salina LEGE 06099 |
| LMGH97 | BGC0001525.1 | 0.16 | Other | bartoloside 2, bartoloside 3, bartoloside 4 | Synechocystis salina LEGE 06155 |
| LMGH97 | BGC0001210.1 | 0.16 | RiPP | pseudomycoicidin | Bacillus pseudomycoides DSM 12442 |
| LMGH97 | BGC0001927.1 | 0.16 | Other | A-94964 | Streptomyces sp. |
| LMGH97 | BGC0001343.1 | 0.16 | Polyketide | aurachin A, aurachin D | Stigmatella aurantiaca Sg a15 |
| LMGH97 | BGC0000554.1 | 0.16 | RiPP | SRO15-3108 | Streptomyces filamentosus NRRL 15998 |
| LMGH97 | BGC0001407.1 | 0.16 | RiPP | bicereucin | Bacillus cereus SJ1 |
| LMGH97 | BGC0000719.1 | 0.16 | Saccharide | tobramycin | Streptoalloteichus hindustanus |
| LMGH97 | BGC0001779.1 | 0.16 | RiPP | siamycin | Streptomyces nodosus |
| LMGTW1 | BGC0001120.1 | 0.16 | NRP, Polyketide | burkholderic acid | Burkholderia thailandensis E264 |
| LMGTW1 | BGC0000719.1 | 0.16 | Saccharide | tobramycin | Streptoalloteichus hindustanus |
| LMGTW1 | BGC0001739.1 | 0.16 | Other | phosphonoacetic Acid | Streptomyces sp. NRRL F-525 |
| LMGTW1 | BGC0000554.1 | 0.16 | RiPP | SRO15-3108 | Streptomyces filamentosus NRRL 15998 |
| LMGTW1 | BGC0000720.1 | 0.16 | Saccharide | tobramycin | Streptoalloteichus tenebrarius |
| LMGTW1 | BGC0001104.1 | 0.16 | NRP, Polyketide | myxovirescin A1 | Myxococcus xanthus DK 1622 |
| LMGTW1 | BGC0001591.1 | 0.14 | Other | fatty acid enol ester | uncultured bacterium CSLC2 |
| LMGTW1 | BGC0001688.1 | 0.14 | Other | N-tetradecanoyl tyrosine | uncultured bacterium CSLC2 |
| LMGTW1 | BGC0001927.1 | 0.14 | Other | A-94964 | Streptomyces sp. |
| LMGTW1 | BGC0001850.1 | 0.14 | Other (Shikimate-derived) | diazaquinomycin A, diazaquinomycin E, diazaquinomycin F, diazaquinomycin G | Streptomyces sp. F001 |
| LMGTW3 | BGC0001120.1 | 0.16 | NRP, Polyketide | burkholderic acid | Burkholderia thailandensis E264 |
| LMGTW3 | BGC0000719.1 | 0.16 | Saccharide | tobramycin | Streptoalloteichus hindustanus |
| LMGTW3 | BGC0000554.1 | 0.16 | RiPP | SRO15-3108 | Streptomyces filamentosus NRRL 15998 |
| LMGTW3 | BGC0000720.1 | 0.16 | Saccharide | tobramycin | Streptoalloteichus tenebrarius |
| LMGTW3 | BGC0001850.1 | 0.14 | Other (Shikimate-derived) | diazaquinomycin A, diazaquinomycin E, diazaquinomycin F, diazaquinomycin G | Streptomyces sp. F001 |
| LMGTW3 | BGC0000783.1 | 0.14 | Saccharide | O-antigen | Xanthomonas oryzae pv. oryzae |
| LMGTW3 | BGC0000243.1 | 0.13 | Polyketide | macrotetrolide | Streptomyces griseus subsp. griseus |
| LMGTW3 | BGC0001540.1 | 0.13 | Other | CC-1065 | Streptomyces zelensis |
| LMGTW3 | BGC0000785.1 | 0.13 | Saccharide | O-antigen | Burkholderia mallei |
| LMGTW3 | BGC0000782.1 | 0.13 | Saccharide | O-antigen | Burkholderia pseudomallei 1026b |
| LMGTW6 | BGC0000554.1 | 0.21 | RiPP | SRO15-3108 | Streptomyces filamentosus NRRL 15998 |
| LMGTW6 | BGC0001120.1 | 0.16 | NRP, Polyketide | burkholderic acid | Burkholderia thailandensis E264 |
| LMGTW6 | BGC0000617.1 | 0.16 | RiPP | coagulin | Bacillus coagulans |
| LMGTW6 | BGC0000719.1 | 0.16 | Saccharide | tobramycin | Streptoalloteichus hindustanus |
| LMGTW6 | BGC0000720.1 | 0.16 | Saccharide | tobramycin | Streptoalloteichus tenebrarius |
| LMGTW6 | BGC0000624.1 | 0.15 | RiPP | salivaricin CRL1328 α peptide, salivaricin CRL1328 β peptide | Lactobacillus salivarius |
| LMGTW6 | BGC0000523.1 | 0.14 | RiPP | lactocin S | Lactobacillus sakei |
| LMGTW6 | BGC0001927.1 | 0.14 | Other | A-94964 | Streptomyces sp. |
| LMGTW6 | BGC0001850.1 | 0.14 | Other (Shikimate-derived) | diazaquinomycin A, diazaquinomycin E, diazaquinomycin F, diazaquinomycin G | Streptomyces sp. F001 |
| LMGTW6 | BGC0000783.1 | 0.14 | Saccharide | O-antigen | Xanthomonas oryzae pv. oryzae |
| LMGTW8 | BGC0000523.1 | 0.18 | RiPP | lactocin S | Lactobacillus sakei |
| LMGTW8 | BGC0001526.1 | 0.16 | Other | bartolosides E, bartolosides F, bartolosides G, bartolosides H, bartolosides I, bartolosides J, bartolosides K | Synechocystis salina LEGE 06099 |
| LMGTW8 | BGC0001525.1 | 0.16 | Other | bartoloside 2, bartoloside 3, bartoloside 4 | Synechocystis salina LEGE 06155 |
| LMGTW8 | BGC0001210.1 | 0.16 | RiPP | pseudomycoicidin | Bacillus pseudomycoides DSM 12442 |
| LMGTW8 | BGC0000554.1 | 0.16 | RiPP | SRO15-3108 | Streptomyces filamentosus NRRL 15998 |
| LMGTW8 | BGC0001407.1 | 0.16 | RiPP | bicereucin | Bacillus cereus SJ1 |
| LMGTW8 | BGC0000719.1 | 0.16 | Saccharide | tobramycin | Streptoalloteichus hindustanus |
| LMGTW8 | BGC0000519.1 | 0.16 | RiPP | labyrinthopeptin A2, labyrinthopeptin A1, labyrinthopeptin A3 | Actinomadura namibiensis |
| LMGTW8 | BGC0001551.1 | 0.16 | RiPP | citrulassin E | Streptomyces glaucescens |
| LMGTW8 | BGC0000521.1 | 0.16 | RiPP | lacticin 481 | Lactococcus lactis subsp. lactis |
| LN02 | BGC0001962.1 | 0.25 | Polyketide | hierridin B, hierridin C | Cyanobium sp. LEGE 06113 |
| LN02 | BGC0000761.1 | 0.18 | Saccharide | exopolysaccharide | Methylobacillus sp. 12S |
| LN02 | BGC0000780.1 | 0.18 | Saccharide | O&K-antigen | Vibrio parahaemolyticus |
| LN02 | BGC0000760.1 | 0.17 | Saccharide | emulsan | Acinetobacter venetianus RAG-1 = CIP 110063 |
| LN02 | BGC0000668.1 | 0.17 | Terpene, Alkaloid | 12-epi-hapalindole C isonitrile, 12-epi-hapalindole E, 12-epi-fischerindole U isonitrile, fischerindole L, 12-epi-fischerindole I isonitrile, welwitindolinone A isonitrile, welwitindolinone B isothiocyanate, welwitindolinone C isothiocyanate, N-methylwelwitindolinone C isothiocyanate, N-methylwelwitinsolinone C isonitrile, 3-epi-welwitindolinone B isothiocyanate, 3-(Z-2'-isocyanoethenyl)-indole | Hapalosiphon welwitschii UTEX B 1830 |
| LN02 | BGC0000928.1 | 0.17 | Other | T3 toxin | Cylindrospermopsis raciborskii T3 |
| LN02 | BGC0000188.1 | 0.17 | Alkaloid | saxitoxin, neosaxitoxin, decarbamoylsaxitoxin, gonyautoxin 3, gonyautoxin 2 | Cylindrospermopsis raciborskii T3 |
| LN02 | BGC0001947.1 | 0.17 | Saccharide | K53 capsular polysaccharide | Acinetobacter baumannii |
| LN02 | BGC0001765.1 | 0.17 | Saccharide | salecan | Agrobacterium tumefaciens |
| LN02 | BGC0001380.1 | 0.17 | Saccharide | salecan | Agrobacterium sp. ZX09 |
| LN12 | BGC0001120.1 | 0.16 | NRP, Polyketide | burkholderic acid | Burkholderia thailandensis E264 |
| LN12 | BGC0000719.1 | 0.16 | Saccharide | tobramycin | Streptoalloteichus hindustanus |
| LN12 | BGC0000554.1 | 0.16 | RiPP | SRO15-3108 | Streptomyces filamentosus NRRL 15998 |
| LN12 | BGC0000720.1 | 0.16 | Saccharide | tobramycin | Streptoalloteichus tenebrarius |
| LN12 | BGC0001104.1 | 0.16 | NRP, Polyketide | myxovirescin A1 | Myxococcus xanthus DK 1622 |
| LN12 | BGC0001521.1 | 0.14 | NRP | auriculamide | Herpetosiphon aurantiacus DSM 785 |
| LN12 | BGC0001927.1 | 0.14 | Other | A-94964 | Streptomyces sp. |
| LN12 | BGC0001850.1 | 0.14 | Other (Shikimate-derived) | diazaquinomycin A, diazaquinomycin E, diazaquinomycin F, diazaquinomycin G | Streptomyces sp. F001 |
| LN12 | BGC0000783.1 | 0.14 | Saccharide | O-antigen | Xanthomonas oryzae pv. oryzae |
| LN12 | BGC0000709.1 | 0.13 | Saccharide | neomycin | Streptomyces fradiae ATCC 10745 = DSM 40063 |
| LN23 | BGC0000888.1 | 0.16 | Other | bacilysin | Bacillus sp. CS93 |
| LN23 | BGC0001120.1 | 0.16 | NRP, Polyketide | burkholderic acid | Burkholderia thailandensis E264 |
| LN23 | BGC0000719.1 | 0.16 | Saccharide | tobramycin | Streptoalloteichus hindustanus |
| LN23 | BGC0000720.1 | 0.16 | Saccharide | tobramycin | Streptoalloteichus tenebrarius |
| LN23 | BGC0000554.1 | 0.16 | RiPP | SRO15-3108 | Streptomyces filamentosus NRRL 15998 |
| LN23 | BGC0001928.1 | 0.15 | Other | (2R,3S,4S)-5-fluoro-2,3,4-trihydroxypentanoic acid | Streptomyces sp. MA37 |
| LN23 | BGC0002008.1 | 0.15 | Other | aryl polyenes | Xenorhabdus doucetiae |
| LN23 | BGC0001591.1 | 0.14 | Other | fatty acid enol ester | uncultured bacterium CSLC2 |
| LN23 | BGC0001688.1 | 0.14 | Other | N-tetradecanoyl tyrosine | uncultured bacterium CSLC2 |
| LN23 | BGC0000836.1 | 0.14 | Other | APE Ec | Escherichia coli CFT073 |
| MGBC116435 | BGC0001594.1 | 0.26 | Alkaloid | fischerindole L | Fischerella muscicola UTEX 1829 |
| MGBC116435 | BGC0000668.1 | 0.24 | Terpene, Alkaloid | 12-epi-hapalindole C isonitrile, 12-epi-hapalindole E, 12-epi-fischerindole U isonitrile, fischerindole L, 12-epi-fischerindole I isonitrile, welwitindolinone A isonitrile, welwitindolinone B isothiocyanate, welwitindolinone C isothiocyanate, N-methylwelwitindolinone C isothiocyanate, N-methylwelwitinsolinone C isonitrile, 3-epi-welwitindolinone B isothiocyanate, 3-(Z-2'-isocyanoethenyl)-indole | Hapalosiphon welwitschii UTEX B 1830 |
| MGBC116435 | BGC0001595.1 | 0.22 | Alkaloid | fischerindole | Fischerella sp. SAG 46.79 |
| MGBC116435 | BGC0001126.1 | 0.21 | Terpene, Alkaloid | 12-epi-hapalindole J isonitrile, ambiguine A isonitrile, ambiguine B isonitrile, ambiguine C isonitrile, ambiguine D isonitrile, ambiguine E isonitrile, ambiguine K isonitrile, ambiguine L isonitrile, ambiguine I isonitrile, ambiguine J isonitrile | Fischerella ambigua UTEX 1903 |
| MGBC116435 | BGC0001612.1 | 0.21 | Alkaloid | ambiguine H isonitrile | Fischerella ambigua UTEX 1903 |
| MGBC116435 | BGC0001501.1 | 0.21 | Alkaloid | ambiguine P | Fischerella sp. TAU |
| MGBC116435 | BGC0001120.1 | 0.16 | NRP, Polyketide | burkholderic acid | Burkholderia thailandensis E264 |
| MGBC116435 | BGC0001664.1 | 0.16 | Terpene | merosterol | Scytonema sp. PCC 10023 |
| MGBC116435 | BGC0000554.1 | 0.16 | RiPP | SRO15-3108 | Streptomyces filamentosus NRRL 15998 |
| MGBC116435 | BGC0002009.1 | 0.15 | Polyketide | kanglemycin A, kanglemycin V1, kanglemycin V2 | Amycolatopsis vancoresmycina |
| NCDO 768 | BGC0001594.1 | 0.26 | Alkaloid | fischerindole L | Fischerella muscicola UTEX 1829 |
| NCDO 768 | BGC0000668.1 | 0.24 | Terpene, Alkaloid | 12-epi-hapalindole C isonitrile, 12-epi-hapalindole E, 12-epi-fischerindole U isonitrile, fischerindole L, 12-epi-fischerindole I isonitrile, welwitindolinone A isonitrile, welwitindolinone B isothiocyanate, welwitindolinone C isothiocyanate, N-methylwelwitindolinone C isothiocyanate, N-methylwelwitinsolinone C isonitrile, 3-epi-welwitindolinone B isothiocyanate, 3-(Z-2'-isocyanoethenyl)-indole | Hapalosiphon welwitschii UTEX B 1830 |
| NCDO 768 | BGC0001595.1 | 0.22 | Alkaloid | fischerindole | Fischerella sp. SAG 46.79 |
| NCDO 768 | BGC0001126.1 | 0.21 | Terpene, Alkaloid | 12-epi-hapalindole J isonitrile, ambiguine A isonitrile, ambiguine B isonitrile, ambiguine C isonitrile, ambiguine D isonitrile, ambiguine E isonitrile, ambiguine K isonitrile, ambiguine L isonitrile, ambiguine I isonitrile, ambiguine J isonitrile | Fischerella ambigua UTEX 1903 |
| NCDO 768 | BGC0001612.1 | 0.21 | Alkaloid | ambiguine H isonitrile | Fischerella ambigua UTEX 1903 |
| NCDO 768 | BGC0001501.1 | 0.21 | Alkaloid | ambiguine P | Fischerella sp. TAU |
| NCDO 768 | BGC0001120.1 | 0.16 | NRP, Polyketide | burkholderic acid | Burkholderia thailandensis E264 |
| NCDO 768 | BGC0000719.1 | 0.16 | Saccharide | tobramycin | Streptoalloteichus hindustanus |
| NCDO 768 | BGC0000720.1 | 0.16 | Saccharide | tobramycin | Streptoalloteichus tenebrarius |
| NCDO 768 | BGC0002009.1 | 0.16 | Polyketide | kanglemycin A, kanglemycin V1, kanglemycin V2 | Amycolatopsis vancoresmycina |
| PS12 | BGC0001937.1 | 0.17 | Other | tubercidin | Streptomyces tubercidicus |
| PS12 | BGC0001120.1 | 0.16 | NRP, Polyketide | burkholderic acid | Burkholderia thailandensis E264 |
| PS12 | BGC0001591.1 | 0.16 | Other | fatty acid enol ester | uncultured bacterium CSLC2 |
| PS12 | BGC0001688.1 | 0.16 | Other | N-tetradecanoyl tyrosine | uncultured bacterium CSLC2 |
| PS12 | BGC0000719.1 | 0.16 | Saccharide | tobramycin | Streptoalloteichus hindustanus |
| PS12 | BGC0000554.1 | 0.16 | RiPP | SRO15-3108 | Streptomyces filamentosus NRRL 15998 |
| PS12 | BGC0000720.1 | 0.16 | Saccharide | tobramycin | Streptoalloteichus tenebrarius |
| PS12 | BGC0001927.1 | 0.15 | Other | A-94964 | Streptomyces sp. |
| PS12 | BGC0001808.1 | 0.15 | Other | toyocamycin | Streptomyces diastatochromogenes |
| PS12 | BGC0000881.1 | 0.15 | Other | toyocamycin | Streptomyces ahygroscopicus subsp. wuzhouensis |
| TMW21073 | BGC0001594.1 | 0.26 | Alkaloid | fischerindole L | Fischerella muscicola UTEX 1829 |
| TMW21073 | BGC0000668.1 | 0.24 | Terpene, Alkaloid | 12-epi-hapalindole C isonitrile, 12-epi-hapalindole E, 12-epi-fischerindole U isonitrile, fischerindole L, 12-epi-fischerindole I isonitrile, welwitindolinone A isonitrile, welwitindolinone B isothiocyanate, welwitindolinone C isothiocyanate, N-methylwelwitindolinone C isothiocyanate, N-methylwelwitinsolinone C isonitrile, 3-epi-welwitindolinone B isothiocyanate, 3-(Z-2'-isocyanoethenyl)-indole | Hapalosiphon welwitschii UTEX B 1830 |
| TMW21073 | BGC0001595.1 | 0.22 | Alkaloid | fischerindole | Fischerella sp. SAG 46.79 |
| TMW21073 | BGC0001126.1 | 0.21 | Terpene, Alkaloid | 12-epi-hapalindole J isonitrile, ambiguine A isonitrile, ambiguine B isonitrile, ambiguine C isonitrile, ambiguine D isonitrile, ambiguine E isonitrile, ambiguine K isonitrile, ambiguine L isonitrile, ambiguine I isonitrile, ambiguine J isonitrile | Fischerella ambigua UTEX 1903 |
| TMW21073 | BGC0001612.1 | 0.21 | Alkaloid | ambiguine H isonitrile | Fischerella ambigua UTEX 1903 |
| TMW21073 | BGC0001501.1 | 0.21 | Alkaloid | ambiguine P | Fischerella sp. TAU |
| TMW21073 | BGC0001120.1 | 0.16 | NRP, Polyketide | burkholderic acid | Burkholderia thailandensis E264 |
| TMW21073 | BGC0000719.1 | 0.16 | Saccharide | tobramycin | Streptoalloteichus hindustanus |
| TMW21073 | BGC0000720.1 | 0.16 | Saccharide | tobramycin | Streptoalloteichus tenebrarius |
| TMW21073 | BGC0002009.1 | 0.16 | Polyketide | kanglemycin A, kanglemycin V1, kanglemycin V2 | Amycolatopsis vancoresmycina |
| TMW21195 | BGC0001594.1 | 0.26 | Alkaloid | fischerindole L | Fischerella muscicola UTEX 1829 |
| TMW21195 | BGC0001595.1 | 0.25 | Alkaloid | fischerindole | Fischerella sp. SAG 46.79 |
| TMW21195 | BGC0000668.1 | 0.24 | Terpene, Alkaloid | 12-epi-hapalindole C isonitrile, 12-epi-hapalindole E, 12-epi-fischerindole U isonitrile, fischerindole L, 12-epi-fischerindole I isonitrile, welwitindolinone A isonitrile, welwitindolinone B isothiocyanate, welwitindolinone C isothiocyanate, N-methylwelwitindolinone C isothiocyanate, N-methylwelwitinsolinone C isonitrile, 3-epi-welwitindolinone B isothiocyanate, 3-(Z-2'-isocyanoethenyl)-indole | Hapalosiphon welwitschii UTEX B 1830 |
| TMW21195 | BGC0001126.1 | 0.21 | Terpene, Alkaloid | 12-epi-hapalindole J isonitrile, ambiguine A isonitrile, ambiguine B isonitrile, ambiguine C isonitrile, ambiguine D isonitrile, ambiguine E isonitrile, ambiguine K isonitrile, ambiguine L isonitrile, ambiguine I isonitrile, ambiguine J isonitrile | Fischerella ambigua UTEX 1903 |
| TMW21195 | BGC0001612.1 | 0.21 | Alkaloid | ambiguine H isonitrile | Fischerella ambigua UTEX 1903 |
| TMW21195 | BGC0001501.1 | 0.21 | Alkaloid | ambiguine P | Fischerella sp. TAU |
| TMW21195 | BGC0001120.1 | 0.16 | NRP, Polyketide | burkholderic acid | Burkholderia thailandensis E264 |
| TMW21195 | BGC0000719.1 | 0.16 | Saccharide | tobramycin | Streptoalloteichus hindustanus |
| TMW21195 | BGC0000720.1 | 0.16 | Saccharide | tobramycin | Streptoalloteichus tenebrarius |
| TMW21195 | BGC0002009.1 | 0.16 | Polyketide | kanglemycin A, kanglemycin V1, kanglemycin V2 | Amycolatopsis vancoresmycina |
| TMW21195 | BGC0000500.1 | 0.16 | RiPP | carnolysin A1, carnolysin A2 | Carnobacterium maltaromaticum |
| TMW21195 | BGC0000554.1 | 0.16 | RiPP | SRO15-3108 | Streptomyces filamentosus NRRL 15998 |
| TMW21195 | BGC0001639.1 | 0.16 | RiPP | klebsidin | Klebsiella pneumoniae |
| TMW21195 | BGC0000294.1 | 0.16 | NRP | acinetobactin | Acinetobacter baumannii |
| TMW21195 | BGC0000484.1 | 0.16 | RiPP | glycocin F | Lactobacillus plantarum |
| TMW21195 | BGC0000504.1 | 0.16 | RiPP | cytolysin ClyLl, cytolysin ClyLs | Plasmid pAD1 |
| TMW21195 | BGC0000548.1 | 0.16 | RiPP | salivaricin A | Streptococcus salivarius |
| TMW21195 | BGC0001891.1 | 0.15 | Other | closthioamide | Ruminiclostridium cellulolyticum H10 |
| TMW21195 | BGC0001120.1 | 0.15 | NRP, Polyketide | burkholderic acid | Burkholderia thailandensis E264 |
| TMW21195 | BGC0002009.1 | 0.15 | Polyketide | kanglemycin A, kanglemycin V1, kanglemycin V2 | Amycolatopsis vancoresmycina |
| TR070 | BGC0001209.1 | 0.11 | RiPP | streptide | Streptococcus thermophilus LMD-9 |
| TR070 | BGC0001929.1 | 0.07 | RiPP | WGK | Streptococcus ferus |
| TR070 | BGC0001594.1 | 0.26 | Alkaloid | fischerindole L | Fischerella muscicola UTEX 1829 |
| TR070 | BGC0000668.1 | 0.24 | Terpene, Alkaloid | 12-epi-hapalindole C isonitrile, 12-epi-hapalindole E, 12-epi-fischerindole U isonitrile, fischerindole L, 12-epi-fischerindole I isonitrile, welwitindolinone A isonitrile, welwitindolinone B isothiocyanate, welwitindolinone C isothiocyanate, N-methylwelwitindolinone C isothiocyanate, N-methylwelwitinsolinone C isonitrile, 3-epi-welwitindolinone B isothiocyanate, 3-(Z-2'-isocyanoethenyl)-indole | Hapalosiphon welwitschii UTEX B 1830 |
| TR070 | BGC0001595.1 | 0.22 | Alkaloid | fischerindole | Fischerella sp. SAG 46.79 |
| TR070 | BGC0001126.1 | 0.21 | Terpene, Alkaloid | 12-epi-hapalindole J isonitrile, ambiguine A isonitrile, ambiguine B isonitrile, ambiguine C isonitrile, ambiguine D isonitrile, ambiguine E isonitrile, ambiguine K isonitrile, ambiguine L isonitrile, ambiguine I isonitrile, ambiguine J isonitrile | Fischerella ambigua UTEX 1903 |
| TR070 | BGC0001612.1 | 0.21 | Alkaloid | ambiguine H isonitrile | Fischerella ambigua UTEX 1903 |
| TR070 | BGC0001501.1 | 0.21 | Alkaloid | ambiguine P | Fischerella sp. TAU |
| TR070 | BGC0001120.1 | 0.16 | NRP, Polyketide | burkholderic acid | Burkholderia thailandensis E264 |
| TR070 | BGC0001664.1 | 0.16 | Terpene | merosterol | Scytonema sp. PCC 10023 |
| TR070 | BGC0002009.1 | 0.15 | Polyketide | kanglemycin A, kanglemycin V1, kanglemycin V2 | Amycolatopsis vancoresmycina |
| TR070 | BGC0001591.1 | 0.14 | Other | fatty acid enol ester | uncultured bacterium CSLC2 |
| UBA11295 | BGC0001594.1 | 0.26 | Alkaloid | fischerindole L | Fischerella muscicola UTEX 1829 |
| UBA11295 | BGC0000668.1 | 0.24 | Terpene, Alkaloid | 12-epi-hapalindole C isonitrile, 12-epi-hapalindole E, 12-epi-fischerindole U isonitrile, fischerindole L, 12-epi-fischerindole I isonitrile, welwitindolinone A isonitrile, welwitindolinone B isothiocyanate, welwitindolinone C isothiocyanate, N-methylwelwitindolinone C isothiocyanate, N-methylwelwitinsolinone C isonitrile, 3-epi-welwitindolinone B isothiocyanate, 3-(Z-2'-isocyanoethenyl)-indole | Hapalosiphon welwitschii UTEX B 1830 |
| UBA11295 | BGC0001595.1 | 0.22 | Alkaloid | fischerindole | Fischerella sp. SAG 46.79 |
| UBA11295 | BGC0001126.1 | 0.21 | Terpene, Alkaloid | 12-epi-hapalindole J isonitrile, ambiguine A isonitrile, ambiguine B isonitrile, ambiguine C isonitrile, ambiguine D isonitrile, ambiguine E isonitrile, ambiguine K isonitrile, ambiguine L isonitrile, ambiguine I isonitrile, ambiguine J isonitrile | Fischerella ambigua UTEX 1903 |
| UBA11295 | BGC0001612.1 | 0.21 | Alkaloid | ambiguine H isonitrile | Fischerella ambigua UTEX 1903 |
| UBA11295 | BGC0001501.1 | 0.21 | Alkaloid | ambiguine P | Fischerella sp. TAU |
| UBA11295 | BGC0001120.1 | 0.16 | NRP, Polyketide | burkholderic acid | Burkholderia thailandensis E264 |
| UBA11295 | BGC0001664.1 | 0.16 | Terpene | merosterol | Scytonema sp. PCC 10023 |
| UBA11295 | BGC0000554.1 | 0.16 | RiPP | SRO15-3108 | Streptomyces filamentosus NRRL 15998 |
| UBA11295 | BGC0002009.1 | 0.15 | Polyketide | kanglemycin A, kanglemycin V1, kanglemycin V2 | Amycolatopsis vancoresmycina |


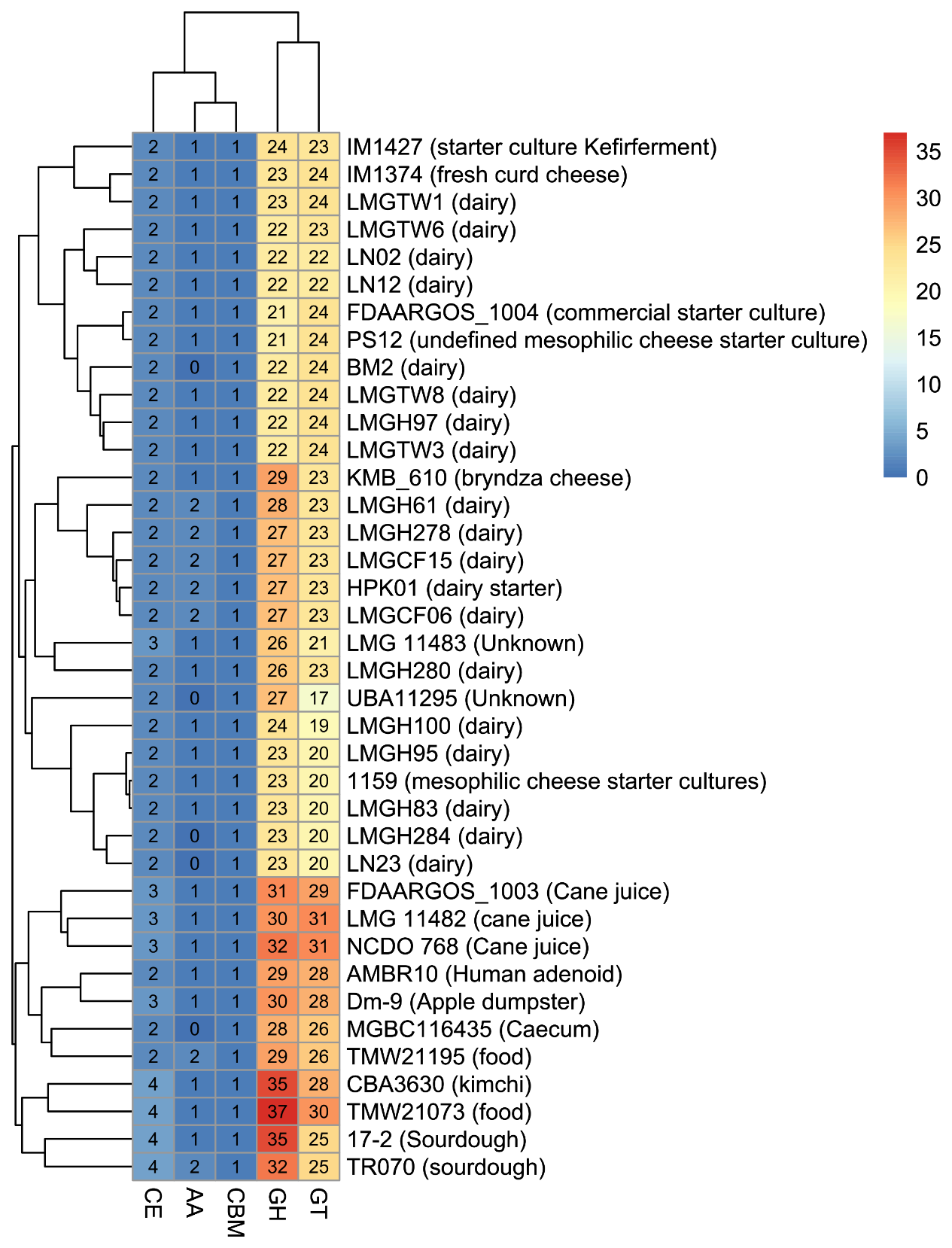


**Supplementary Figure 1.** Heatmap of CAZymes distribution and clustering across thirty-eight *Ln. pseudomesenteroides* genomes. The color gradient from lighter to darker colours represents the abundance of CAZymes found in each genome. GH: Glycoside hydrolase, GT: Glycosyltransferase, CE: Carbohydrate esterase, AA: Auxiliary activity, CBM: Carbohydrate binding module. Pheatmap package in R programming language (version 4.1.1) was used to draw the heatmap.
